# Supplementary figures and images for: Small RNAs from mitochondrial genome recombination sites are incorporated into T. gondii mitoribosomes
Source: eLife. 2024 Feb 16;13:e95407. doi: 10.7554/eLife.95407 (PMC10948144; doi:10.7554/eLife.95407)

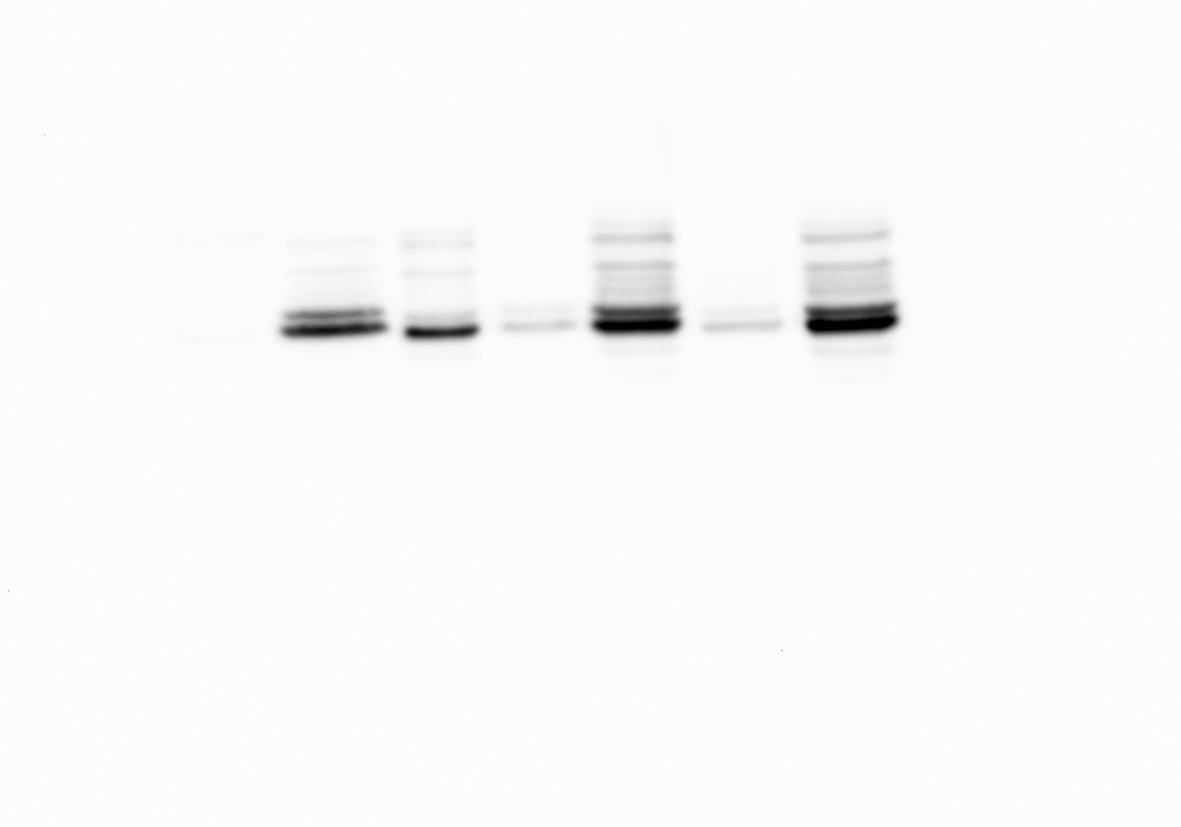

Supplement: Figure 1—source data 1. — Uncropped blots and gels accompanied by images indicating the areas shown in Figure 1B–D with a red rectangle. In addition, raw scan images are provided. If the scan contains multiple blots, the position of the blot of interest is indicated in the file name. Additionally, for immunoblots light image overlays depicting the membrane outline are provided. [file elife-95407-fig1-data1.zip › Fig 1-source data 1/Figure 1B- source data mGFP raw scan.tif]

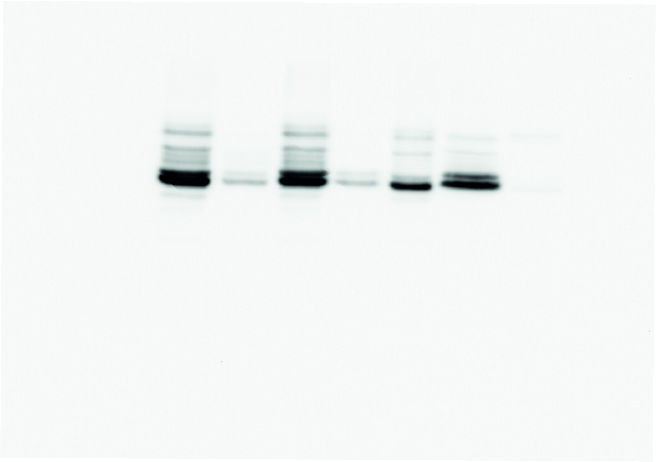

Supplement: Figure 1—source data 1. — Uncropped blots and gels accompanied by images indicating the areas shown in Figure 1B–D with a red rectangle. In addition, raw scan images are provided. If the scan contains multiple blots, the position of the blot of interest is indicated in the file name. Additionally, for immunoblots light image overlays depicting the membrane outline are provided. [file elife-95407-fig1-data1.zip › Fig 1-source data 1/Figure 1B-source data mGFP blot.jpg]

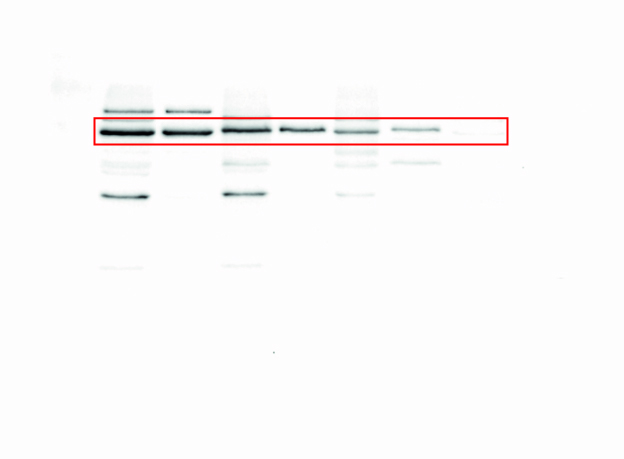

Supplement: Figure 1—source data 1. — Uncropped blots and gels accompanied by images indicating the areas shown in Figure 1B–D with a red rectangle. In addition, raw scan images are provided. If the scan contains multiple blots, the position of the blot of interest is indicated in the file name. Additionally, for immunoblots light image overlays depicting the membrane outline are provided. [file elife-95407-fig1-data1.zip › Fig 1-source data 1/Figure 1B-source data HSP70 blot labeled.jpg]

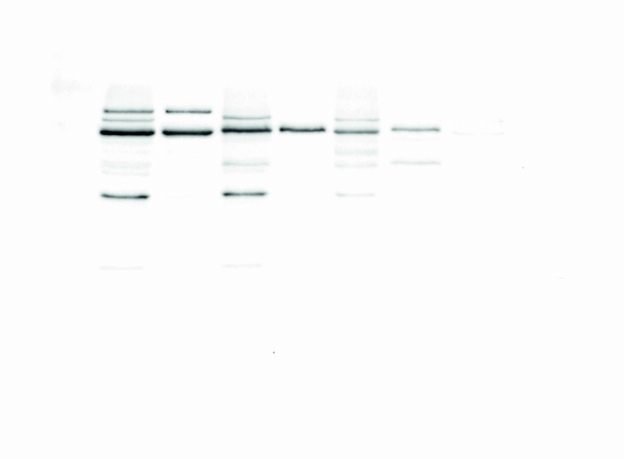

Supplement: Figure 1—source data 1. — Uncropped blots and gels accompanied by images indicating the areas shown in Figure 1B–D with a red rectangle. In addition, raw scan images are provided. If the scan contains multiple blots, the position of the blot of interest is indicated in the file name. Additionally, for immunoblots light image overlays depicting the membrane outline are provided. [file elife-95407-fig1-data1.zip › Fig 1-source data 1/Figure 1B-source data HSP70 blot.jpg]

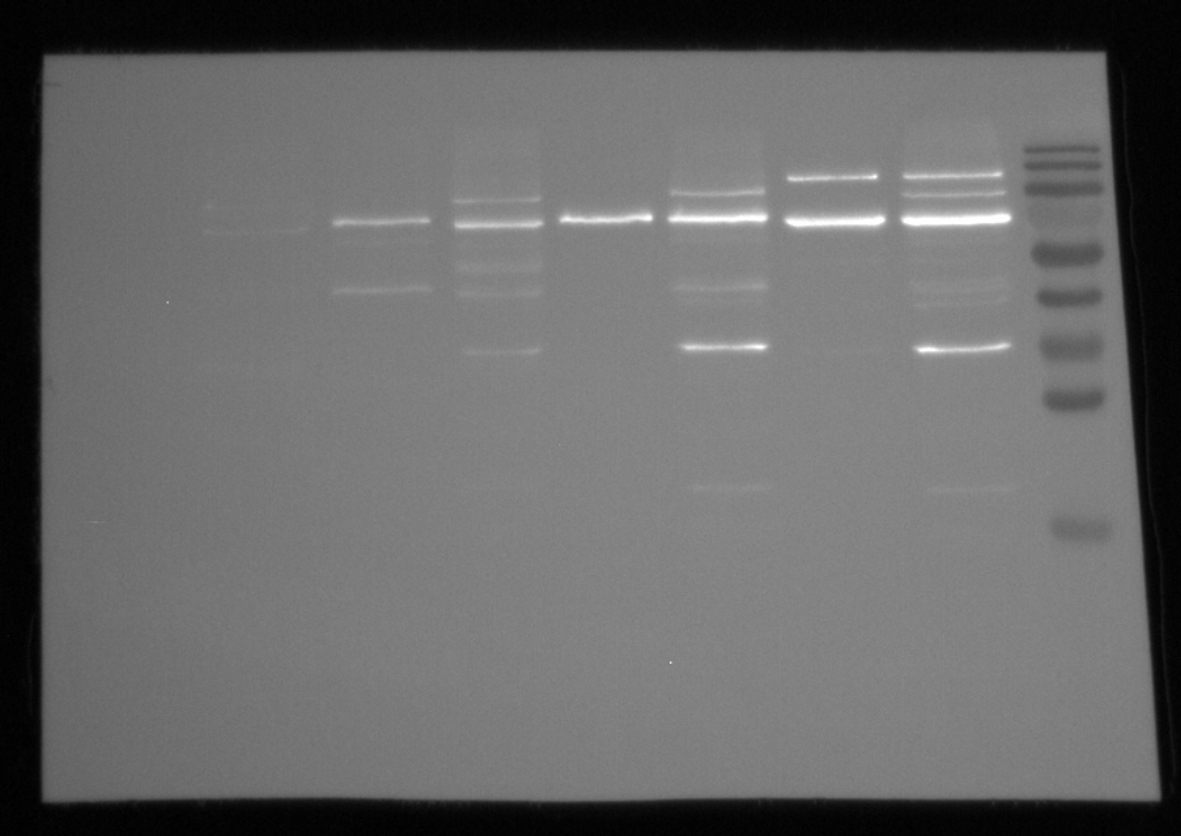

Supplement: Figure 1—source data 1. — Uncropped blots and gels accompanied by images indicating the areas shown in Figure 1B–D with a red rectangle. In addition, raw scan images are provided. If the scan contains multiple blots, the position of the blot of interest is indicated in the file name. Additionally, for immunoblots light image overlays depicting the membrane outline are provided. [file elife-95407-fig1-data1.zip › Fig 1-source data 1/Figure 1B-source data HSP70 raw scan light image overlay.tif]

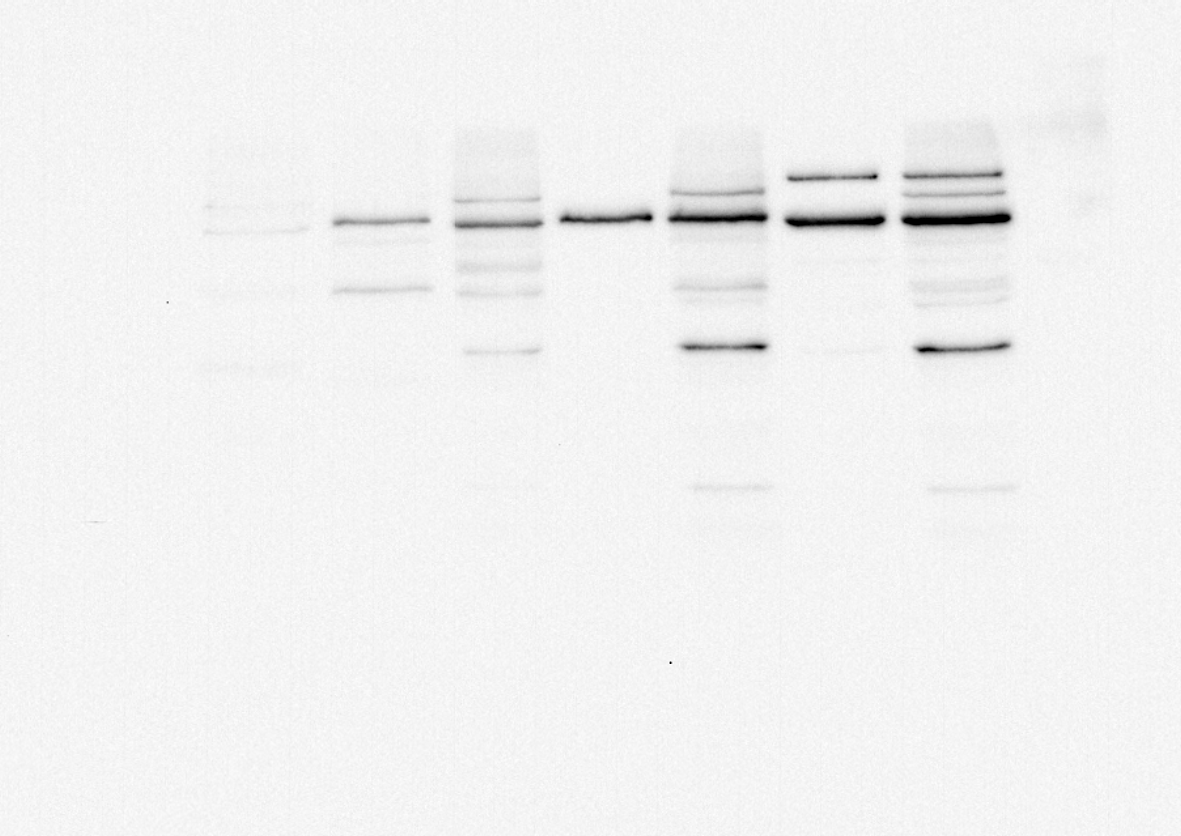

Supplement: Figure 1—source data 1. — Uncropped blots and gels accompanied by images indicating the areas shown in Figure 1B–D with a red rectangle. In addition, raw scan images are provided. If the scan contains multiple blots, the position of the blot of interest is indicated in the file name. Additionally, for immunoblots light image overlays depicting the membrane outline are provided. [file elife-95407-fig1-data1.zip › Fig 1-source data 1/Figure 1B-source data HSP70 raw scan.tif]

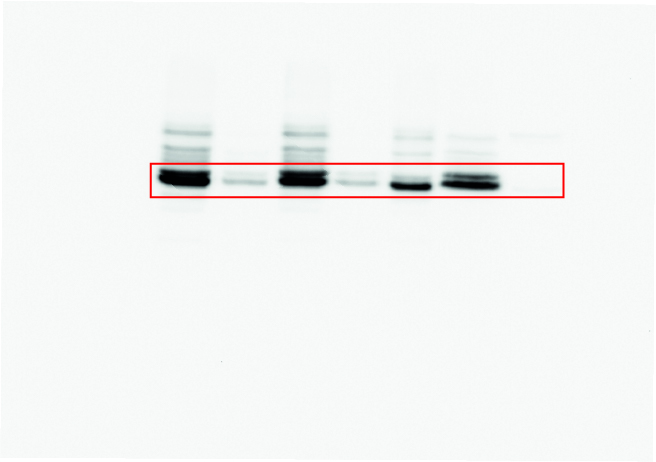

Supplement: Figure 1—source data 1. — Uncropped blots and gels accompanied by images indicating the areas shown in Figure 1B–D with a red rectangle. In addition, raw scan images are provided. If the scan contains multiple blots, the position of the blot of interest is indicated in the file name. Additionally, for immunoblots light image overlays depicting the membrane outline are provided. [file elife-95407-fig1-data1.zip › Fig 1-source data 1/Figure 1B-source data mGFP blot labeled.jpg]

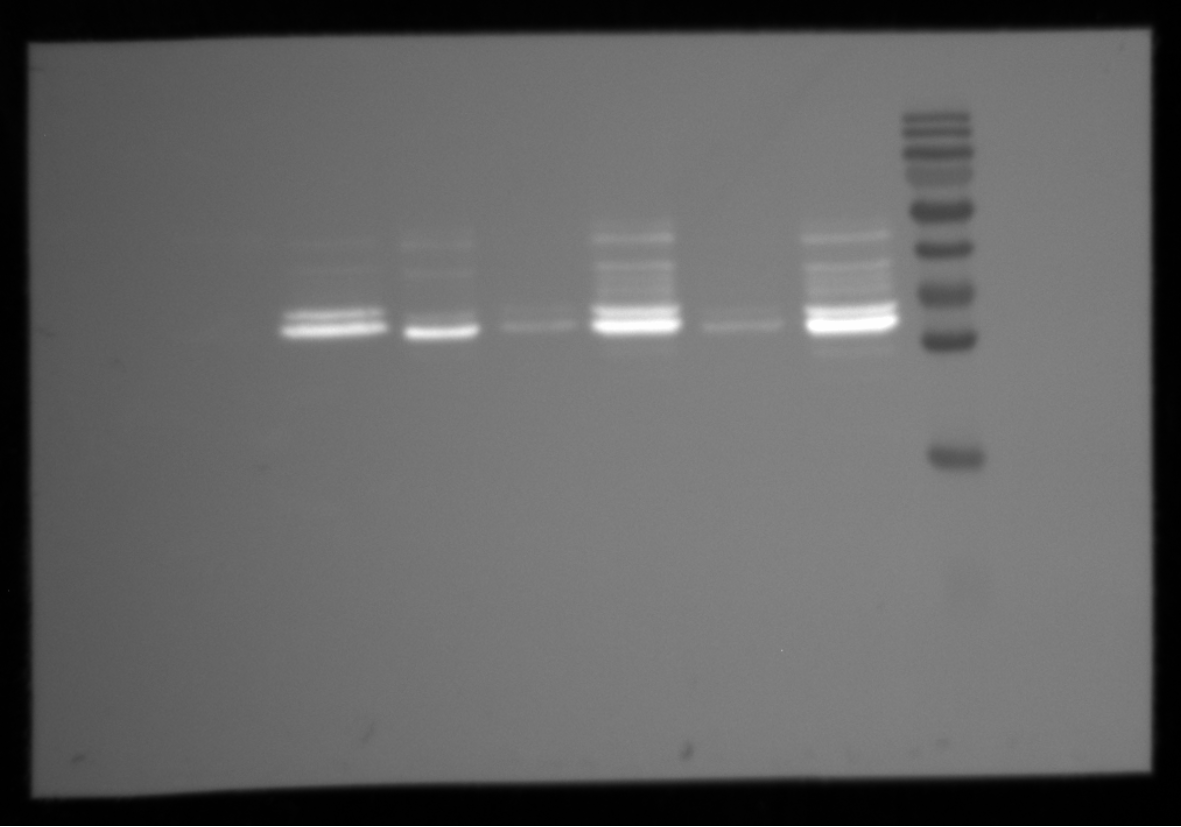

Supplement: Figure 1—source data 1. — Uncropped blots and gels accompanied by images indicating the areas shown in Figure 1B–D with a red rectangle. In addition, raw scan images are provided. If the scan contains multiple blots, the position of the blot of interest is indicated in the file name. Additionally, for immunoblots light image overlays depicting the membrane outline are provided. [file elife-95407-fig1-data1.zip › Fig 1-source data 1/Figure 1B-source data mGFP raw scan light image overlay.tif]

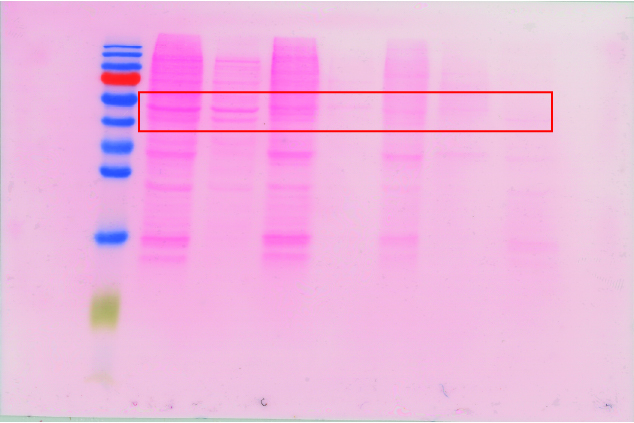

Supplement: Figure 1—source data 1. — Uncropped blots and gels accompanied by images indicating the areas shown in Figure 1B–D with a red rectangle. In addition, raw scan images are provided. If the scan contains multiple blots, the position of the blot of interest is indicated in the file name. Additionally, for immunoblots light image overlays depicting the membrane outline are provided. [file elife-95407-fig1-data1.zip › Fig 1-source data 1/Figure 1B-source data ponceau uncropped labeled.jpg]

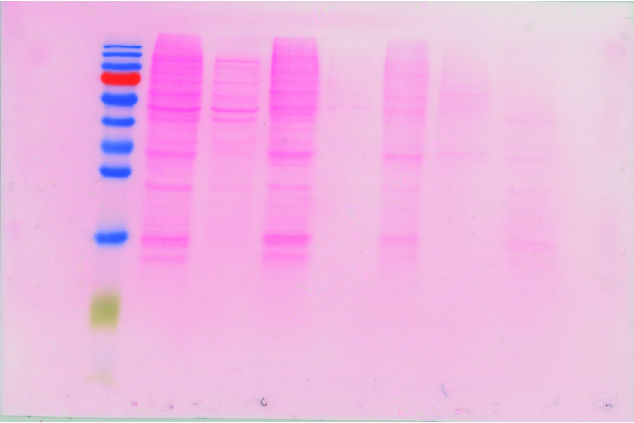

Supplement: Figure 1—source data 1. — Uncropped blots and gels accompanied by images indicating the areas shown in Figure 1B–D with a red rectangle. In addition, raw scan images are provided. If the scan contains multiple blots, the position of the blot of interest is indicated in the file name. Additionally, for immunoblots light image overlays depicting the membrane outline are provided. [file elife-95407-fig1-data1.zip › Fig 1-source data 1/Figure 1B-source data ponceau uncropped.jpg]

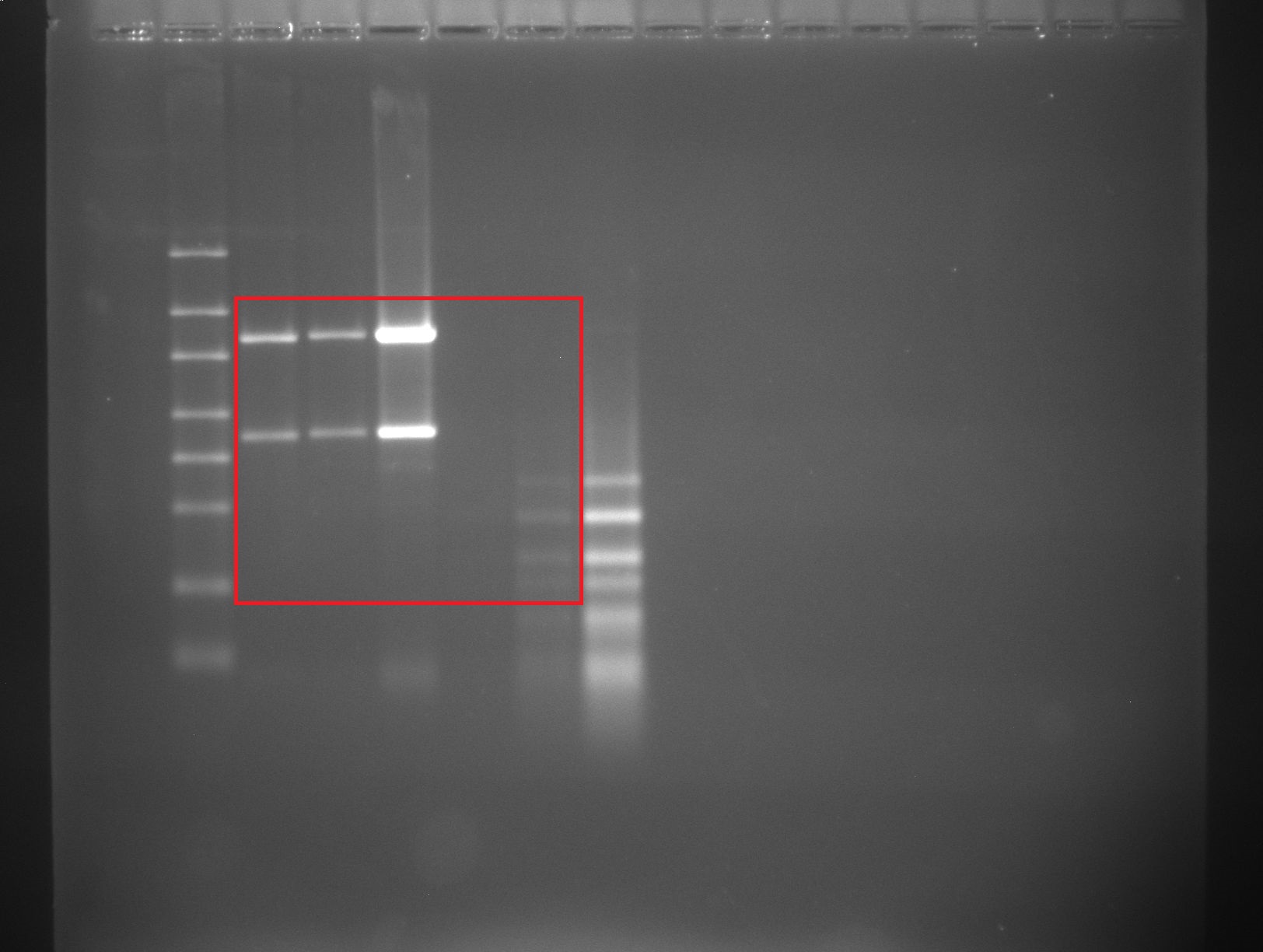

Supplement: Figure 1—source data 1. — Uncropped blots and gels accompanied by images indicating the areas shown in Figure 1B–D with a red rectangle. In addition, raw scan images are provided. If the scan contains multiple blots, the position of the blot of interest is indicated in the file name. Additionally, for immunoblots light image overlays depicting the membrane outline are provided. [file elife-95407-fig1-data1.zip › Fig 1-source data 1/Figure 1C-source data RNA gel uncropped labeled.jpg]

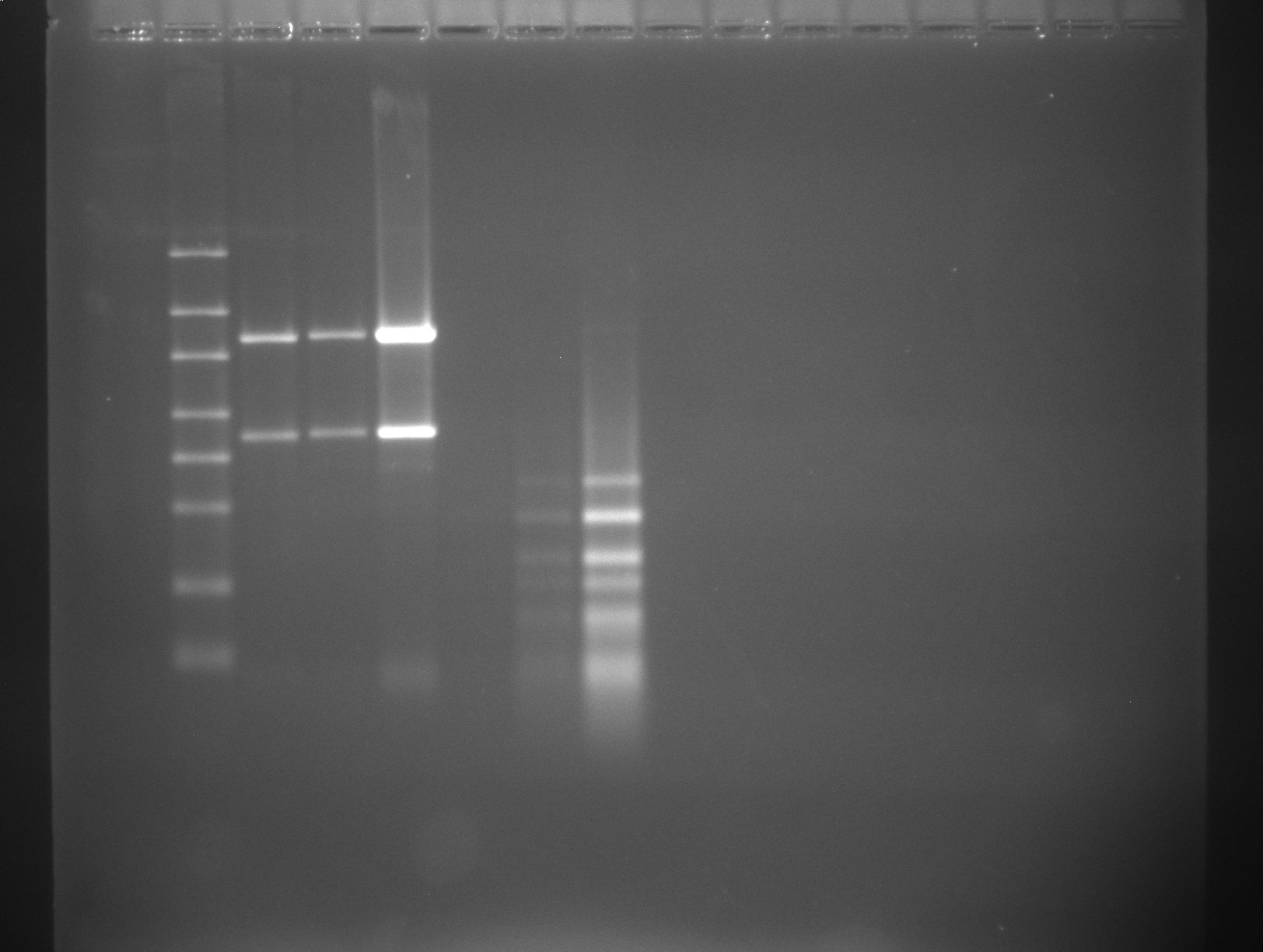

Supplement: Figure 1—source data 1. — Uncropped blots and gels accompanied by images indicating the areas shown in Figure 1B–D with a red rectangle. In addition, raw scan images are provided. If the scan contains multiple blots, the position of the blot of interest is indicated in the file name. Additionally, for immunoblots light image overlays depicting the membrane outline are provided. [file elife-95407-fig1-data1.zip › Fig 1-source data 1/Figure 1C-source data RNA gel uncropped.jpg]

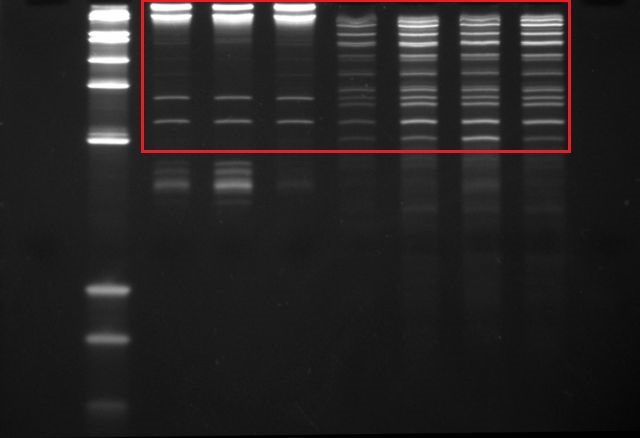

Supplement: Figure 1—source data 1. — Uncropped blots and gels accompanied by images indicating the areas shown in Figure 1B–D with a red rectangle. In addition, raw scan images are provided. If the scan contains multiple blots, the position of the blot of interest is indicated in the file name. Additionally, for immunoblots light image overlays depicting the membrane outline are provided. [file elife-95407-fig1-data1.zip › Fig 1-source data 1/Figure 1D-source data ethidiumbromide uncropped labeled.jpg]

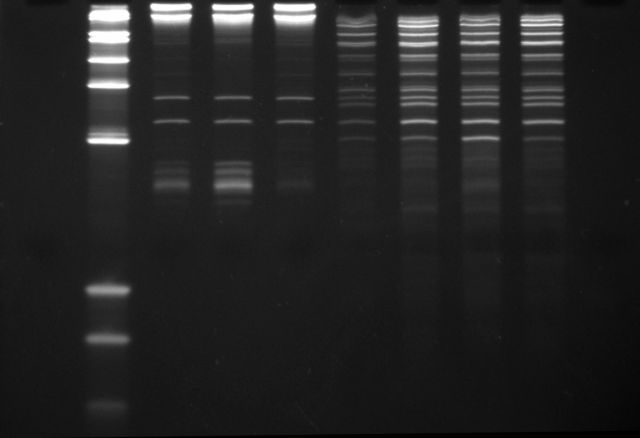

Supplement: Figure 1—source data 1. — Uncropped blots and gels accompanied by images indicating the areas shown in Figure 1B–D with a red rectangle. In addition, raw scan images are provided. If the scan contains multiple blots, the position of the blot of interest is indicated in the file name. Additionally, for immunoblots light image overlays depicting the membrane outline are provided. [file elife-95407-fig1-data1.zip › Fig 1-source data 1/Figure 1D-source data ethidiumbromide uncropped.jpg]

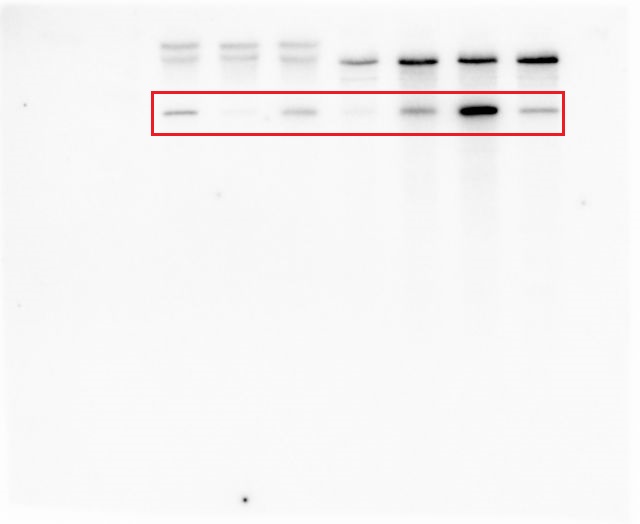

Supplement: Figure 1—source data 1. — Uncropped blots and gels accompanied by images indicating the areas shown in Figure 1B–D with a red rectangle. In addition, raw scan images are provided. If the scan contains multiple blots, the position of the blot of interest is indicated in the file name. Additionally, for immunoblots light image overlays depicting the membrane outline are provided. [file elife-95407-fig1-data1.zip › Fig 1-source data 1/Figure 1D-source data LSUF blot labeled.jpg]

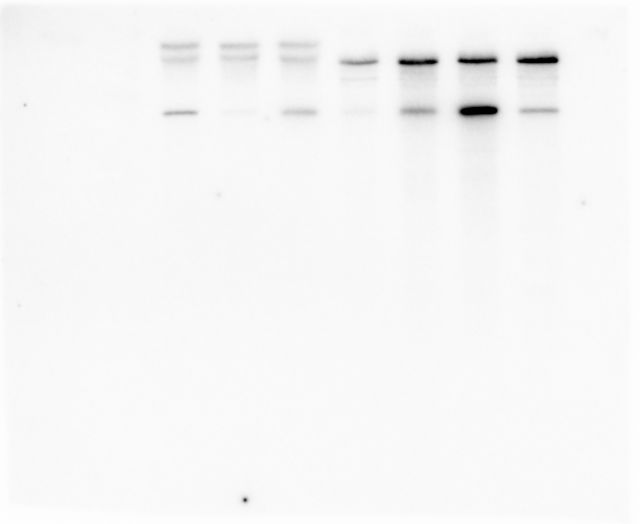

Supplement: Figure 1—source data 1. — Uncropped blots and gels accompanied by images indicating the areas shown in Figure 1B–D with a red rectangle. In addition, raw scan images are provided. If the scan contains multiple blots, the position of the blot of interest is indicated in the file name. Additionally, for immunoblots light image overlays depicting the membrane outline are provided. [file elife-95407-fig1-data1.zip › Fig 1-source data 1/Figure 1D-source data LSUF blot.jpg]

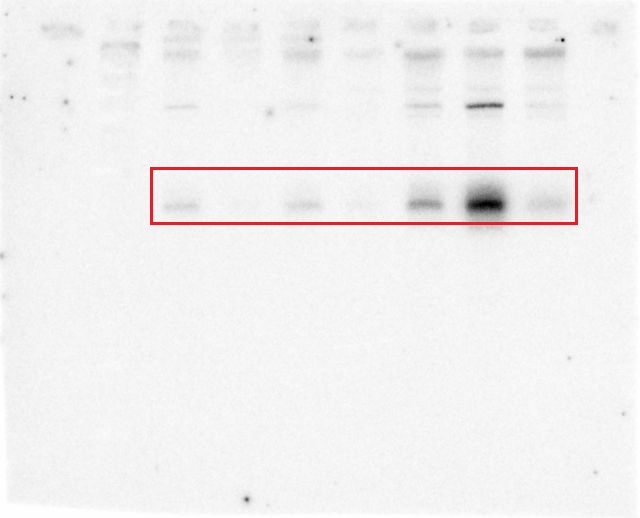

Supplement: Figure 1—source data 1. — Uncropped blots and gels accompanied by images indicating the areas shown in Figure 1B–D with a red rectangle. In addition, raw scan images are provided. If the scan contains multiple blots, the position of the blot of interest is indicated in the file name. Additionally, for immunoblots light image overlays depicting the membrane outline are provided. [file elife-95407-fig1-data1.zip › Fig 1-source data 1/Figure 1D-source data SSUD blot labeled.jpg]

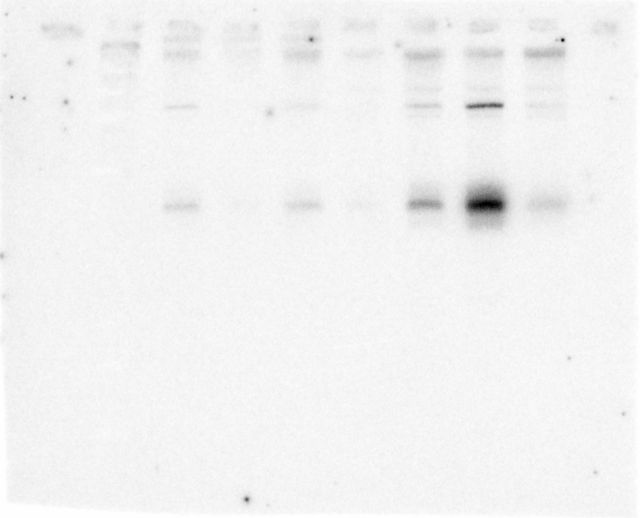

Supplement: Figure 1—source data 1. — Uncropped blots and gels accompanied by images indicating the areas shown in Figure 1B–D with a red rectangle. In addition, raw scan images are provided. If the scan contains multiple blots, the position of the blot of interest is indicated in the file name. Additionally, for immunoblots light image overlays depicting the membrane outline are provided. [file elife-95407-fig1-data1.zip › Fig 1-source data 1/Figure 1D-source data SSUD blot.jpg]

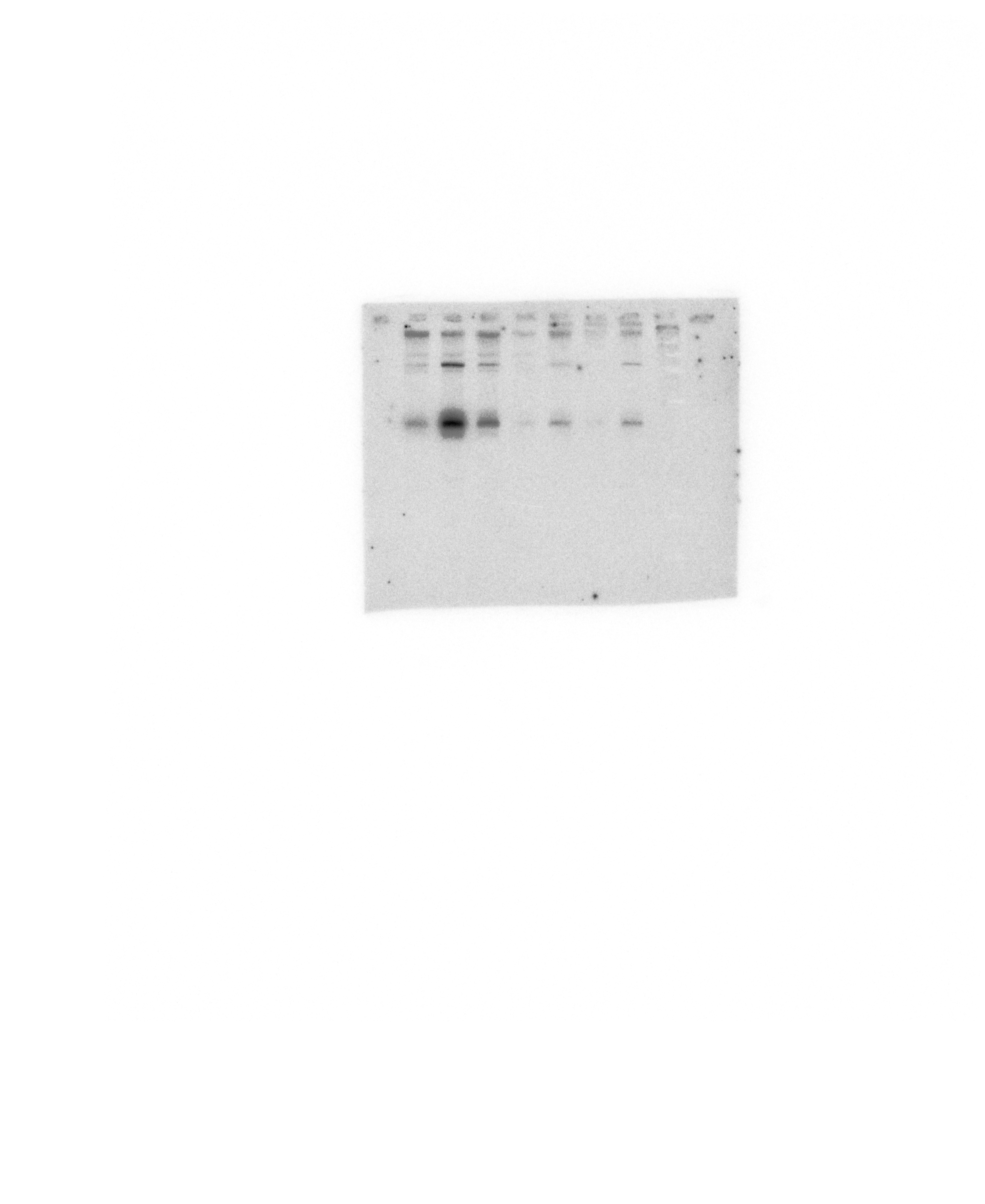

Supplement: Figure 1—source data 1. — Uncropped blots and gels accompanied by images indicating the areas shown in Figure 1B–D with a red rectangle. In addition, raw scan images are provided. If the scan contains multiple blots, the position of the blot of interest is indicated in the file name. Additionally, for immunoblots light image overlays depicting the membrane outline are provided. [file elife-95407-fig1-data1.zip › Fig 1-source data 1/Figure 1D-source data SSUD raw scan.tif]

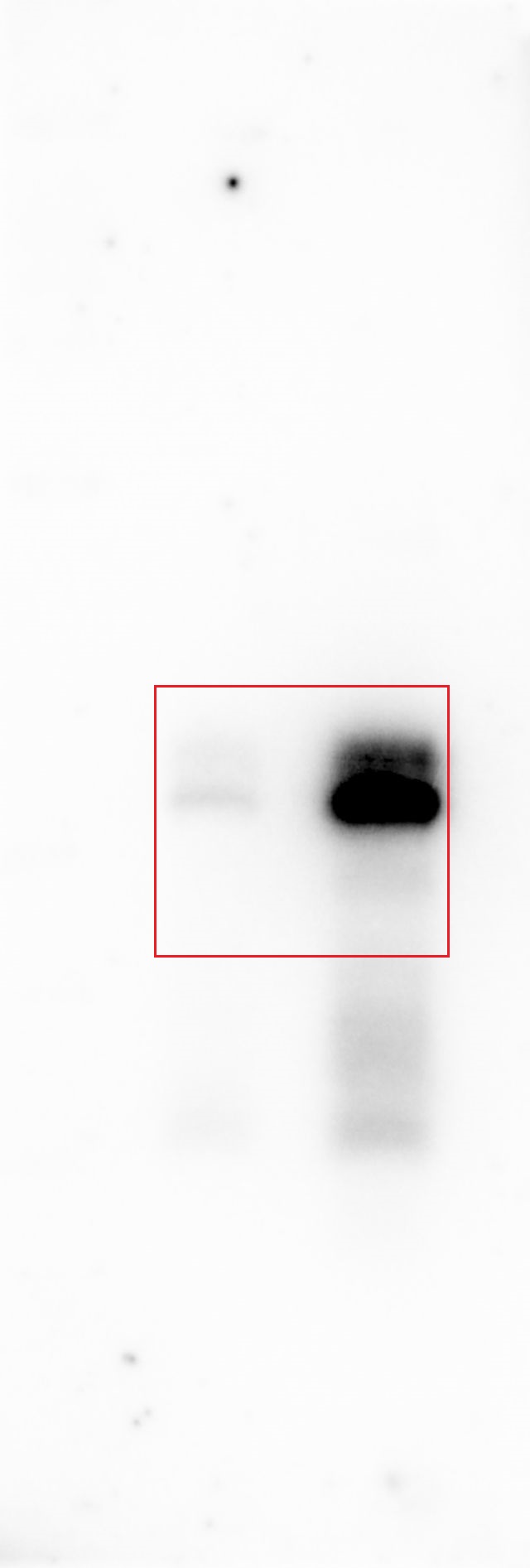

Supplement: Figure 3—source data 1. — Uncropped blots accompanied by images indicating the areas shown in Figure 3B with a red rectangle. In addition, raw scan images are provided. If the scan contains multiple blots, the position of the blot of interest is indicated in the file name. [file elife-95407-fig3-data1.zip › Fig 3-source data 1/Figure 3B-source data RNA17 blot labeled.jpg]

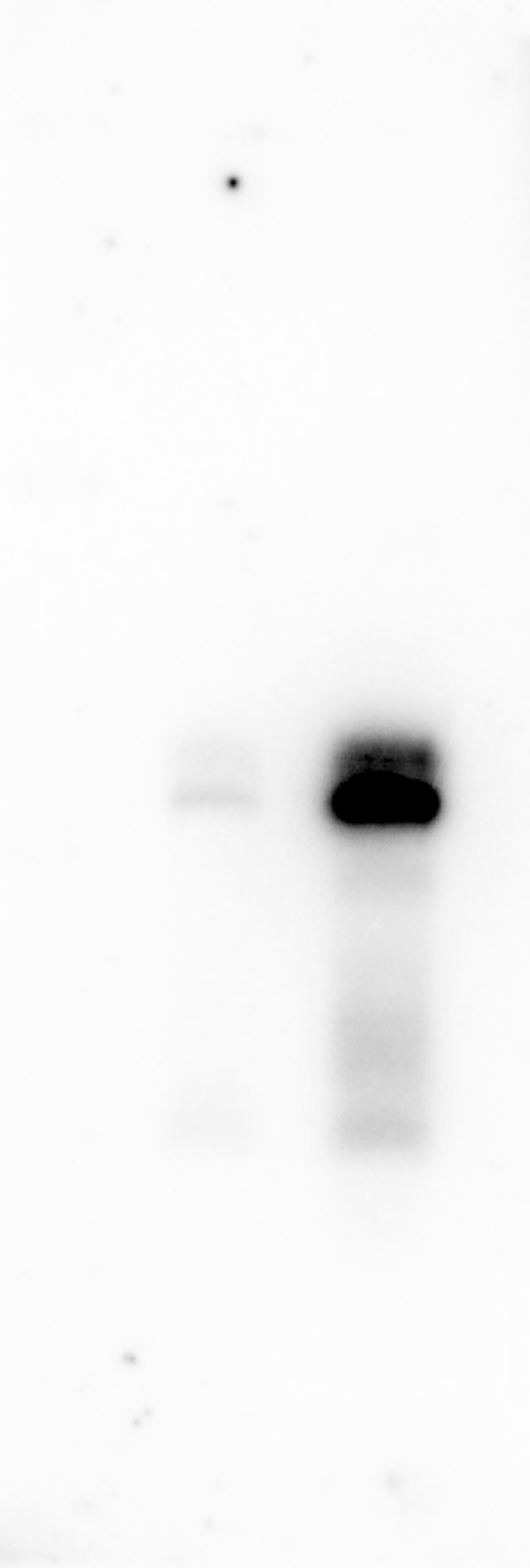

Supplement: Figure 3—source data 1. — Uncropped blots accompanied by images indicating the areas shown in Figure 3B with a red rectangle. In addition, raw scan images are provided. If the scan contains multiple blots, the position of the blot of interest is indicated in the file name. [file elife-95407-fig3-data1.zip › Fig 3-source data 1/Figure 3B-source data RNA17 blot.jpg]

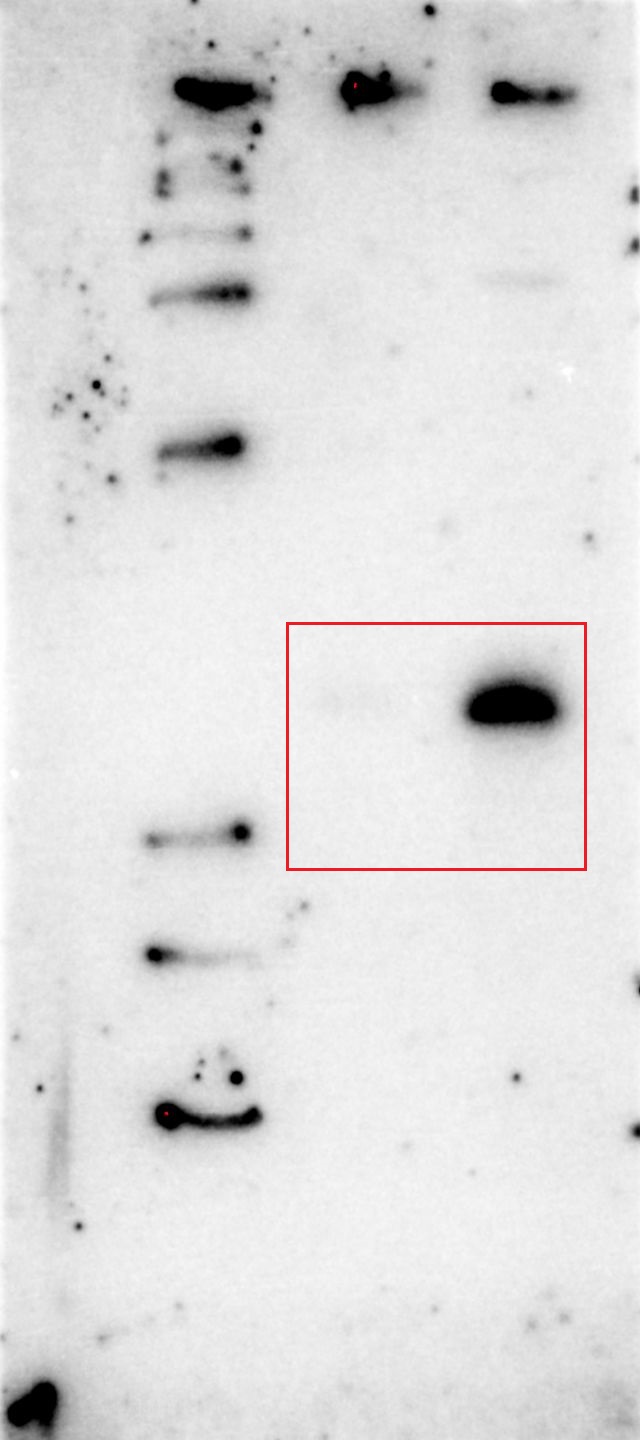

Supplement: Figure 3—source data 1. — Uncropped blots accompanied by images indicating the areas shown in Figure 3B with a red rectangle. In addition, raw scan images are provided. If the scan contains multiple blots, the position of the blot of interest is indicated in the file name. [file elife-95407-fig3-data1.zip › Fig 3-source data 1/Figure 3B-source data RNA29 blot labeled.jpg]

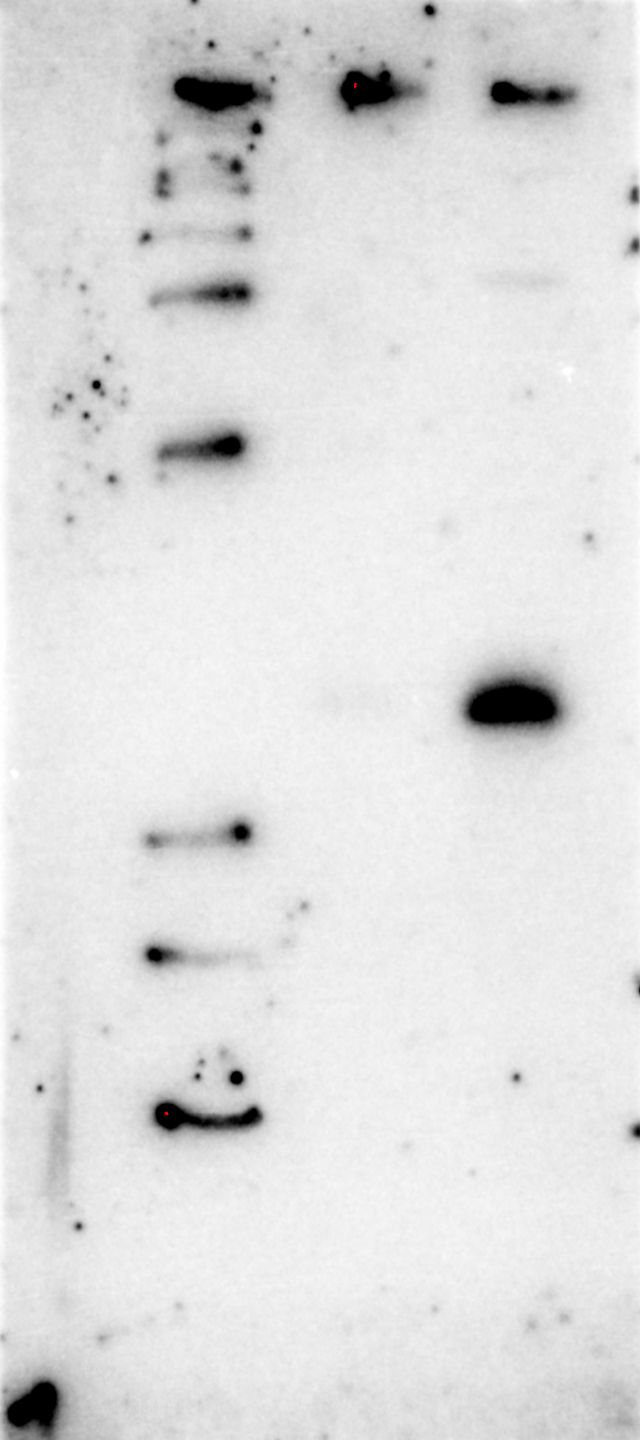

Supplement: Figure 3—source data 1. — Uncropped blots accompanied by images indicating the areas shown in Figure 3B with a red rectangle. In addition, raw scan images are provided. If the scan contains multiple blots, the position of the blot of interest is indicated in the file name. [file elife-95407-fig3-data1.zip › Fig 3-source data 1/Figure 3B-source data RNA29 blot.jpg]

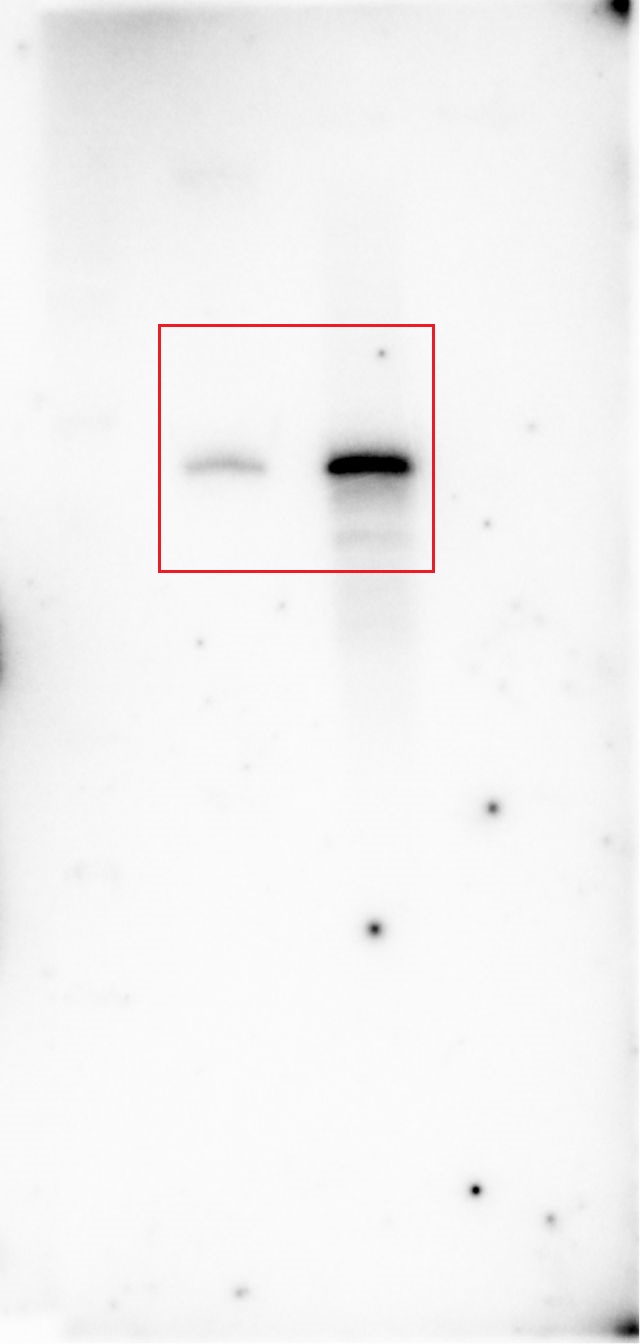

Supplement: Figure 3—source data 1. — Uncropped blots accompanied by images indicating the areas shown in Figure 3B with a red rectangle. In addition, raw scan images are provided. If the scan contains multiple blots, the position of the blot of interest is indicated in the file name. [file elife-95407-fig3-data1.zip › Fig 3-source data 1/Figure 3B-source data RNA5 blot labeled.jpg]

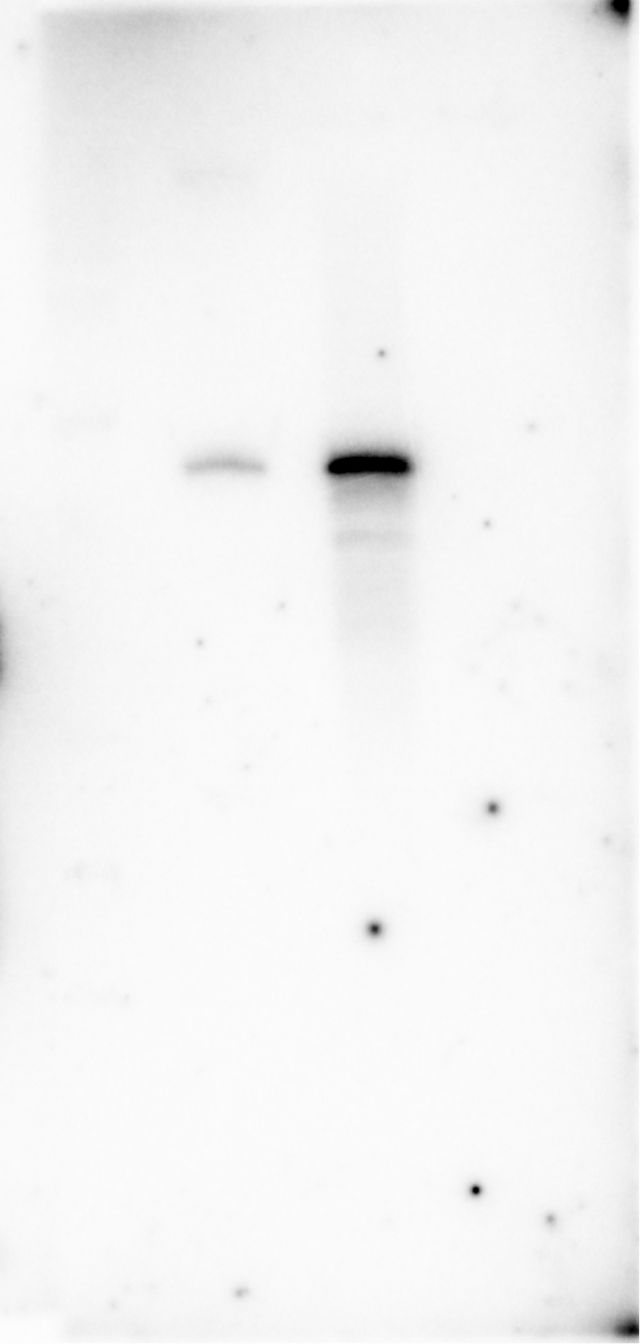

Supplement: Figure 3—source data 1. — Uncropped blots accompanied by images indicating the areas shown in Figure 3B with a red rectangle. In addition, raw scan images are provided. If the scan contains multiple blots, the position of the blot of interest is indicated in the file name. [file elife-95407-fig3-data1.zip › Fig 3-source data 1/Figure 3B-source data RNA5 blot.jpg]

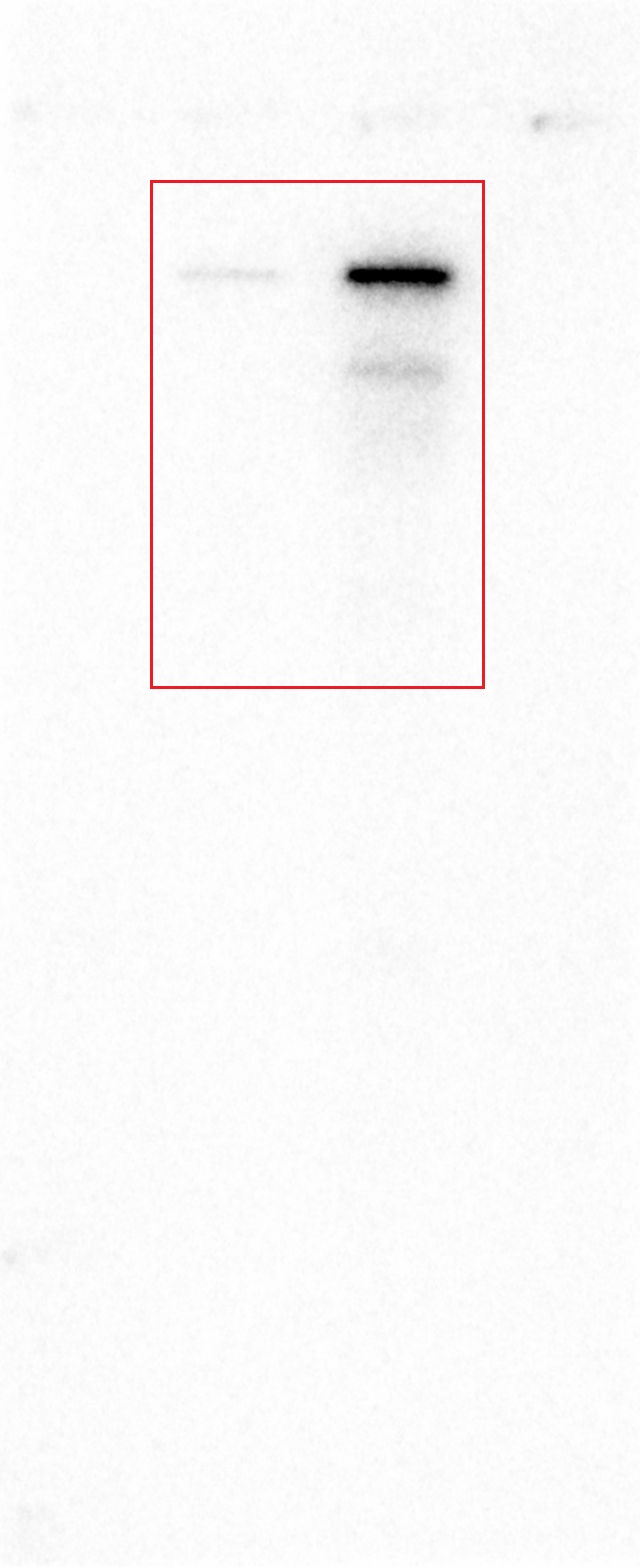

Supplement: Figure 3—figure supplement 1—source data 1. — Uncropped blots accompanied by images indicating the areas shown in Figure 3—figure supplement 1A–B with a red rectangle. In addition, raw scan images are provided. If the scan contains multiple blots, the position of the blot of interest is indicated in the file name. [file elife-95407-fig3-figsupp1-data1.zip › Fig 3-figure supplement 1-source data/Figure 3-figure supplement 1A-source data blot probe d labeled.jpg]

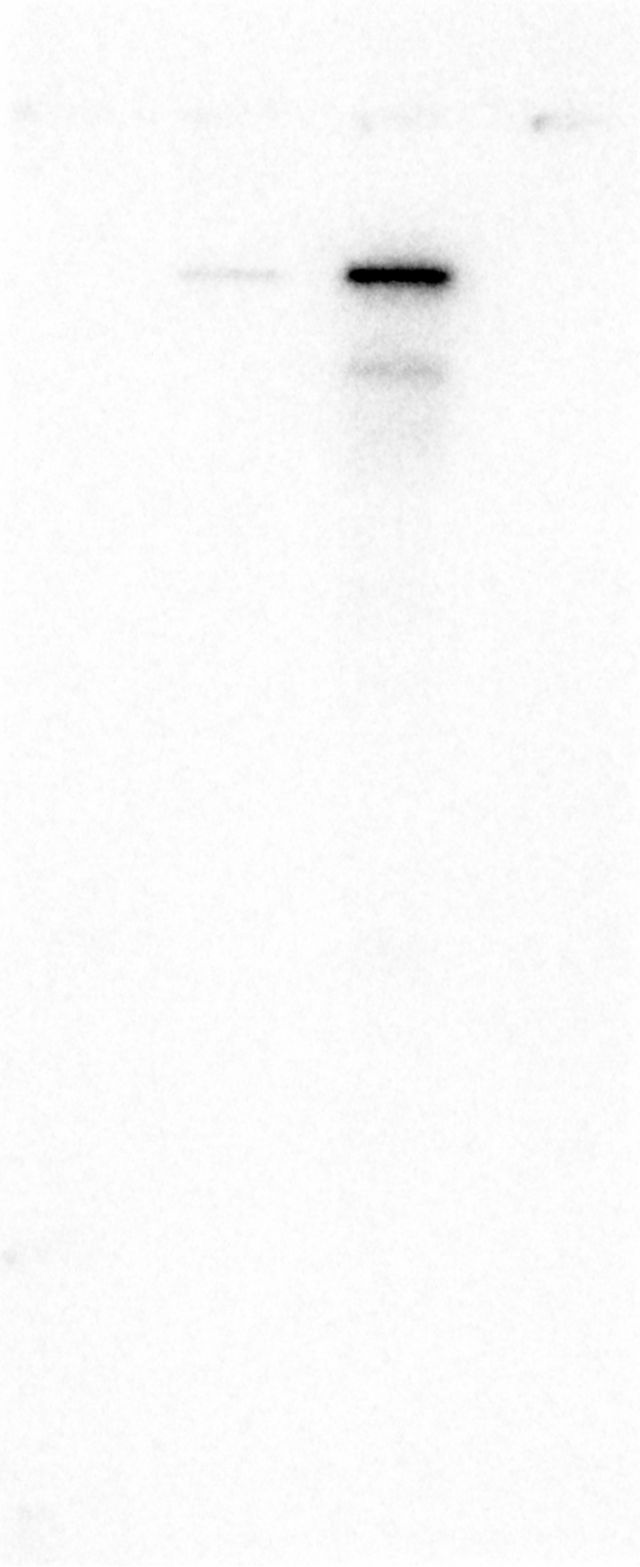

Supplement: Figure 3—figure supplement 1—source data 1. — Uncropped blots accompanied by images indicating the areas shown in Figure 3—figure supplement 1A–B with a red rectangle. In addition, raw scan images are provided. If the scan contains multiple blots, the position of the blot of interest is indicated in the file name. [file elife-95407-fig3-figsupp1-data1.zip › Fig 3-figure supplement 1-source data/Figure 3-figure supplement 1A-source data blot probe d.jpg]

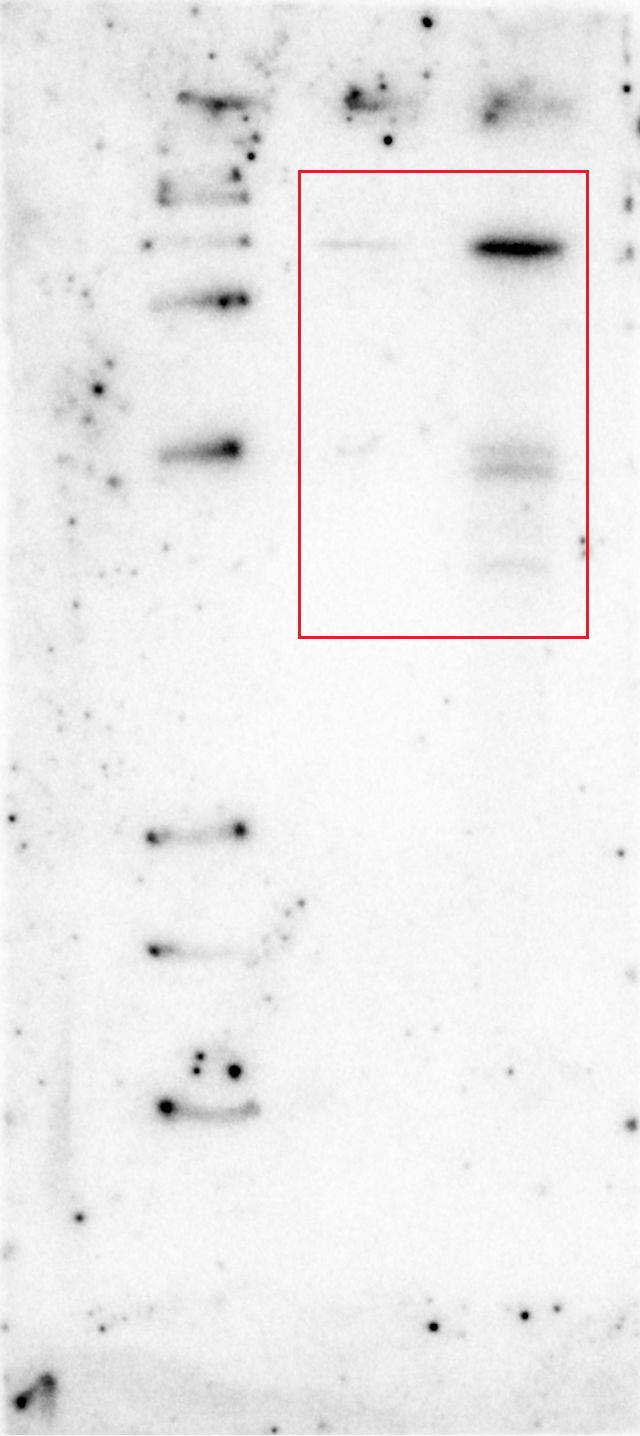

Supplement: Figure 3—figure supplement 1—source data 1. — Uncropped blots accompanied by images indicating the areas shown in Figure 3—figure supplement 1A–B with a red rectangle. In addition, raw scan images are provided. If the scan contains multiple blots, the position of the blot of interest is indicated in the file name. [file elife-95407-fig3-figsupp1-data1.zip › Fig 3-figure supplement 1-source data/Figure 3-figure supplement 1A-source data blot probe e labeled.jpg]

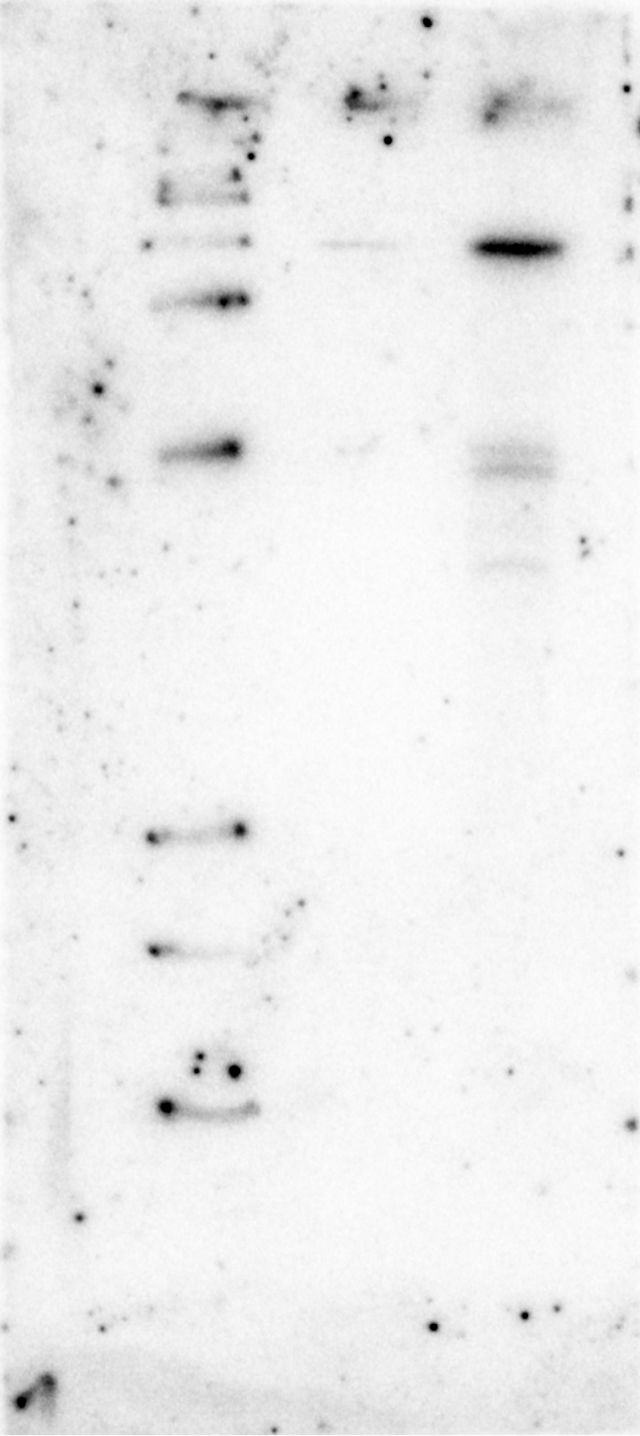

Supplement: Figure 3—figure supplement 1—source data 1. — Uncropped blots accompanied by images indicating the areas shown in Figure 3—figure supplement 1A–B with a red rectangle. In addition, raw scan images are provided. If the scan contains multiple blots, the position of the blot of interest is indicated in the file name. [file elife-95407-fig3-figsupp1-data1.zip › Fig 3-figure supplement 1-source data/Figure 3-figure supplement 1A-source data blot probe e.jpg]

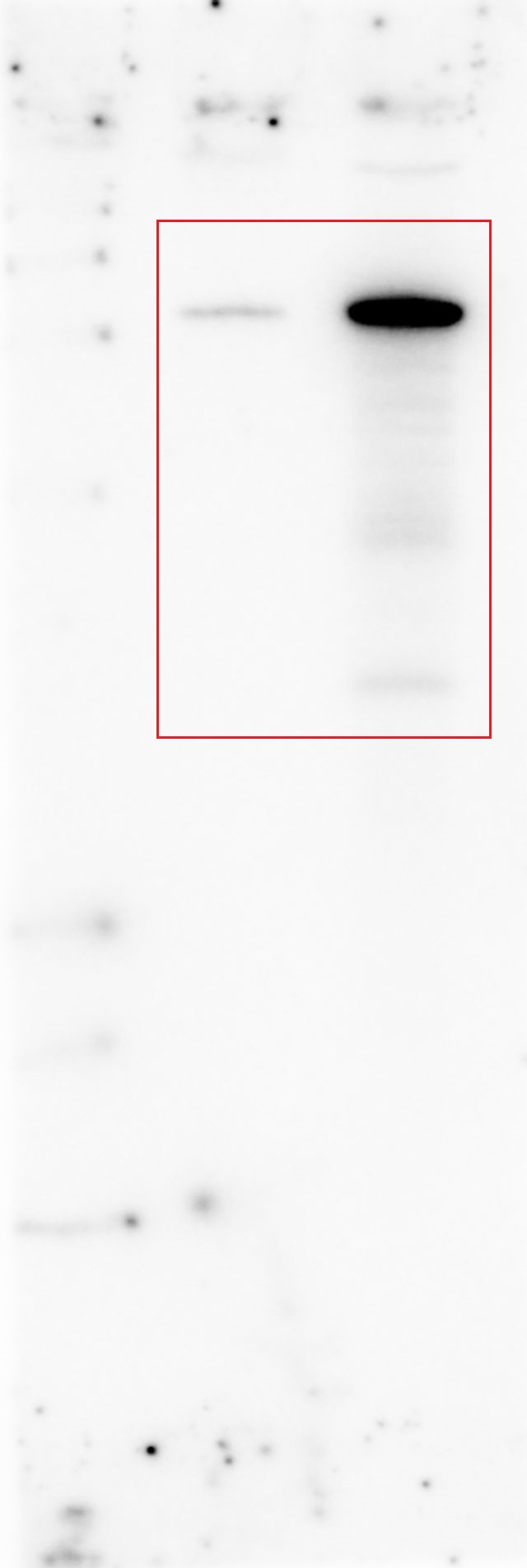

Supplement: Figure 3—figure supplement 1—source data 1. — Uncropped blots accompanied by images indicating the areas shown in Figure 3—figure supplement 1A–B with a red rectangle. In addition, raw scan images are provided. If the scan contains multiple blots, the position of the blot of interest is indicated in the file name. [file elife-95407-fig3-figsupp1-data1.zip › Fig 3-figure supplement 1-source data/Figure 3-figure supplement 1B-source data blot probe f labeled.jpg]

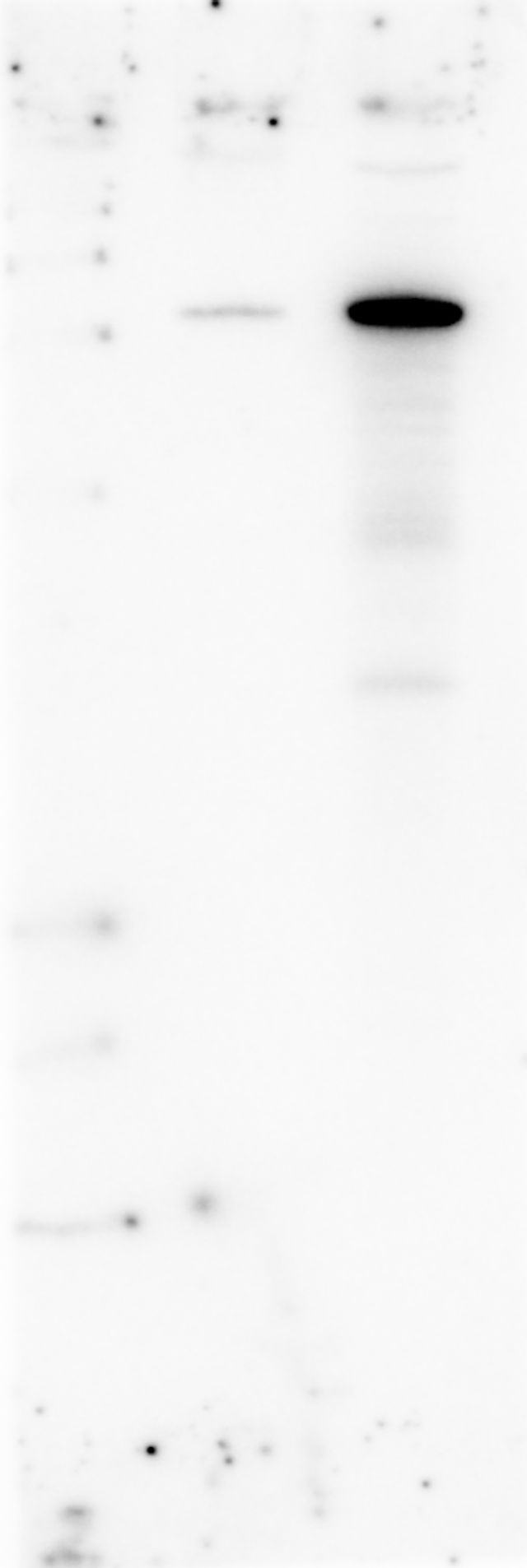

Supplement: Figure 3—figure supplement 1—source data 1. — Uncropped blots accompanied by images indicating the areas shown in Figure 3—figure supplement 1A–B with a red rectangle. In addition, raw scan images are provided. If the scan contains multiple blots, the position of the blot of interest is indicated in the file name. [file elife-95407-fig3-figsupp1-data1.zip › Fig 3-figure supplement 1-source data/Figure 3-figure supplement 1B-source data blot probe f.jpg]

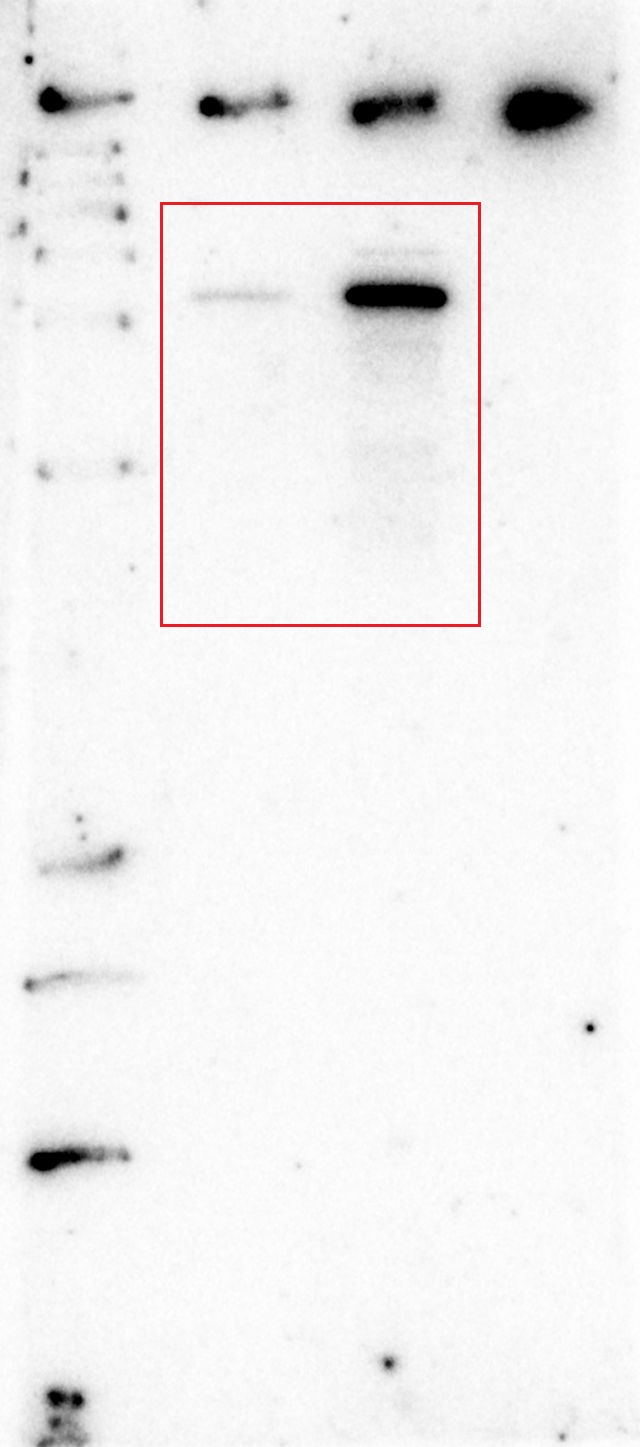

Supplement: Figure 3—figure supplement 1—source data 1. — Uncropped blots accompanied by images indicating the areas shown in Figure 3—figure supplement 1A–B with a red rectangle. In addition, raw scan images are provided. If the scan contains multiple blots, the position of the blot of interest is indicated in the file name. [file elife-95407-fig3-figsupp1-data1.zip › Fig 3-figure supplement 1-source data/Figure 3-figure supplement 1B-source data blot probe g labeled.jpg]

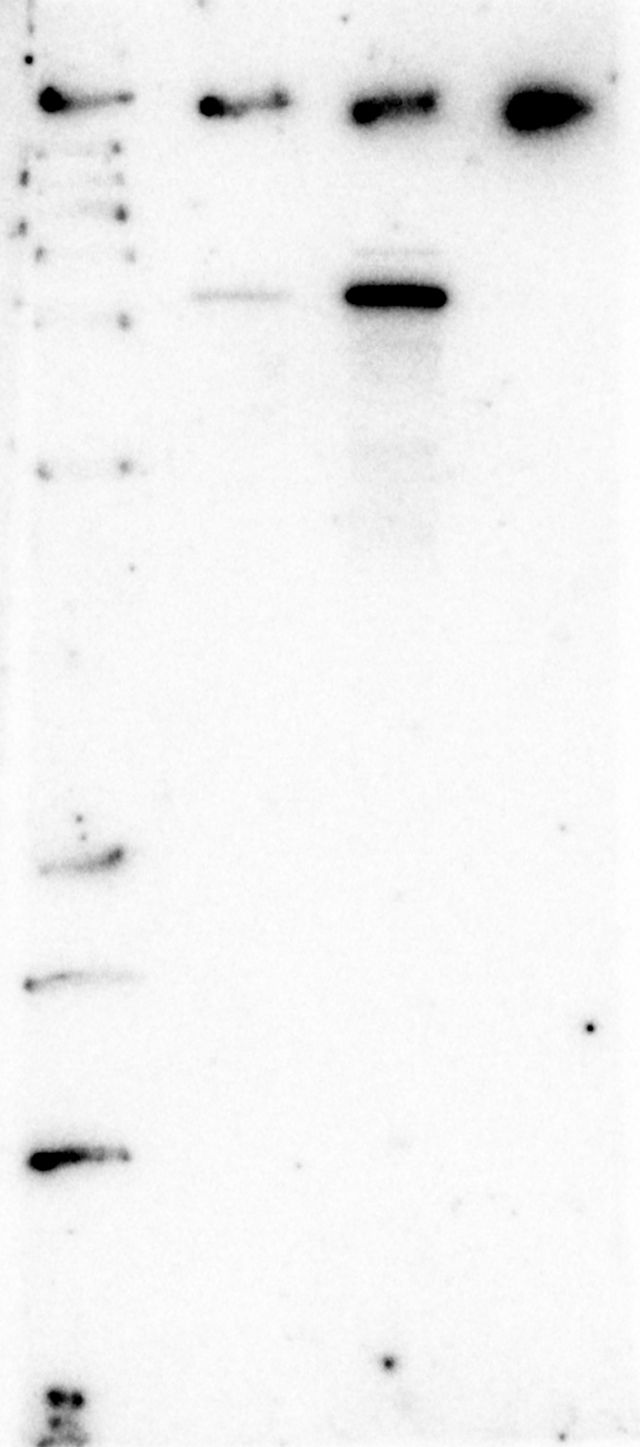

Supplement: Figure 3—figure supplement 1—source data 1. — Uncropped blots accompanied by images indicating the areas shown in Figure 3—figure supplement 1A–B with a red rectangle. In addition, raw scan images are provided. If the scan contains multiple blots, the position of the blot of interest is indicated in the file name. [file elife-95407-fig3-figsupp1-data1.zip › Fig 3-figure supplement 1-source data/Figure 3-figure supplement 1B-source data blot probe g.jpg]

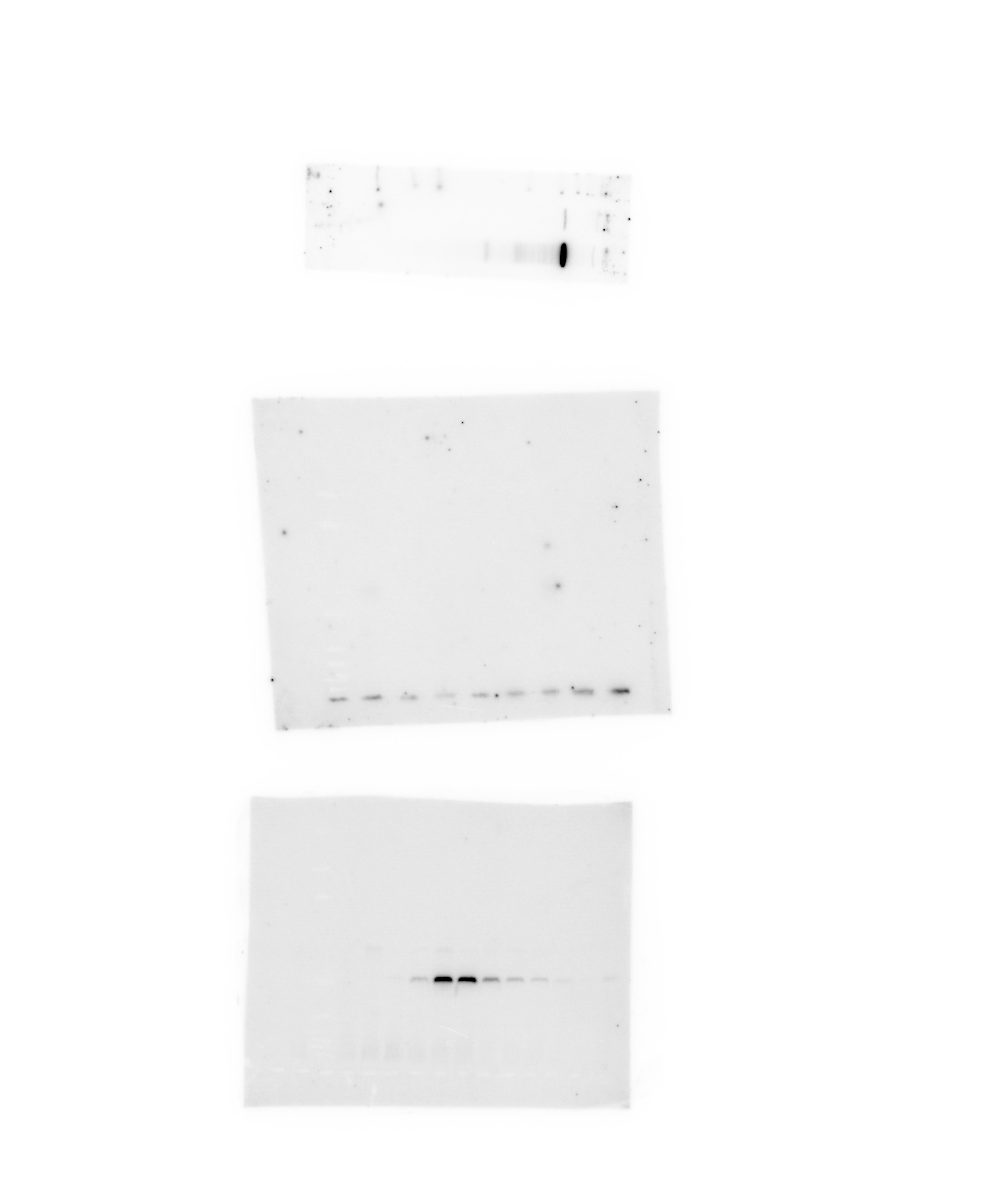

Supplement: Figure 3—figure supplement 1—source data 1. — Uncropped blots accompanied by images indicating the areas shown in Figure 3—figure supplement 1A–B with a red rectangle. In addition, raw scan images are provided. If the scan contains multiple blots, the position of the blot of interest is indicated in the file name. [file elife-95407-fig3-figsupp1-data1.zip › Fig 3-figure supplement 1-source data/Figure 3-figure supplement 1B-source data probe f raw scan (top).tif]

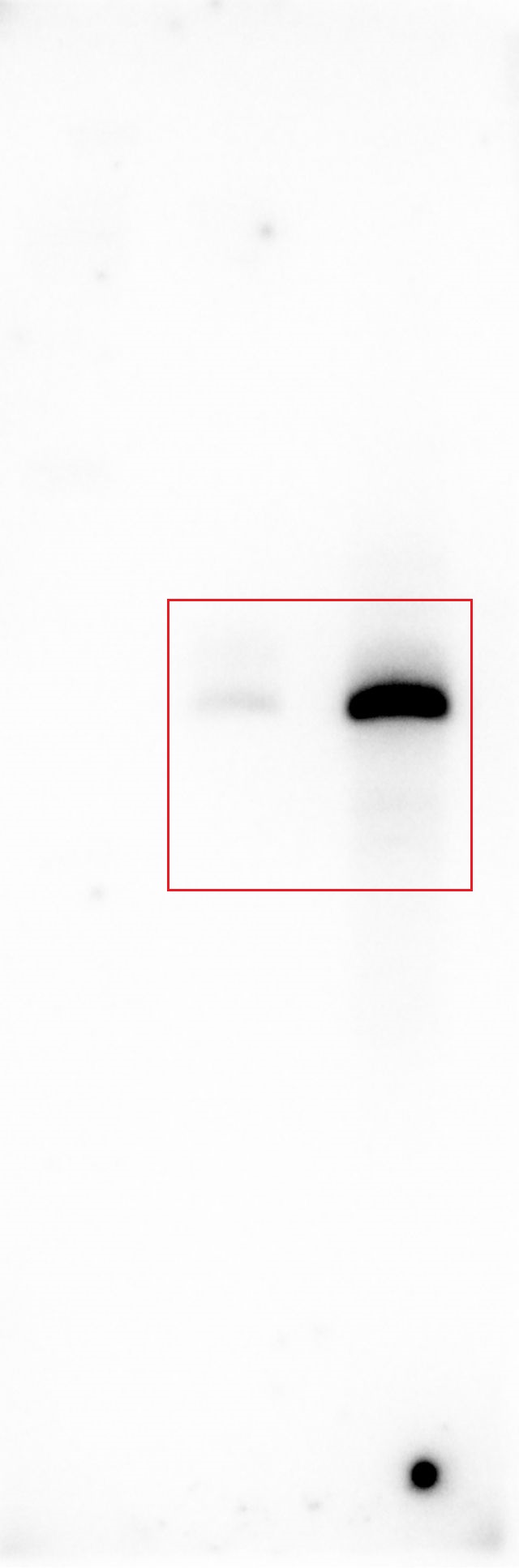

Supplement: Figure 3—figure supplement 2—source data 1. — Uncropped blots accompanied by images indicating the areas shown in Figure 3—figure supplement 2B with a red rectangle. In addition, raw scan images are provided. If the scan contains multiple blots, the position of the blot of interest is indicated in the file name. [file elife-95407-fig3-figsupp2-data1.zip › Fig 3-figure supplement 2-source data/Figure 3-figure supplement 2B-source data RNA6 blot labeled.jpg]

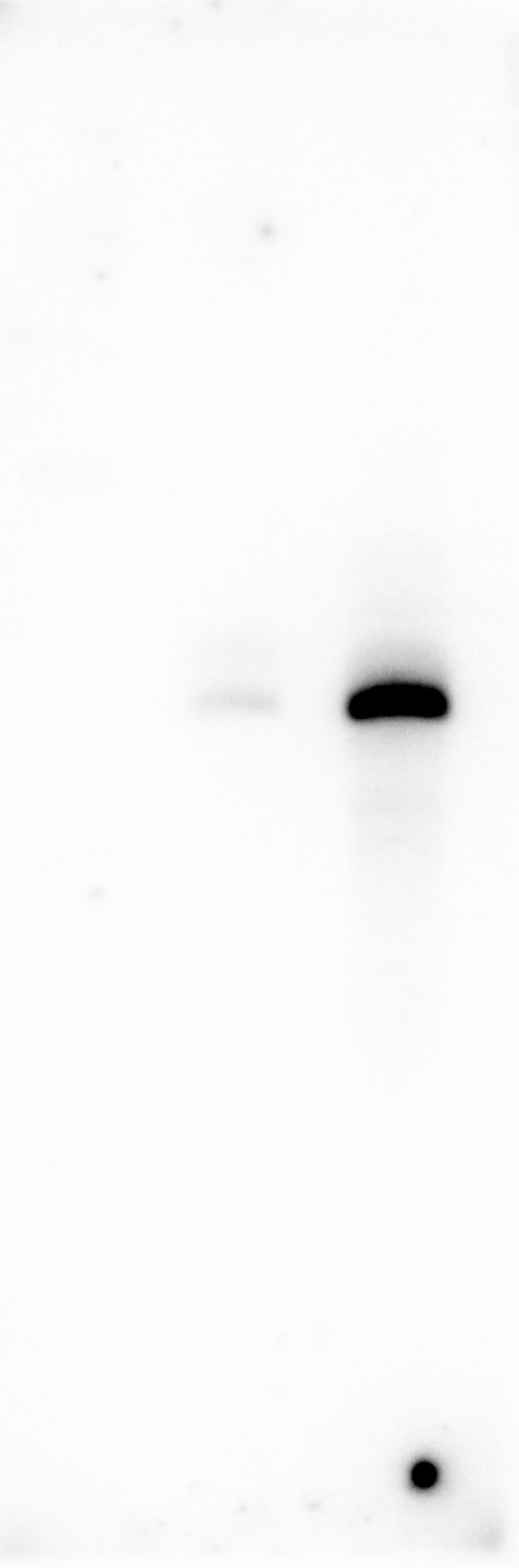

Supplement: Figure 3—figure supplement 2—source data 1. — Uncropped blots accompanied by images indicating the areas shown in Figure 3—figure supplement 2B with a red rectangle. In addition, raw scan images are provided. If the scan contains multiple blots, the position of the blot of interest is indicated in the file name. [file elife-95407-fig3-figsupp2-data1.zip › Fig 3-figure supplement 2-source data/Figure 3-figure supplement 2B-source data RNA6 blot.jpg]

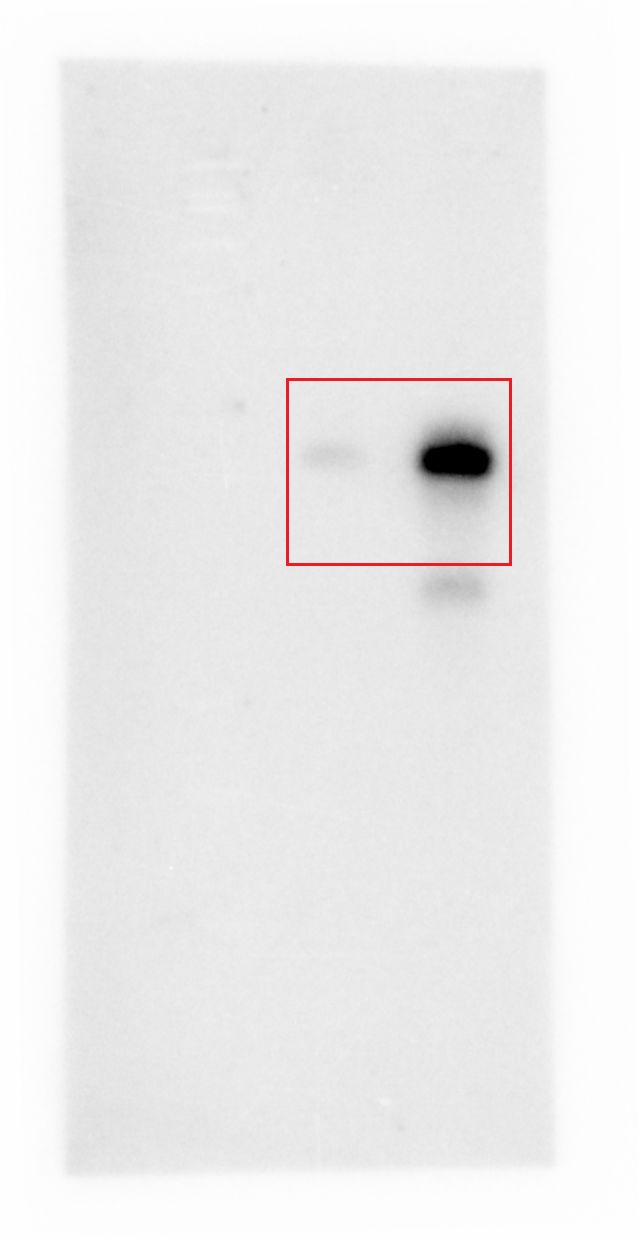

Supplement: Figure 3—figure supplement 2—source data 1. — Uncropped blots accompanied by images indicating the areas shown in Figure 3—figure supplement 2B with a red rectangle. In addition, raw scan images are provided. If the scan contains multiple blots, the position of the blot of interest is indicated in the file name. [file elife-95407-fig3-figsupp2-data1.zip › Fig 3-figure supplement 2-source data/Figure 3-figure supplement 2B-source data RNA7 blot labeled.jpg]

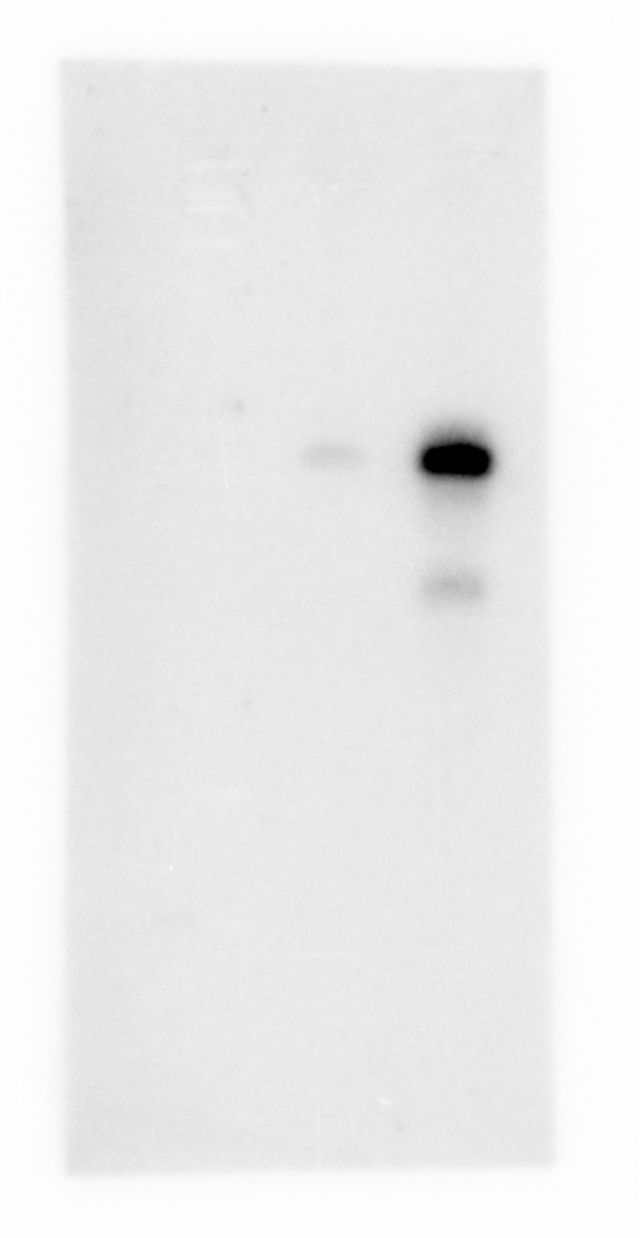

Supplement: Figure 3—figure supplement 2—source data 1. — Uncropped blots accompanied by images indicating the areas shown in Figure 3—figure supplement 2B with a red rectangle. In addition, raw scan images are provided. If the scan contains multiple blots, the position of the blot of interest is indicated in the file name. [file elife-95407-fig3-figsupp2-data1.zip › Fig 3-figure supplement 2-source data/Figure 3-figure supplement 2B-source data RNA7 blot.jpg]

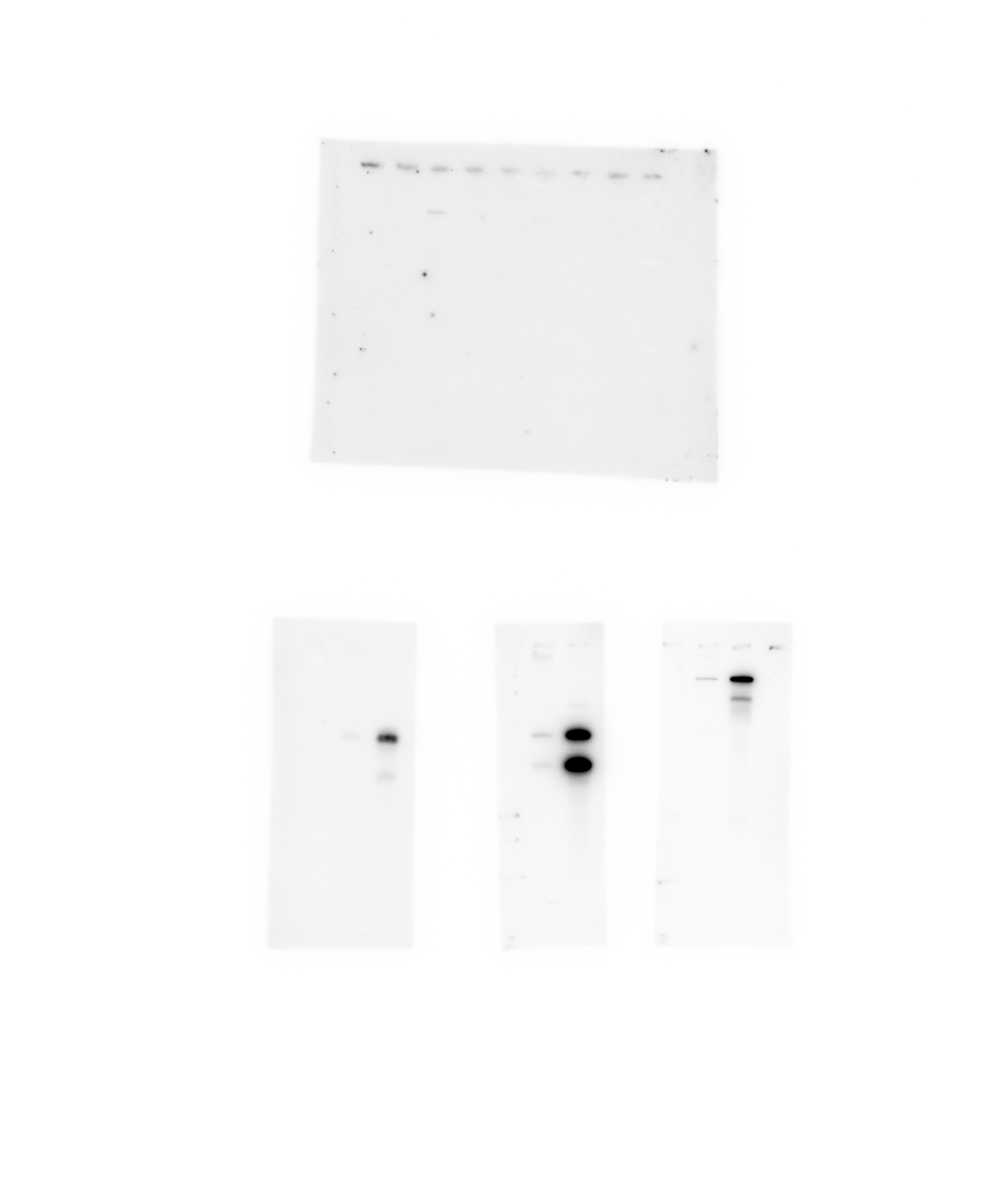

Supplement: Figure 3—figure supplement 2—source data 1. — Uncropped blots accompanied by images indicating the areas shown in Figure 3—figure supplement 2B with a red rectangle. In addition, raw scan images are provided. If the scan contains multiple blots, the position of the blot of interest is indicated in the file name. [file elife-95407-fig3-figsupp2-data1.zip › Fig 3-figure supplement 2-source data/Figure 3-figure supplement 2B-source data RNA7 scan (bottom left).tif]

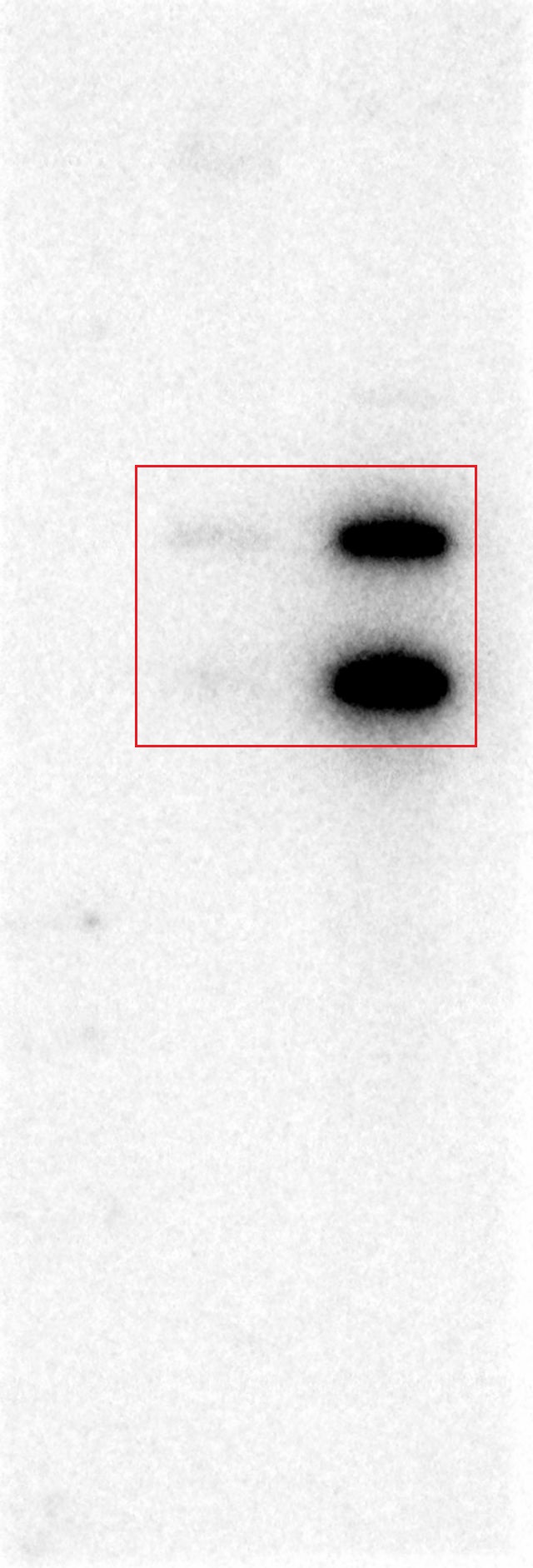

Supplement: Figure 4—source data 1. — Uncropped blot accompanied by an image indicating the areas shown in Figure 4B with a red rectangle. In addition, the raw scan image is provided with the position of the blot of interest being indicated in the file name. [file elife-95407-fig4-data1.zip › Fig 4-source data 1/Figure 4B-source data RNA1-2 blot labeled.jpg]

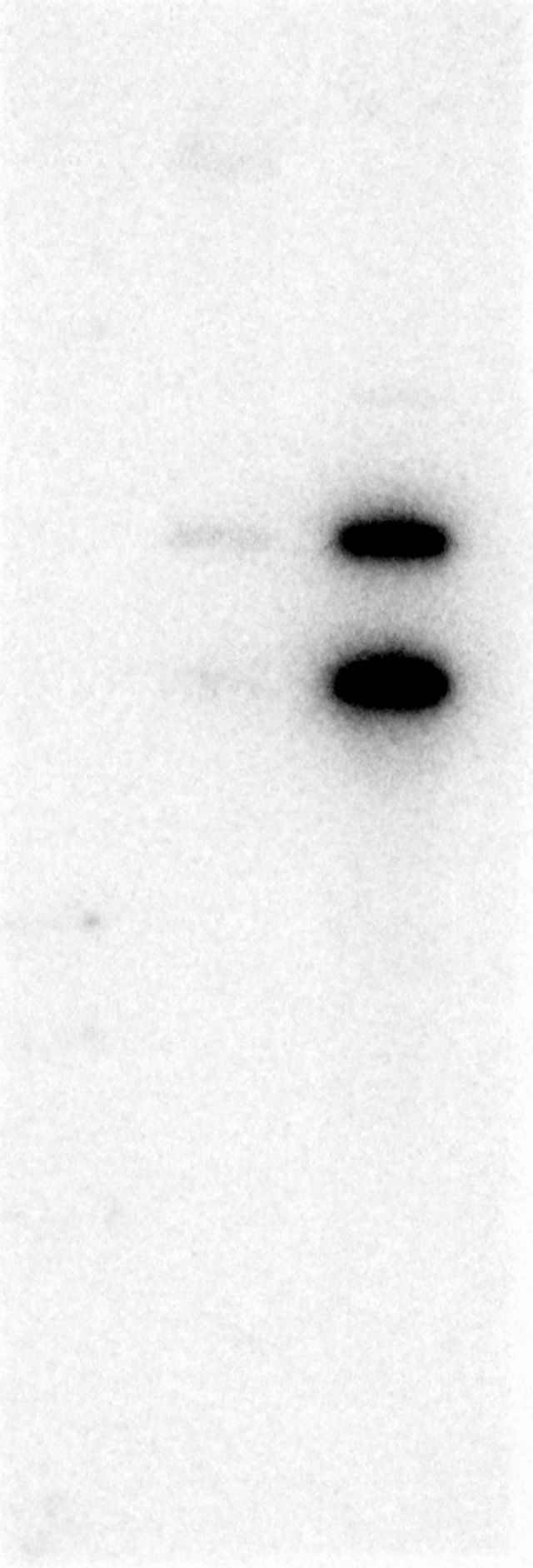

Supplement: Figure 4—source data 1. — Uncropped blot accompanied by an image indicating the areas shown in Figure 4B with a red rectangle. In addition, the raw scan image is provided with the position of the blot of interest being indicated in the file name. [file elife-95407-fig4-data1.zip › Fig 4-source data 1/Figure 4B-source data RNA1-2 blot.jpg]

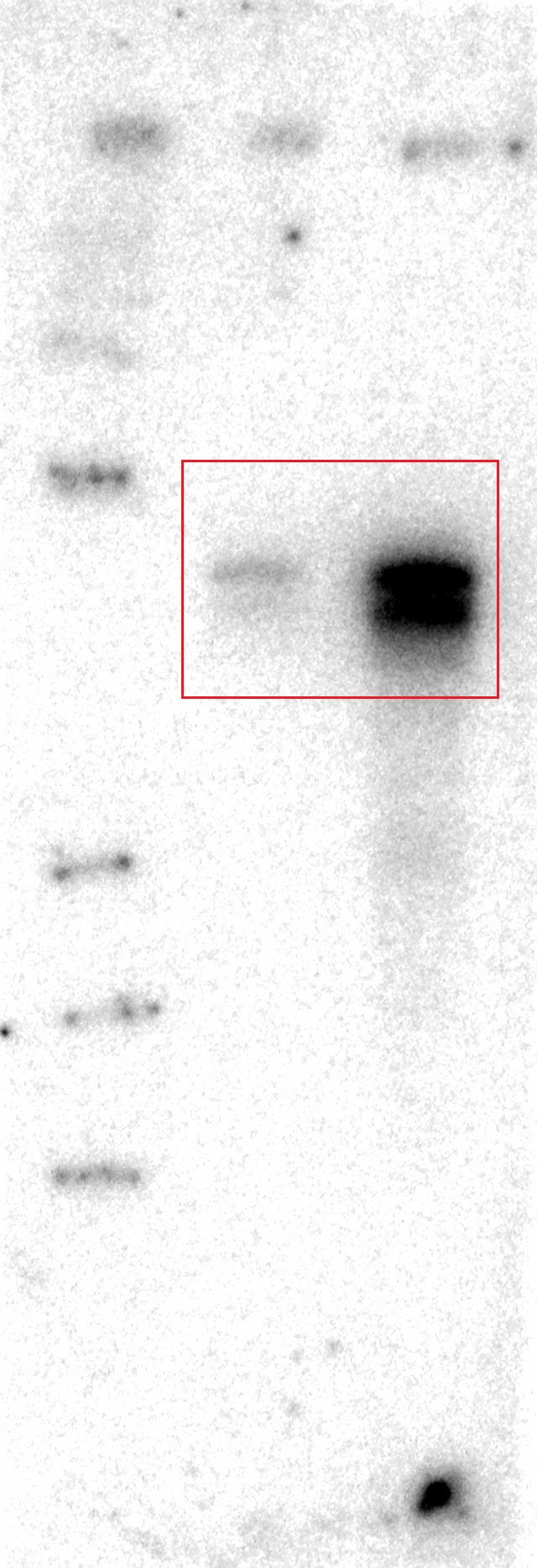

Supplement: Figure 5—source data 1. — Uncropped blots and gels accompanied by images indicating the areas shown in Figure 5C and D and -G with a red rectangle. In addition, raw scan images are provided. [file elife-95407-fig5-data1.zip › Fig 5-source data 1/Fig 5C-source data RNA34 blot labeled.jpg]

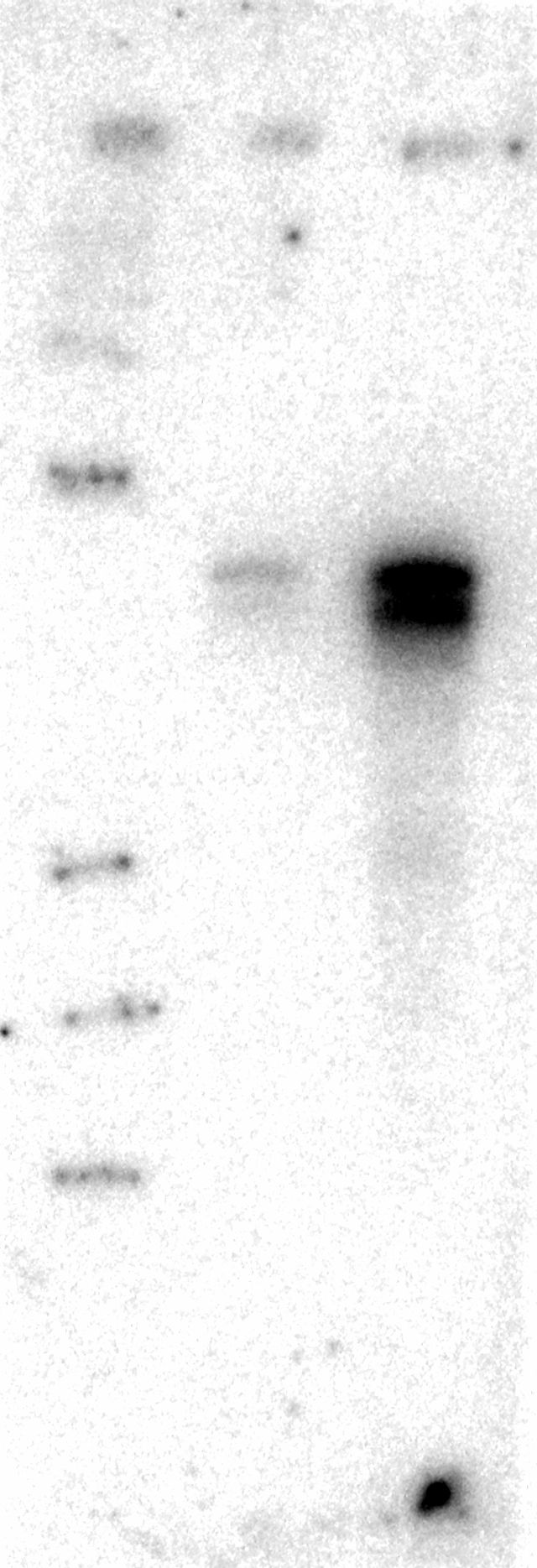

Supplement: Figure 5—source data 1. — Uncropped blots and gels accompanied by images indicating the areas shown in Figure 5C and D and -G with a red rectangle. In addition, raw scan images are provided. [file elife-95407-fig5-data1.zip › Fig 5-source data 1/Fig 5C-source data RNA34 blot.jpg]

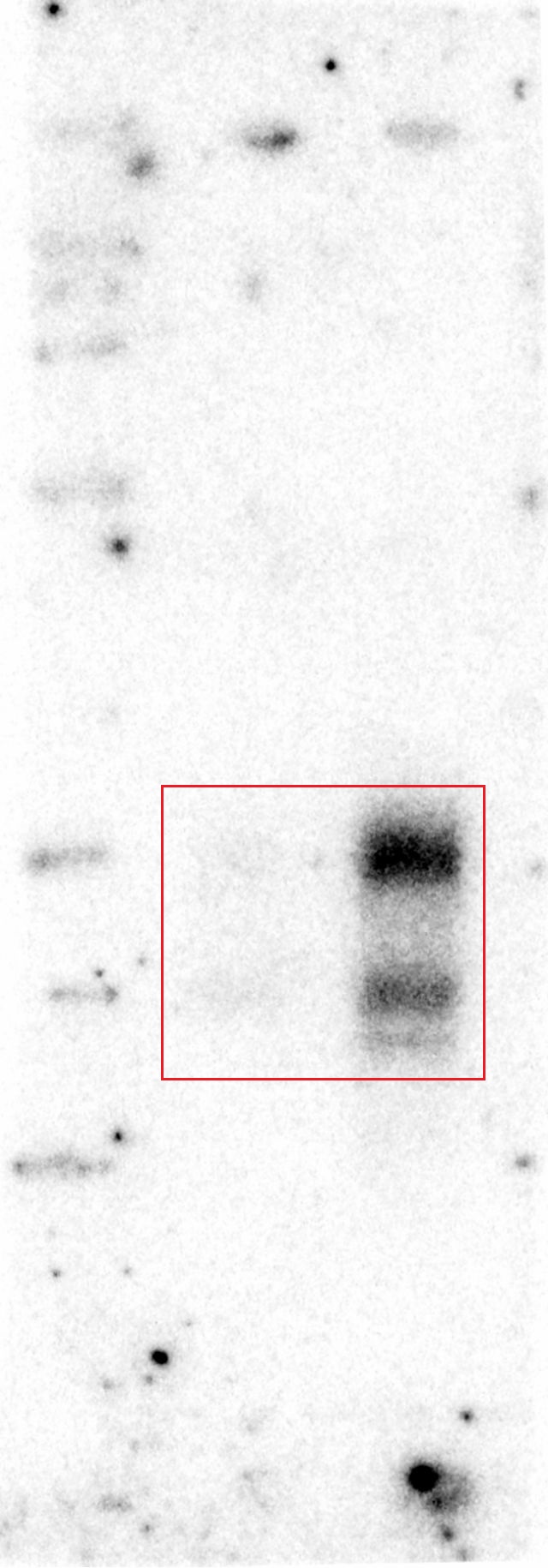

Supplement: Figure 5—source data 1. — Uncropped blots and gels accompanied by images indicating the areas shown in Figure 5C and D and -G with a red rectangle. In addition, raw scan images are provided. [file elife-95407-fig5-data1.zip › Fig 5-source data 1/Fig 5D-source data RNA19 blot labeled.jpg]

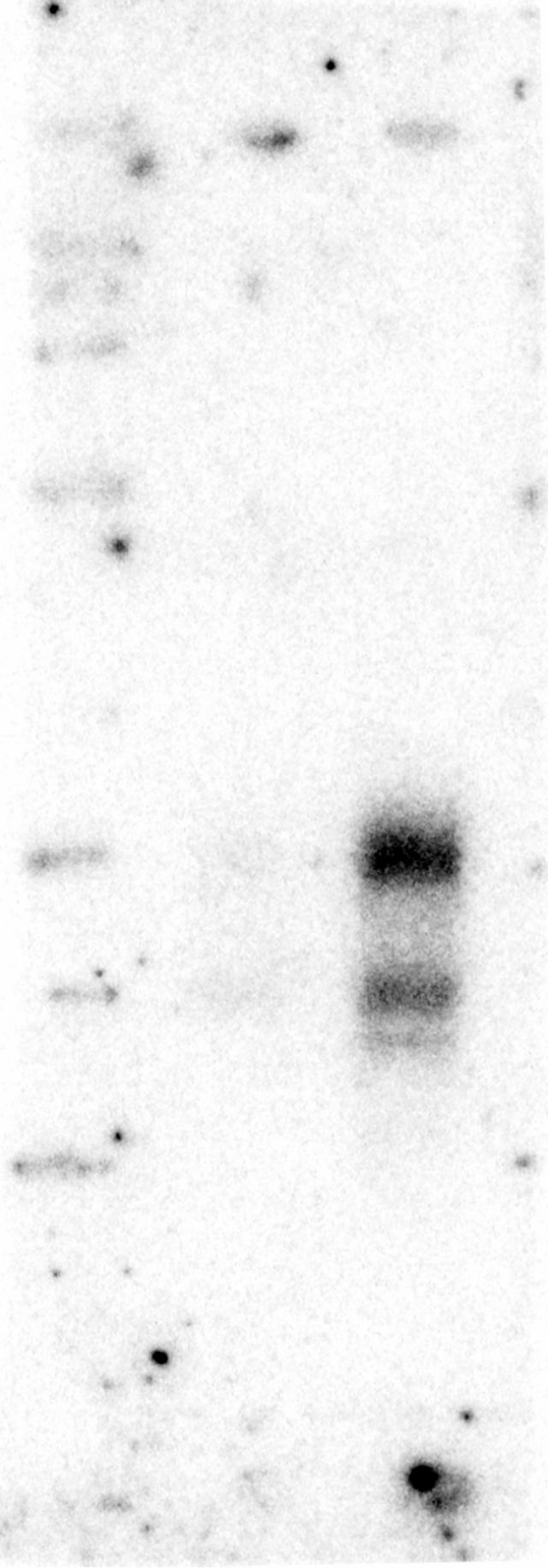

Supplement: Figure 5—source data 1. — Uncropped blots and gels accompanied by images indicating the areas shown in Figure 5C and D and -G with a red rectangle. In addition, raw scan images are provided. [file elife-95407-fig5-data1.zip › Fig 5-source data 1/Fig 5D-source data RNA19 blot.jpg]

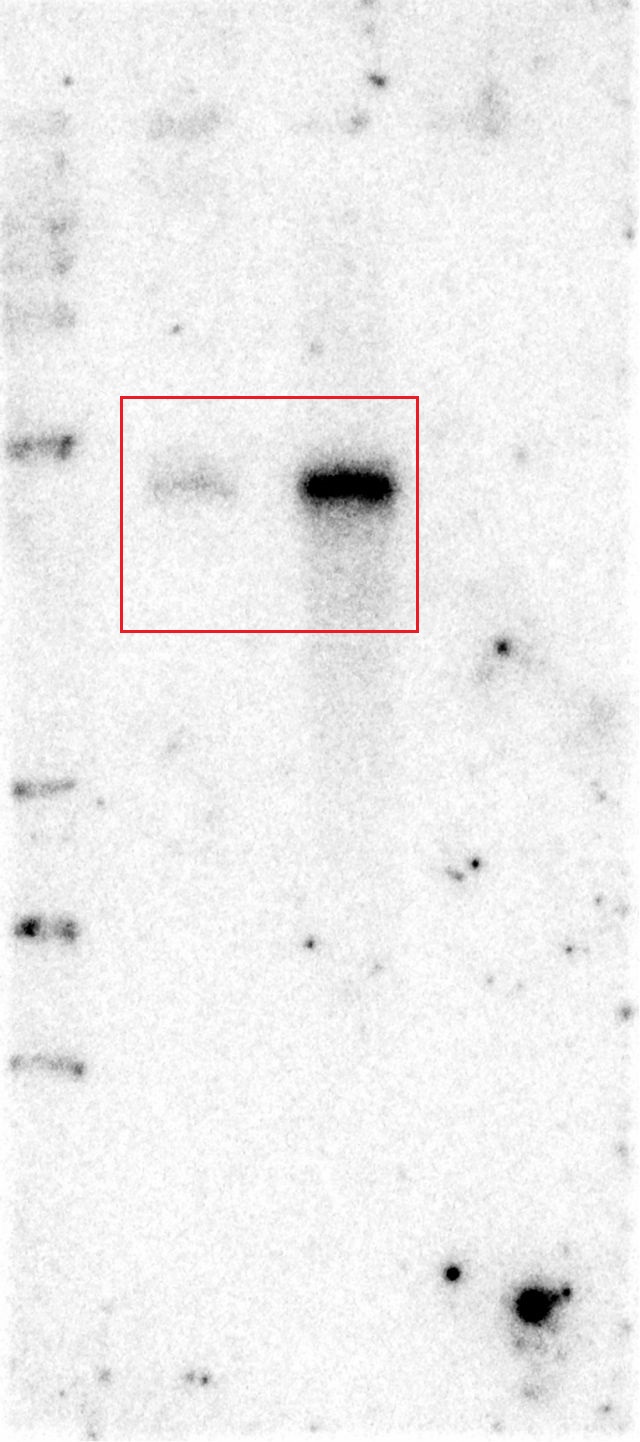

Supplement: Figure 5—source data 1. — Uncropped blots and gels accompanied by images indicating the areas shown in Figure 5C and D and -G with a red rectangle. In addition, raw scan images are provided. [file elife-95407-fig5-data1.zip › Fig 5-source data 1/Fig 5D-source data RNA3 blot labeled.jpg]

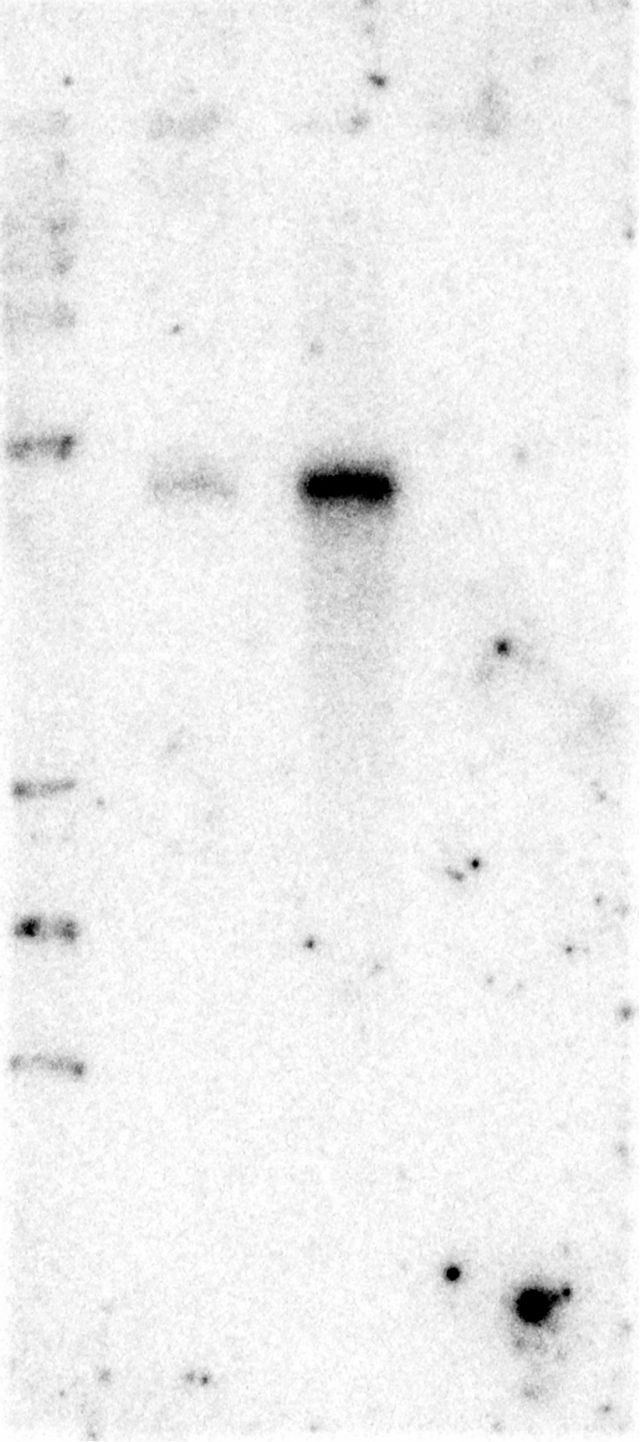

Supplement: Figure 5—source data 1. — Uncropped blots and gels accompanied by images indicating the areas shown in Figure 5C and D and -G with a red rectangle. In addition, raw scan images are provided. [file elife-95407-fig5-data1.zip › Fig 5-source data 1/Fig 5D-source data RNA3 blot.jpg]

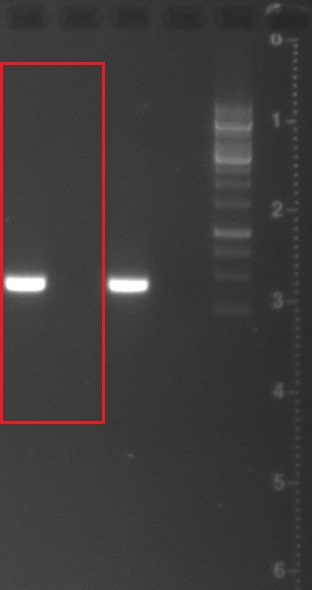

Supplement: Figure 5—source data 1. — Uncropped blots and gels accompanied by images indicating the areas shown in Figure 5C and D and -G with a red rectangle. In addition, raw scan images are provided. [file elife-95407-fig5-data1.zip › Fig 5-source data 1/Fig 5G-source data cob 5 RACE gel labeled.jpg]

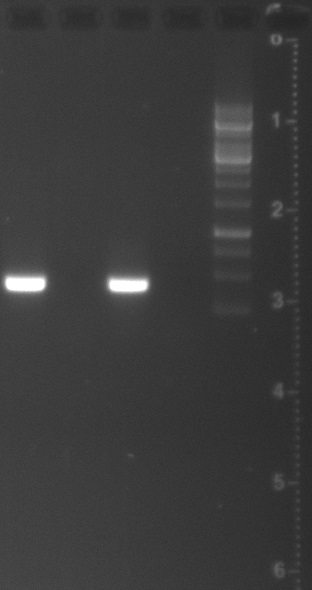

Supplement: Figure 5—source data 1. — Uncropped blots and gels accompanied by images indicating the areas shown in Figure 5C and D and -G with a red rectangle. In addition, raw scan images are provided. [file elife-95407-fig5-data1.zip › Fig 5-source data 1/Fig 5G-source data cob 5 RACE gel.jpg]

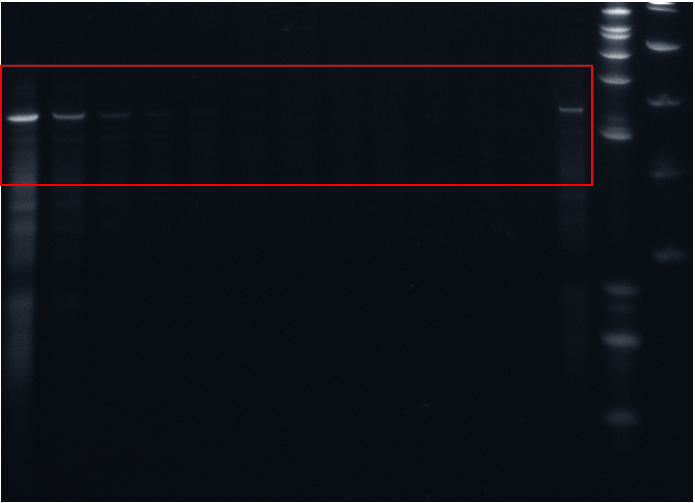

Supplement: Figure 6—source data 1. — Uncropped blots accompanied by images indicating the areas shown in Figure 6A–D with a red rectangle. In addition, raw scan images are provided. If the scan contains multiple blots, the position of the blot of interest is indicated in the file name. [file elife-95407-fig6-data1.zip › Fig 6-source data 1/Figure 6A-source data EDTA ethidiumbr. labeled.jpg]

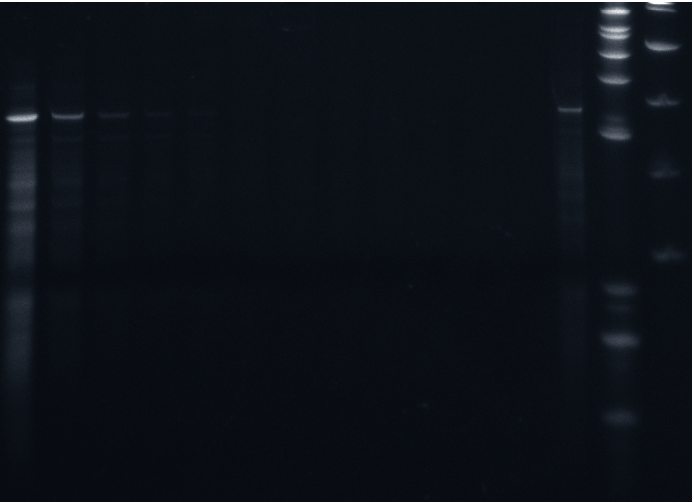

Supplement: Figure 6—source data 1. — Uncropped blots accompanied by images indicating the areas shown in Figure 6A–D with a red rectangle. In addition, raw scan images are provided. If the scan contains multiple blots, the position of the blot of interest is indicated in the file name. [file elife-95407-fig6-data1.zip › Fig 6-source data 1/Figure 6A-source data urea-PAGE EDTA ethidiumbr..jpg]

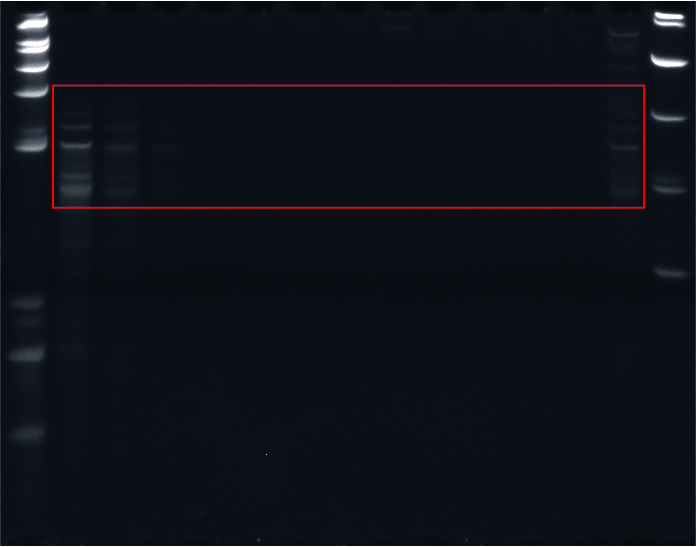

Supplement: Figure 6—source data 1. — Uncropped blots accompanied by images indicating the areas shown in Figure 6A–D with a red rectangle. In addition, raw scan images are provided. If the scan contains multiple blots, the position of the blot of interest is indicated in the file name. [file elife-95407-fig6-data1.zip › Fig 6-source data 1/Figure 6A-source data urea-PAGE ethidiumbr. labeled.jpg]

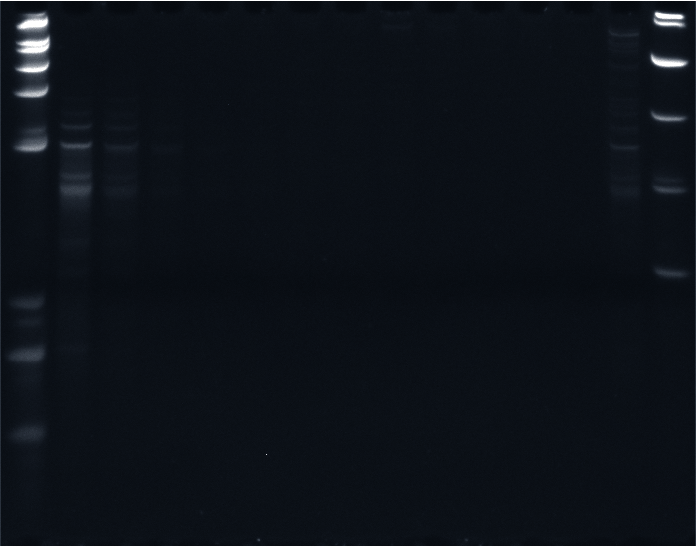

Supplement: Figure 6—source data 1. — Uncropped blots accompanied by images indicating the areas shown in Figure 6A–D with a red rectangle. In addition, raw scan images are provided. If the scan contains multiple blots, the position of the blot of interest is indicated in the file name. [file elife-95407-fig6-data1.zip › Fig 6-source data 1/Figure 6A-source data urea-PAGE Mg ethidiumbr..jpg]

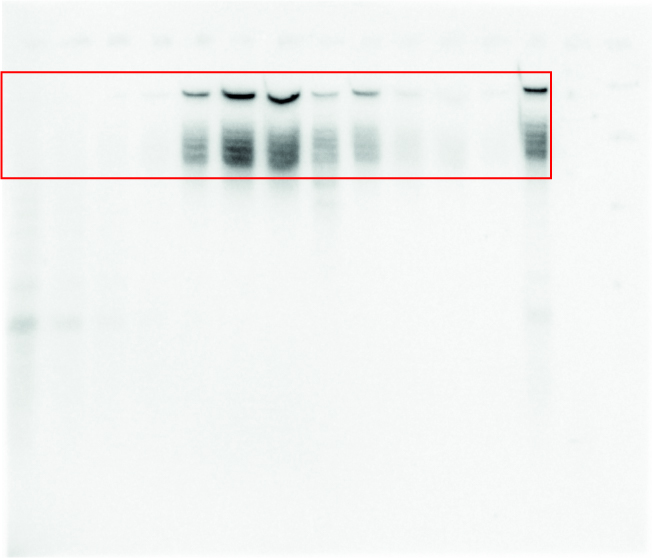

Supplement: Figure 6—source data 1. — Uncropped blots accompanied by images indicating the areas shown in Figure 6A–D with a red rectangle. In addition, raw scan images are provided. If the scan contains multiple blots, the position of the blot of interest is indicated in the file name. [file elife-95407-fig6-data1.zip › Fig 6-source data 1/Figure 6B-source data LSUD-E EDTA blot labeled.jpg]

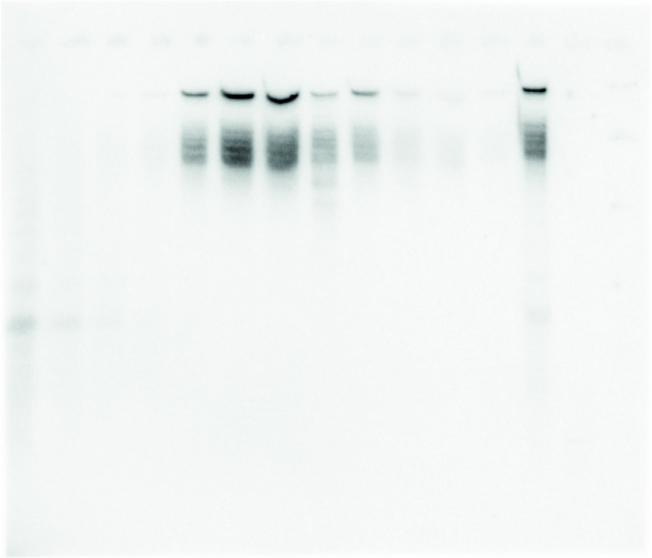

Supplement: Figure 6—source data 1. — Uncropped blots accompanied by images indicating the areas shown in Figure 6A–D with a red rectangle. In addition, raw scan images are provided. If the scan contains multiple blots, the position of the blot of interest is indicated in the file name. [file elife-95407-fig6-data1.zip › Fig 6-source data 1/Figure 6B-source data LSUD-E EDTA blot.jpg]

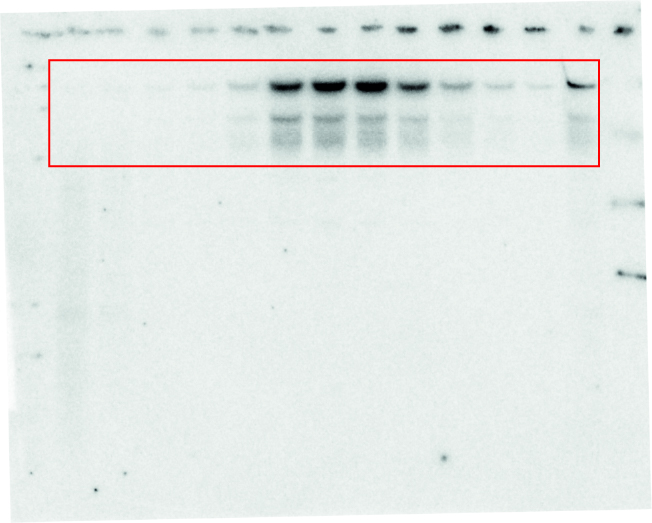

Supplement: Figure 6—source data 1. — Uncropped blots accompanied by images indicating the areas shown in Figure 6A–D with a red rectangle. In addition, raw scan images are provided. If the scan contains multiple blots, the position of the blot of interest is indicated in the file name. [file elife-95407-fig6-data1.zip › Fig 6-source data 1/Figure 6B-source data LSUD-E Mg blot labeled.jpg]

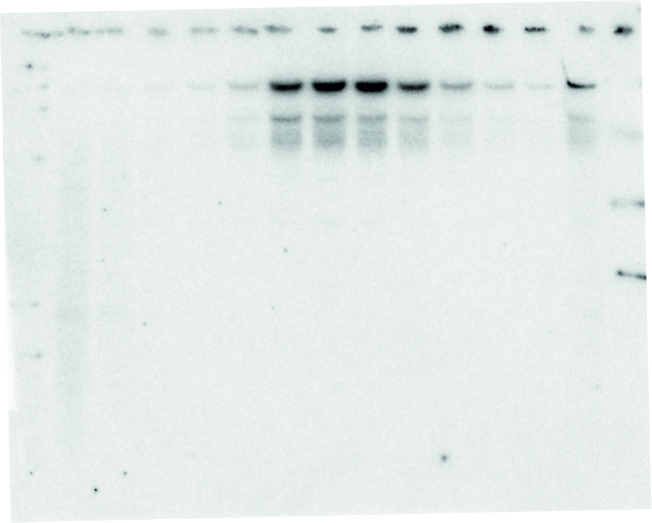

Supplement: Figure 6—source data 1. — Uncropped blots accompanied by images indicating the areas shown in Figure 6A–D with a red rectangle. In addition, raw scan images are provided. If the scan contains multiple blots, the position of the blot of interest is indicated in the file name. [file elife-95407-fig6-data1.zip › Fig 6-source data 1/Figure 6B-source data LSUD-E Mg blot.jpg]

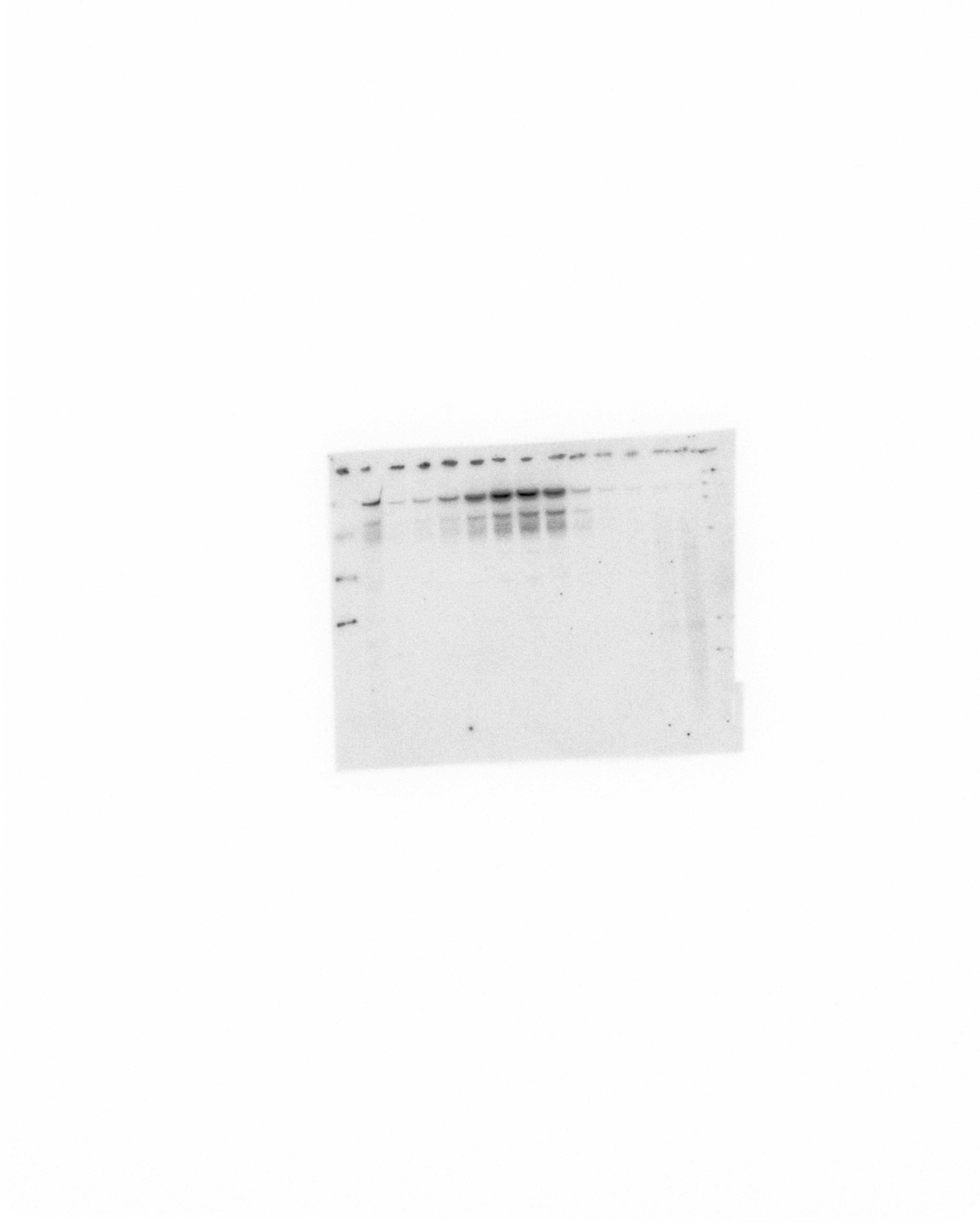

Supplement: Figure 6—source data 1. — Uncropped blots accompanied by images indicating the areas shown in Figure 6A–D with a red rectangle. In addition, raw scan images are provided. If the scan contains multiple blots, the position of the blot of interest is indicated in the file name. [file elife-95407-fig6-data1.zip › Fig 6-source data 1/Figure 6B-source data LSUD-E Mg raw scan.tif]

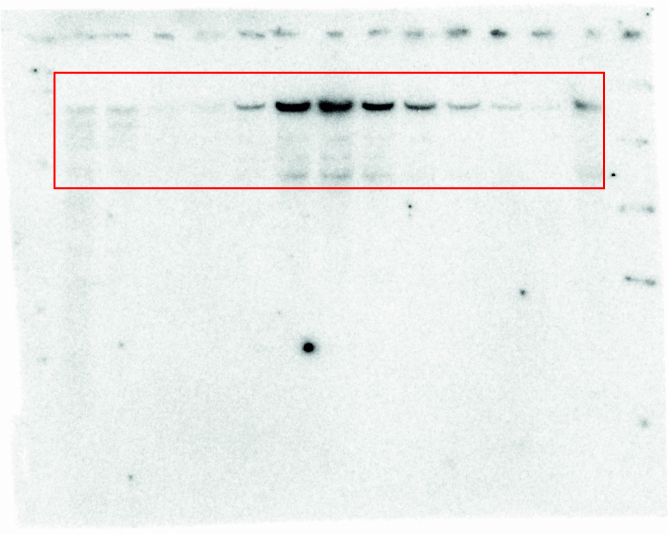

Supplement: Figure 6—source data 1. — Uncropped blots accompanied by images indicating the areas shown in Figure 6A–D with a red rectangle. In addition, raw scan images are provided. If the scan contains multiple blots, the position of the blot of interest is indicated in the file name. [file elife-95407-fig6-data1.zip › Fig 6-source data 1/Figure 6B-source data LSUF-G Mg blot labeled.jpg]

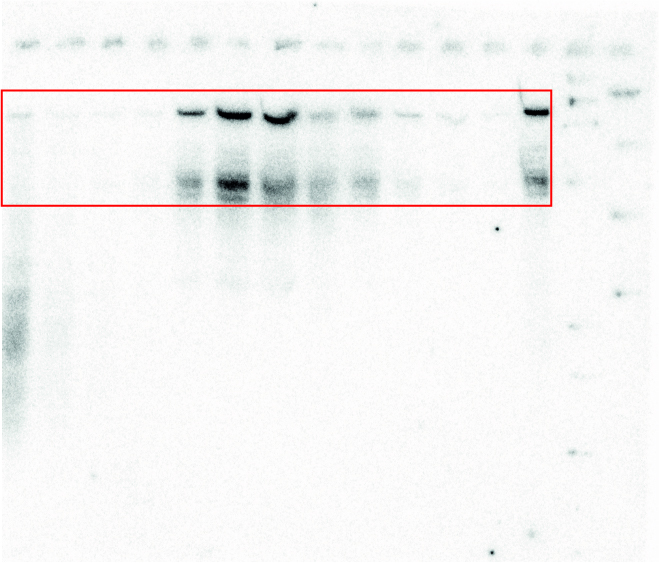

Supplement: Figure 6—source data 1. — Uncropped blots accompanied by images indicating the areas shown in Figure 6A–D with a red rectangle. In addition, raw scan images are provided. If the scan contains multiple blots, the position of the blot of interest is indicated in the file name. [file elife-95407-fig6-data1.zip › Fig 6-source data 1/Figure 6C-source data LSUF-G EDTA blot labeled.jpg]

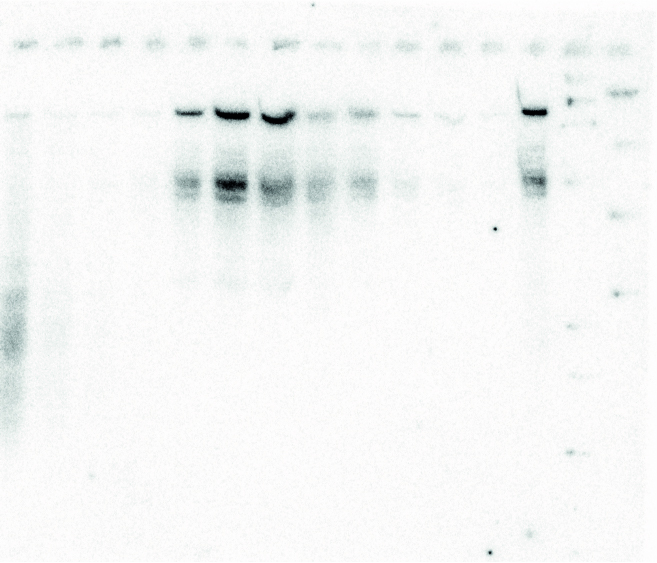

Supplement: Figure 6—source data 1. — Uncropped blots accompanied by images indicating the areas shown in Figure 6A–D with a red rectangle. In addition, raw scan images are provided. If the scan contains multiple blots, the position of the blot of interest is indicated in the file name. [file elife-95407-fig6-data1.zip › Fig 6-source data 1/Figure 6C-source data LSUF-G EDTA blot.jpg]

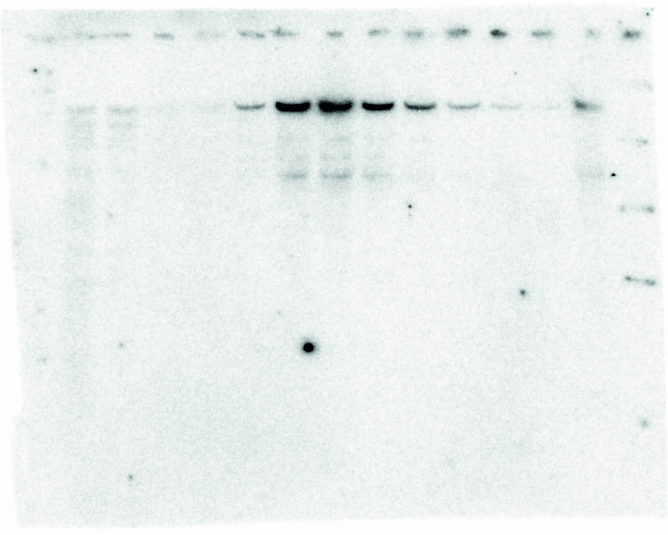

Supplement: Figure 6—source data 1. — Uncropped blots accompanied by images indicating the areas shown in Figure 6A–D with a red rectangle. In addition, raw scan images are provided. If the scan contains multiple blots, the position of the blot of interest is indicated in the file name. [file elife-95407-fig6-data1.zip › Fig 6-source data 1/Figure 6C-source data LSUF-G Mg blot.jpg]

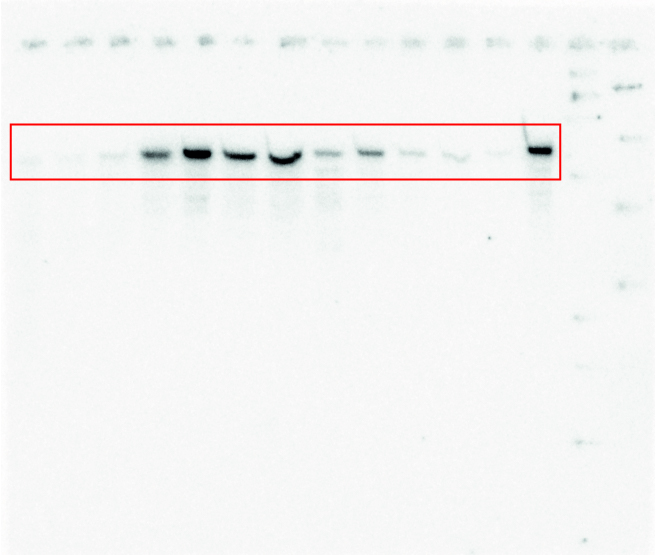

Supplement: Figure 6—source data 1. — Uncropped blots accompanied by images indicating the areas shown in Figure 6A–D with a red rectangle. In addition, raw scan images are provided. If the scan contains multiple blots, the position of the blot of interest is indicated in the file name. [file elife-95407-fig6-data1.zip › Fig 6-source data 1/Figure 6D-source data SSUA EDTA blot labeled.jpg]

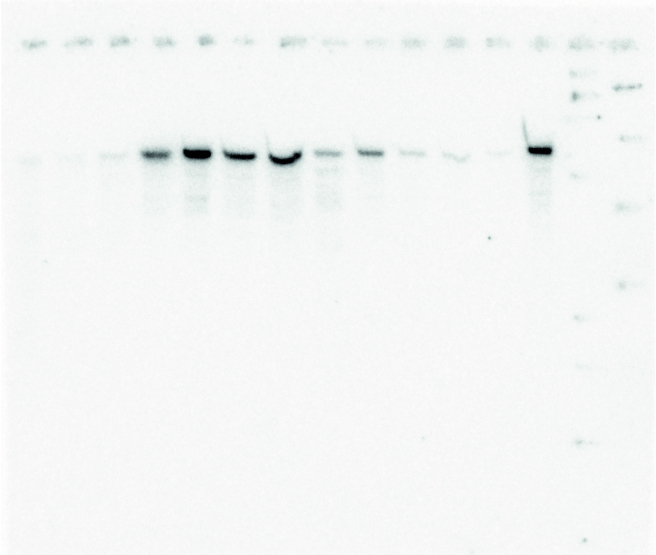

Supplement: Figure 6—source data 1. — Uncropped blots accompanied by images indicating the areas shown in Figure 6A–D with a red rectangle. In addition, raw scan images are provided. If the scan contains multiple blots, the position of the blot of interest is indicated in the file name. [file elife-95407-fig6-data1.zip › Fig 6-source data 1/Figure 6D-source data SSUA EDTA blot.jpg]

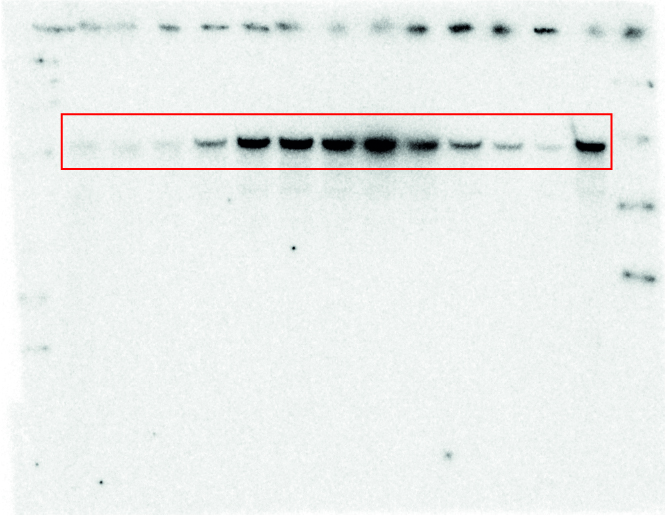

Supplement: Figure 6—source data 1. — Uncropped blots accompanied by images indicating the areas shown in Figure 6A–D with a red rectangle. In addition, raw scan images are provided. If the scan contains multiple blots, the position of the blot of interest is indicated in the file name. [file elife-95407-fig6-data1.zip › Fig 6-source data 1/Figure 6D-source data SSUA Mg blot labeled.jpg]

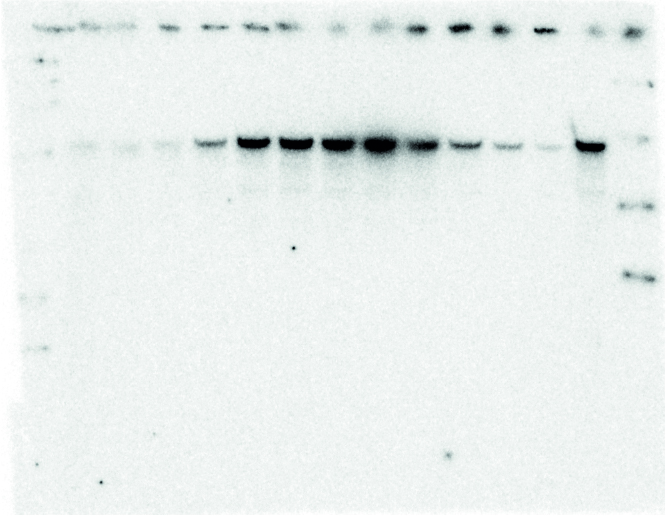

Supplement: Figure 6—source data 1. — Uncropped blots accompanied by images indicating the areas shown in Figure 6A–D with a red rectangle. In addition, raw scan images are provided. If the scan contains multiple blots, the position of the blot of interest is indicated in the file name. [file elife-95407-fig6-data1.zip › Fig 6-source data 1/Figure 6D-source data SSUA Mg blot.jpg]

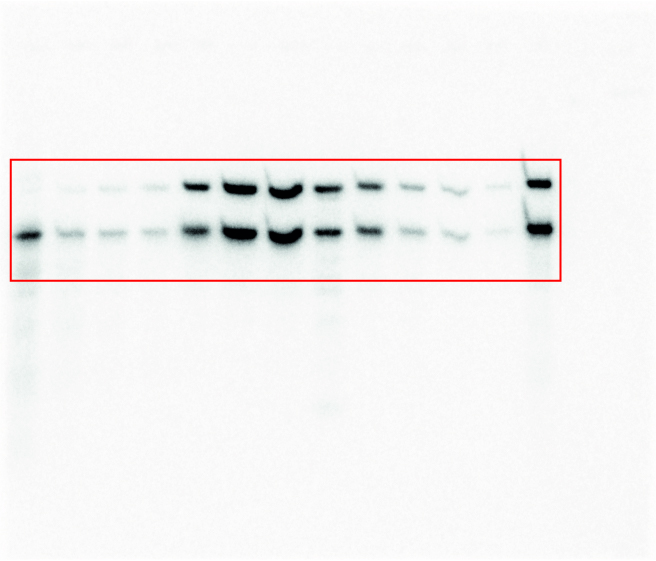

Supplement: Figure 6—source data 2. — Uncropped blots accompanied by images indicating the areas shown in Figure 6E–H with a red rectangle. In addition, raw scan images are provided. If the scan contains multiple blots, the position of the blot of interest is indicated in the file name. [file elife-95407-fig6-data2.zip › Fig 6-source data 2/Figure 6E-source data RNA1-2 EDTA blot labeled.jpg]

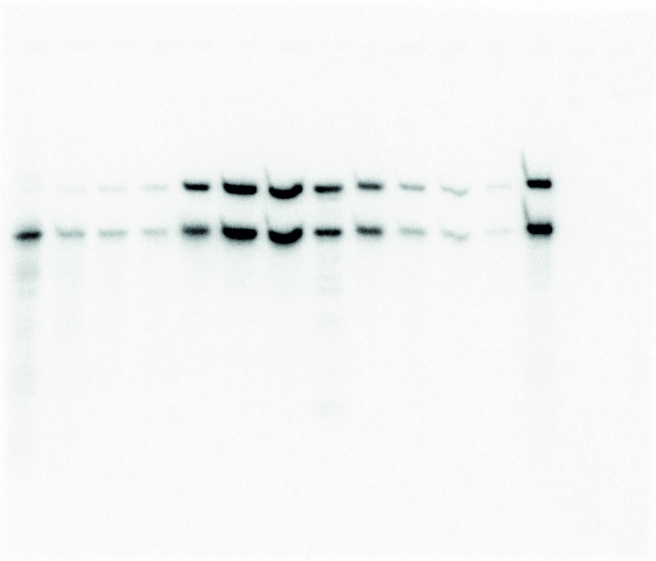

Supplement: Figure 6—source data 2. — Uncropped blots accompanied by images indicating the areas shown in Figure 6E–H with a red rectangle. In addition, raw scan images are provided. If the scan contains multiple blots, the position of the blot of interest is indicated in the file name. [file elife-95407-fig6-data2.zip › Fig 6-source data 2/Figure 6E-source data RNA1-2 EDTA blot.jpg]

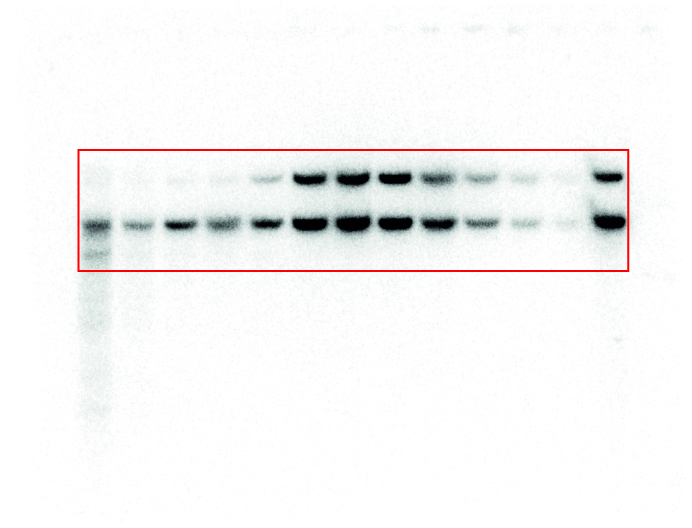

Supplement: Figure 6—source data 2. — Uncropped blots accompanied by images indicating the areas shown in Figure 6E–H with a red rectangle. In addition, raw scan images are provided. If the scan contains multiple blots, the position of the blot of interest is indicated in the file name. [file elife-95407-fig6-data2.zip › Fig 6-source data 2/Figure 6E-source data RNA1-2 Mg blot labeled.jpg]

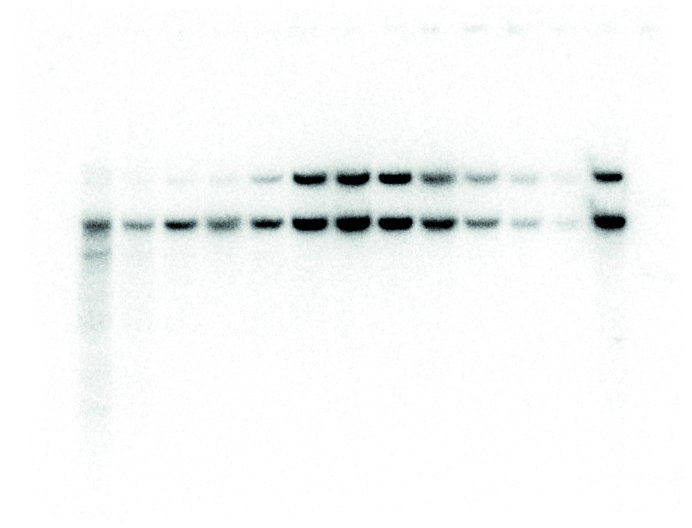

Supplement: Figure 6—source data 2. — Uncropped blots accompanied by images indicating the areas shown in Figure 6E–H with a red rectangle. In addition, raw scan images are provided. If the scan contains multiple blots, the position of the blot of interest is indicated in the file name. [file elife-95407-fig6-data2.zip › Fig 6-source data 2/Figure 6E-source data RNA1-2 Mg blot.jpg]

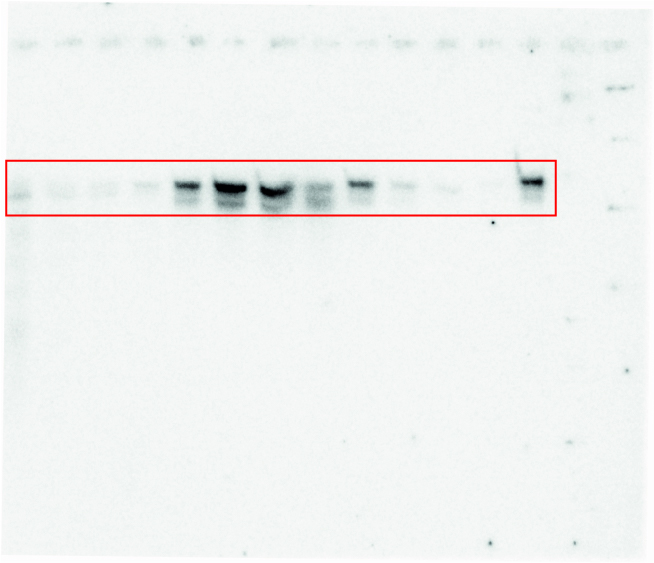

Supplement: Figure 6—source data 2. — Uncropped blots accompanied by images indicating the areas shown in Figure 6E–H with a red rectangle. In addition, raw scan images are provided. If the scan contains multiple blots, the position of the blot of interest is indicated in the file name. [file elife-95407-fig6-data2.zip › Fig 6-source data 2/Figure 6F-source data RNA3 EDTA blot labeled.jpg]

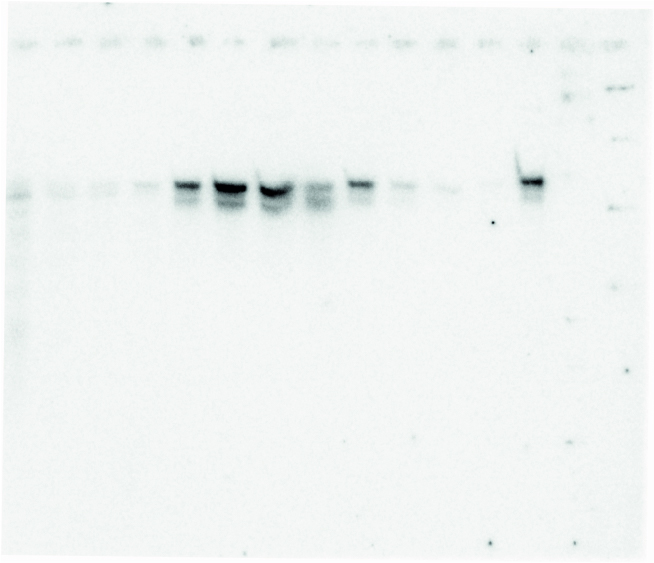

Supplement: Figure 6—source data 2. — Uncropped blots accompanied by images indicating the areas shown in Figure 6E–H with a red rectangle. In addition, raw scan images are provided. If the scan contains multiple blots, the position of the blot of interest is indicated in the file name. [file elife-95407-fig6-data2.zip › Fig 6-source data 2/Figure 6F-source data RNA3 EDTA blot.jpg]

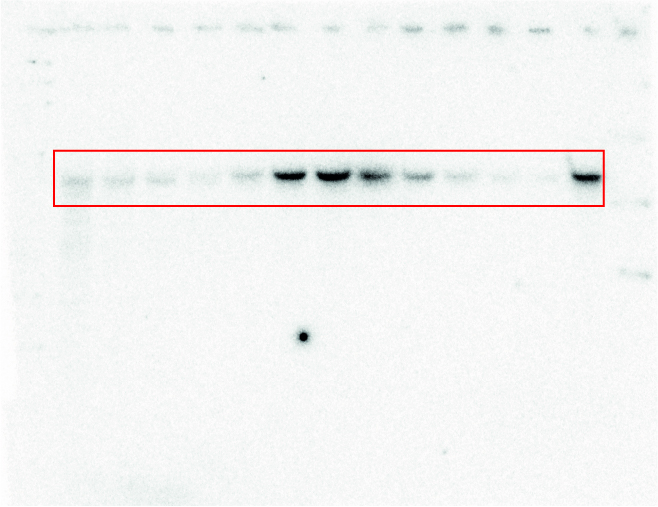

Supplement: Figure 6—source data 2. — Uncropped blots accompanied by images indicating the areas shown in Figure 6E–H with a red rectangle. In addition, raw scan images are provided. If the scan contains multiple blots, the position of the blot of interest is indicated in the file name. [file elife-95407-fig6-data2.zip › Fig 6-source data 2/Figure 6F-source data RNA3 Mg blot labeled.jpg]

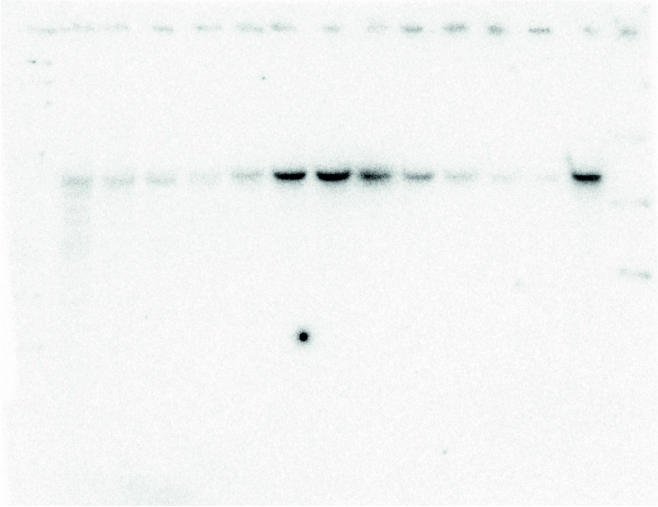

Supplement: Figure 6—source data 2. — Uncropped blots accompanied by images indicating the areas shown in Figure 6E–H with a red rectangle. In addition, raw scan images are provided. If the scan contains multiple blots, the position of the blot of interest is indicated in the file name. [file elife-95407-fig6-data2.zip › Fig 6-source data 2/Figure 6F-source data RNA3 Mg blot.jpg]

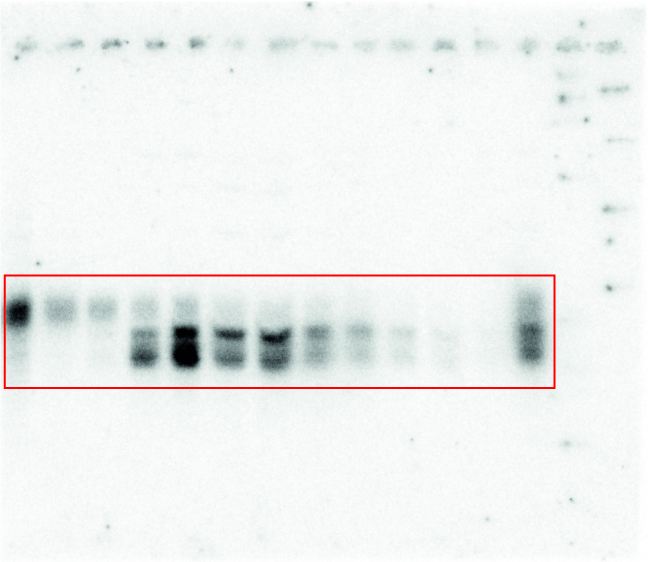

Supplement: Figure 6—source data 2. — Uncropped blots accompanied by images indicating the areas shown in Figure 6E–H with a red rectangle. In addition, raw scan images are provided. If the scan contains multiple blots, the position of the blot of interest is indicated in the file name. [file elife-95407-fig6-data2.zip › Fig 6-source data 2/Figure 6G-source data RNA19 blot EDTA labeled.jpg]

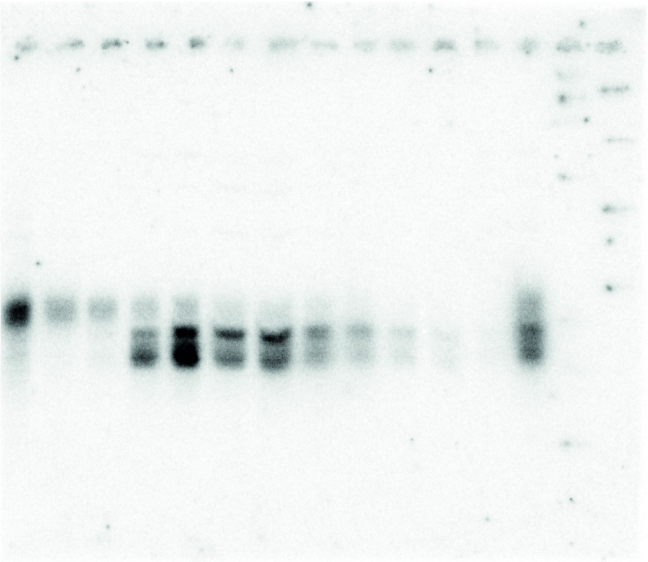

Supplement: Figure 6—source data 2. — Uncropped blots accompanied by images indicating the areas shown in Figure 6E–H with a red rectangle. In addition, raw scan images are provided. If the scan contains multiple blots, the position of the blot of interest is indicated in the file name. [file elife-95407-fig6-data2.zip › Fig 6-source data 2/Figure 6G-source data RNA19 blot EDTA.jpg]

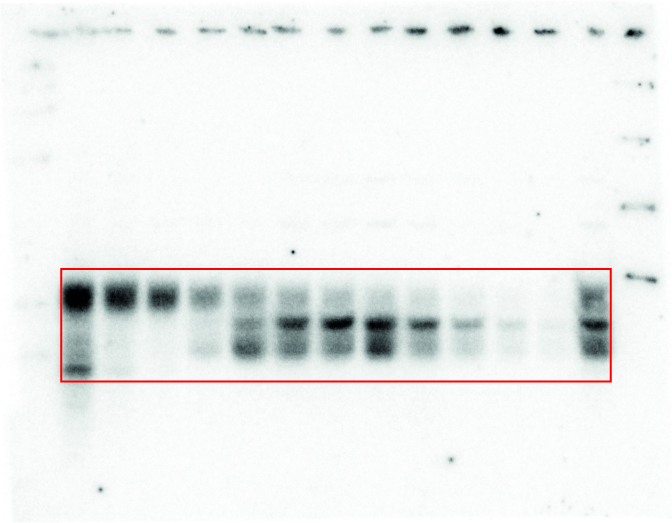

Supplement: Figure 6—source data 2. — Uncropped blots accompanied by images indicating the areas shown in Figure 6E–H with a red rectangle. In addition, raw scan images are provided. If the scan contains multiple blots, the position of the blot of interest is indicated in the file name. [file elife-95407-fig6-data2.zip › Fig 6-source data 2/Figure 6G-source data RNA19 blot Mg labeled.jpg]

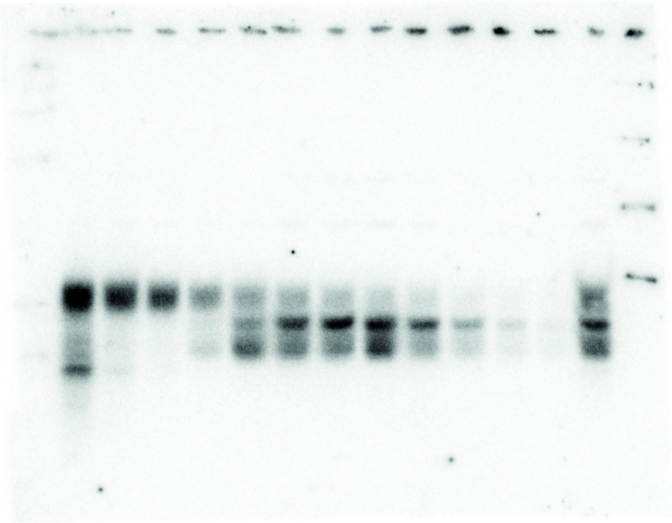

Supplement: Figure 6—source data 2. — Uncropped blots accompanied by images indicating the areas shown in Figure 6E–H with a red rectangle. In addition, raw scan images are provided. If the scan contains multiple blots, the position of the blot of interest is indicated in the file name. [file elife-95407-fig6-data2.zip › Fig 6-source data 2/Figure 6G-source data RNA19 blot Mg.jpg]

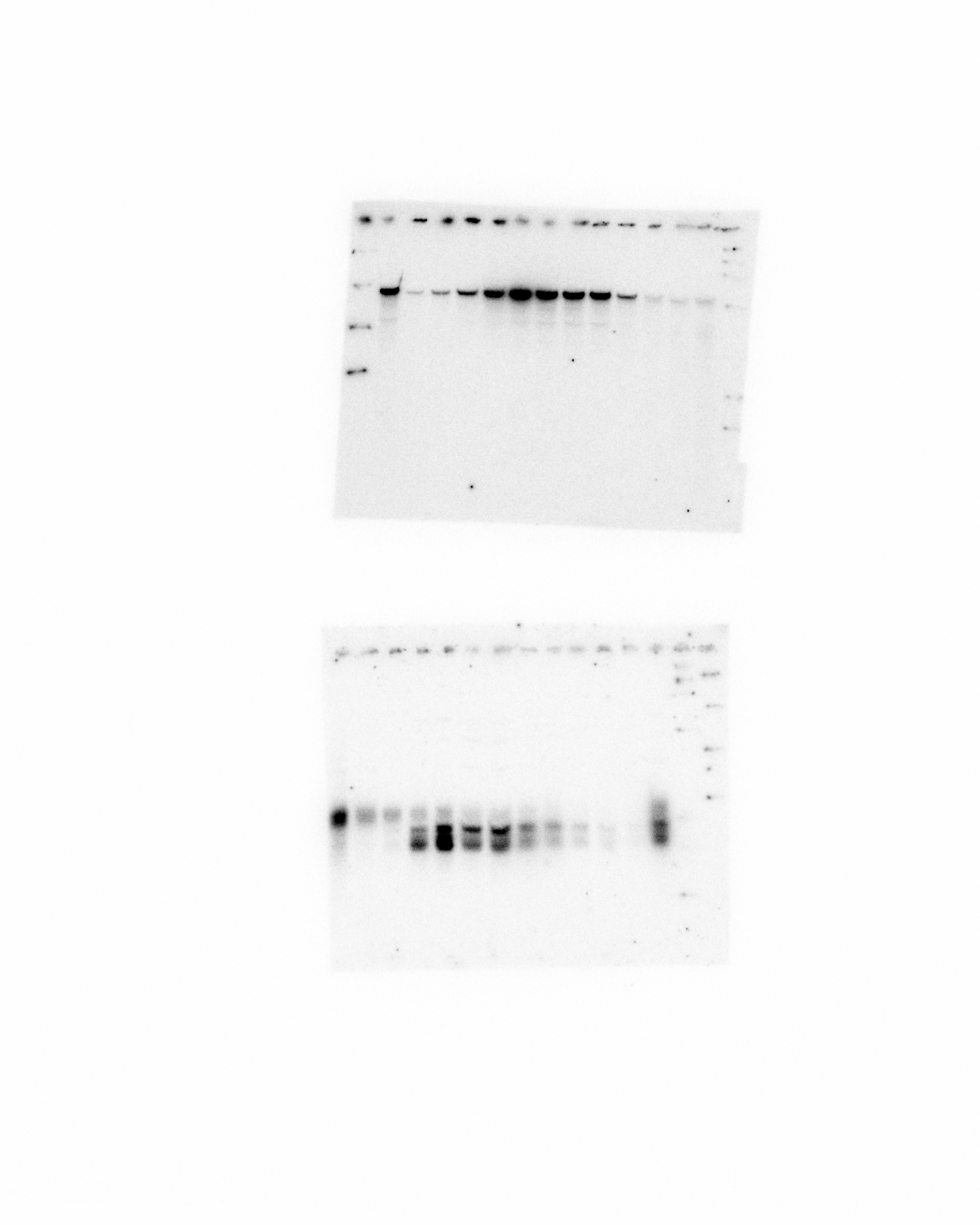

Supplement: Figure 6—source data 2. — Uncropped blots accompanied by images indicating the areas shown in Figure 6E–H with a red rectangle. In addition, raw scan images are provided. If the scan contains multiple blots, the position of the blot of interest is indicated in the file name. [file elife-95407-fig6-data2.zip › Fig 6-source data 2/Figure 6G-source data RNA19 EDTA raw scan (bottom).tif]

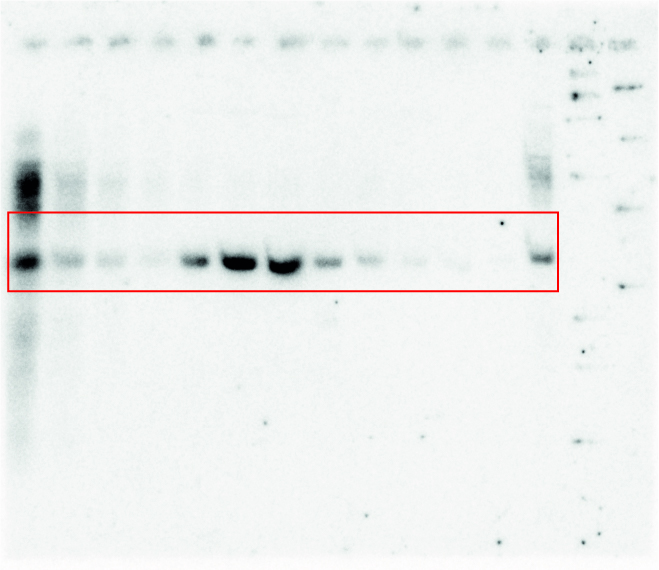

Supplement: Figure 6—source data 2. — Uncropped blots accompanied by images indicating the areas shown in Figure 6E–H with a red rectangle. In addition, raw scan images are provided. If the scan contains multiple blots, the position of the blot of interest is indicated in the file name. [file elife-95407-fig6-data2.zip › Fig 6-source data 2/Figure 6H-source data RNA29 EDTA blot labeled.jpg]

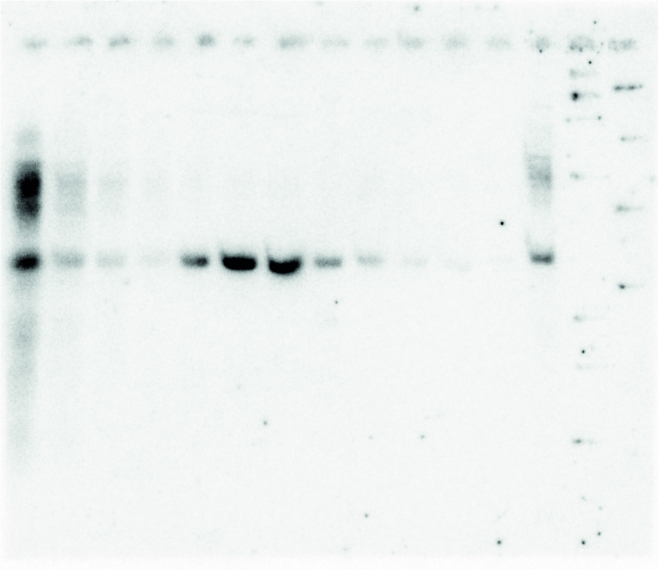

Supplement: Figure 6—source data 2. — Uncropped blots accompanied by images indicating the areas shown in Figure 6E–H with a red rectangle. In addition, raw scan images are provided. If the scan contains multiple blots, the position of the blot of interest is indicated in the file name. [file elife-95407-fig6-data2.zip › Fig 6-source data 2/Figure 6H-source data RNA29 EDTA blot.jpg]

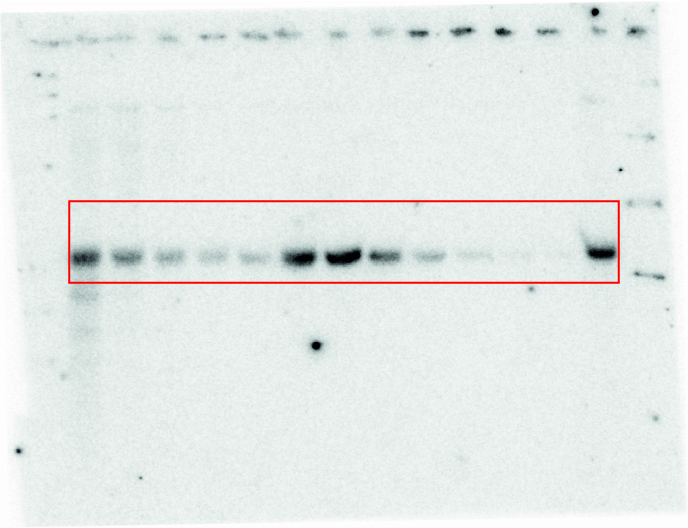

Supplement: Figure 6—source data 2. — Uncropped blots accompanied by images indicating the areas shown in Figure 6E–H with a red rectangle. In addition, raw scan images are provided. If the scan contains multiple blots, the position of the blot of interest is indicated in the file name. [file elife-95407-fig6-data2.zip › Fig 6-source data 2/Figure 6H-source data RNA29 Mg blot labeled.jpg]

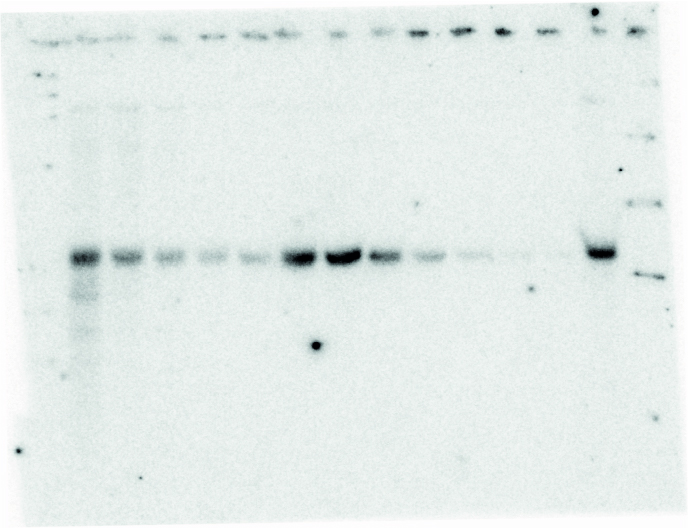

Supplement: Figure 6—source data 2. — Uncropped blots accompanied by images indicating the areas shown in Figure 6E–H with a red rectangle. In addition, raw scan images are provided. If the scan contains multiple blots, the position of the blot of interest is indicated in the file name. [file elife-95407-fig6-data2.zip › Fig 6-source data 2/Figure 6H-source data RNA29 Mg blot.jpg]

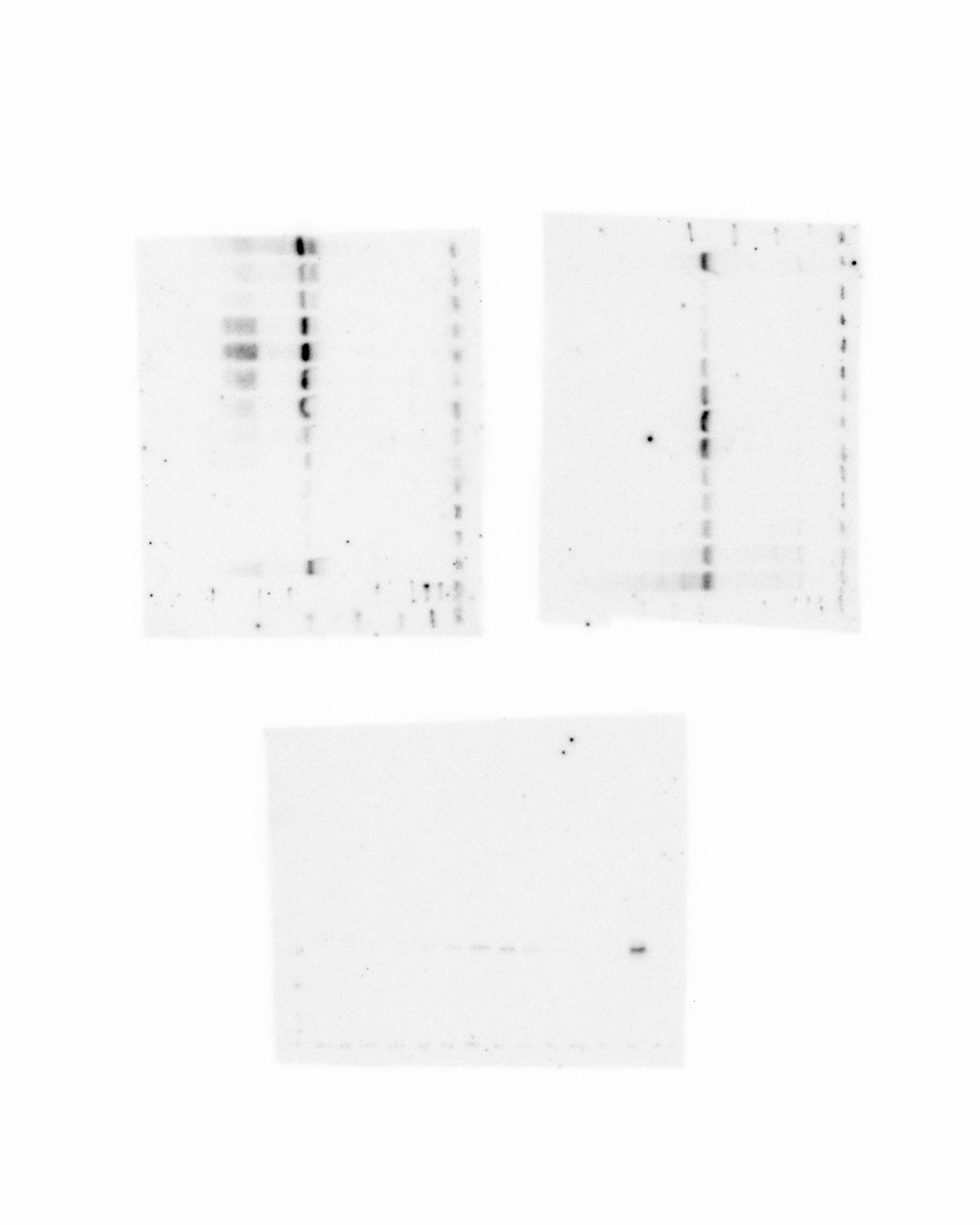

Supplement: Figure 6—source data 2. — Uncropped blots accompanied by images indicating the areas shown in Figure 6E–H with a red rectangle. In addition, raw scan images are provided. If the scan contains multiple blots, the position of the blot of interest is indicated in the file name. [file elife-95407-fig6-data2.zip › Fig 6-source data 2/Figure 6H-source data RNA29 Mg raw scan (top right).tif]

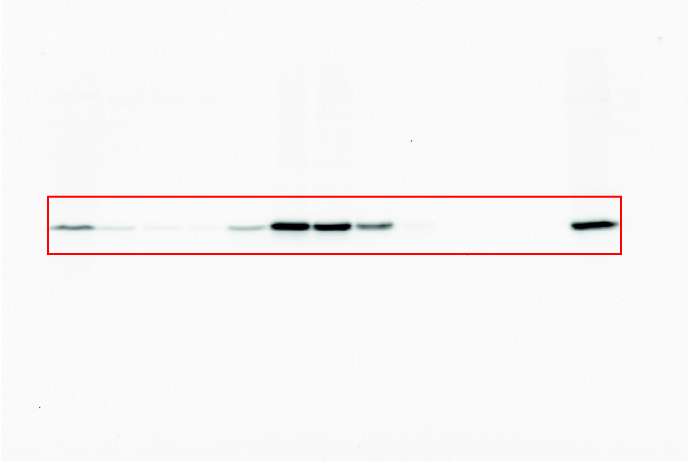

Supplement: Figure 8—source data 1. — Uncropped immunoblots accompanied by images indicating the areas shown in Figure 8B with a red rectangle. In addition, raw scan images and light image overlays depicting the membrane outline are provided. [file elife-95407-fig8-data1.zip › Fig 8-source data 1/Figure 8B-source data L11 blot EDTA labeled.jpg]

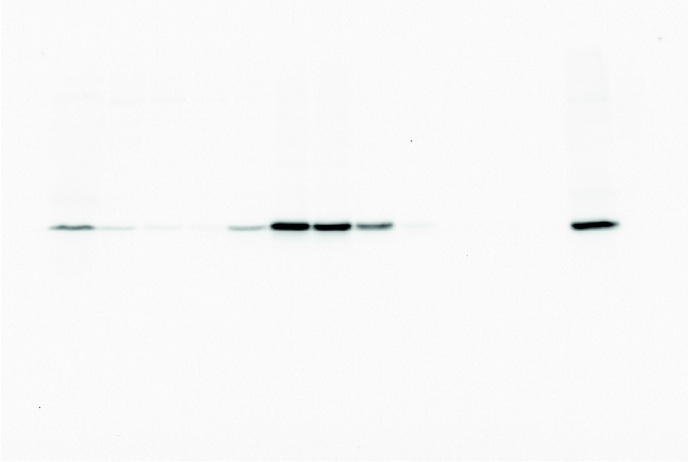

Supplement: Figure 8—source data 1. — Uncropped immunoblots accompanied by images indicating the areas shown in Figure 8B with a red rectangle. In addition, raw scan images and light image overlays depicting the membrane outline are provided. [file elife-95407-fig8-data1.zip › Fig 8-source data 1/Figure 8B-source data L11 blot EDTA.jpg]

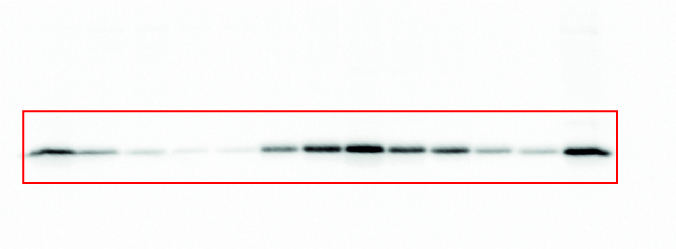

Supplement: Figure 8—source data 1. — Uncropped immunoblots accompanied by images indicating the areas shown in Figure 8B with a red rectangle. In addition, raw scan images and light image overlays depicting the membrane outline are provided. [file elife-95407-fig8-data1.zip › Fig 8-source data 1/Figure 8B-source data L11 blot Mg labeled.jpg]

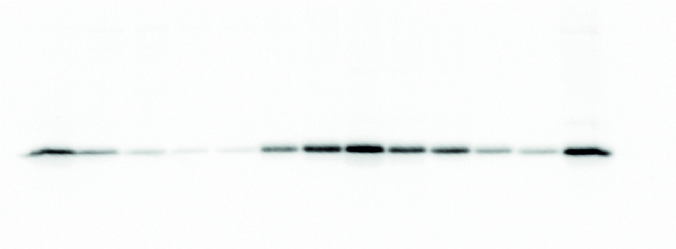

Supplement: Figure 8—source data 1. — Uncropped immunoblots accompanied by images indicating the areas shown in Figure 8B with a red rectangle. In addition, raw scan images and light image overlays depicting the membrane outline are provided. [file elife-95407-fig8-data1.zip › Fig 8-source data 1/Figure 8B-source data L11 blot Mg.jpg]

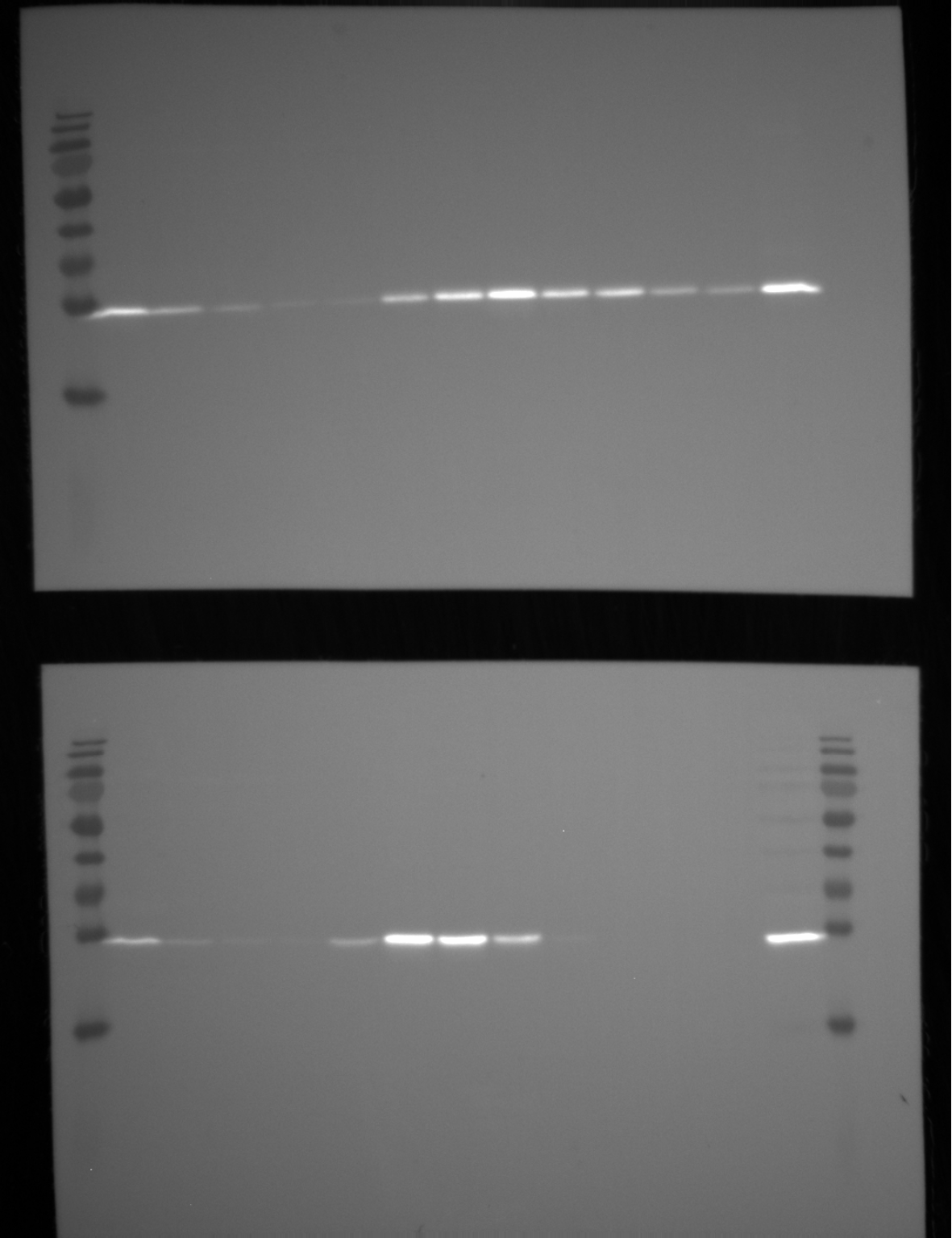

Supplement: Figure 8—source data 1. — Uncropped immunoblots accompanied by images indicating the areas shown in Figure 8B with a red rectangle. In addition, raw scan images and light image overlays depicting the membrane outline are provided. [file elife-95407-fig8-data1.zip › Fig 8-source data 1/Figure 8B-source data L11 Mg EDTA raw scan light overlay.tif]

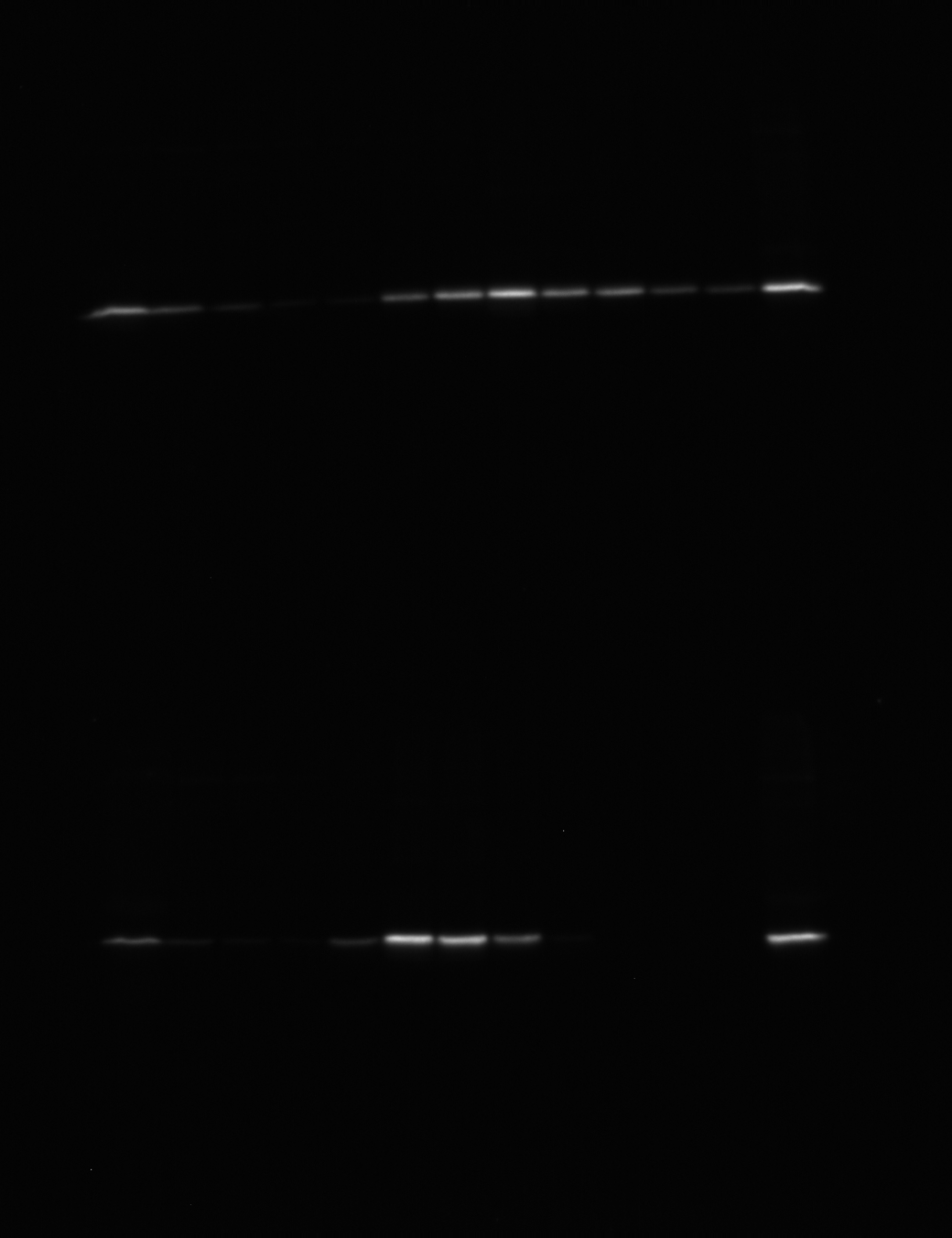

Supplement: Figure 8—source data 1. — Uncropped immunoblots accompanied by images indicating the areas shown in Figure 8B with a red rectangle. In addition, raw scan images and light image overlays depicting the membrane outline are provided. [file elife-95407-fig8-data1.zip › Fig 8-source data 1/Figure 8B-source data L11 Mg EDTA raw scan.tif]

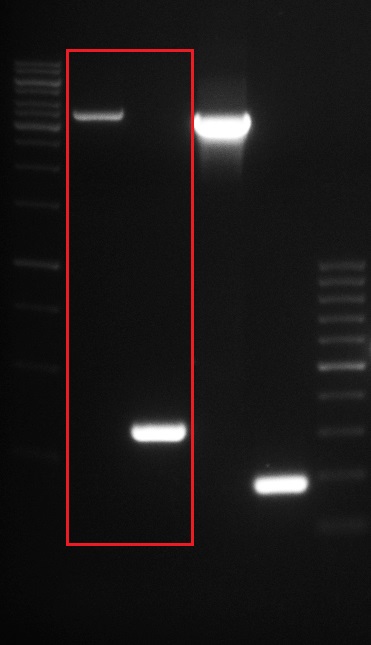

Supplement: Figure 8—figure supplement 1—source data 1. — Uncropped blots and gels accompanied by images indicating the areas shown in Figure 8—figure supplement 1B–C with a red rectangle. In addition, raw scan images are provided. Additionally, for immunoblots light image overlays depicting the membrane outline are provided. [file elife-95407-fig8-figsupp1-data1.zip › Figure 8-figure supplement 1-source data 1/Figure 8-figure supplement 1B-source data L11 gel raw labeled.jpg]

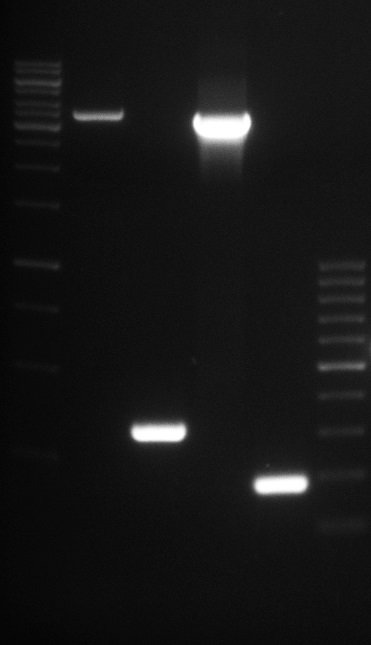

Supplement: Figure 8—figure supplement 1—source data 1. — Uncropped blots and gels accompanied by images indicating the areas shown in Figure 8—figure supplement 1B–C with a red rectangle. In addition, raw scan images are provided. Additionally, for immunoblots light image overlays depicting the membrane outline are provided. [file elife-95407-fig8-figsupp1-data1.zip › Figure 8-figure supplement 1-source data 1/Figure 8-figure supplement 1B-source data L11 gel raw.jpg]

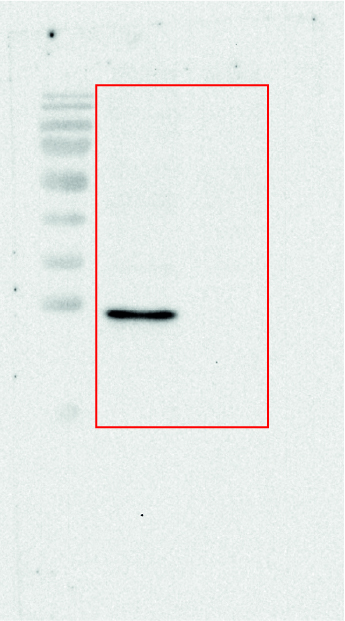

Supplement: Figure 8—figure supplement 1—source data 1. — Uncropped blots and gels accompanied by images indicating the areas shown in Figure 8—figure supplement 1B–C with a red rectangle. In addition, raw scan images are provided. Additionally, for immunoblots light image overlays depicting the membrane outline are provided. [file elife-95407-fig8-figsupp1-data1.zip › Figure 8-figure supplement 1-source data 1/Figure 8-figure supplement 1C-source data L11 blot labeled.jpg]

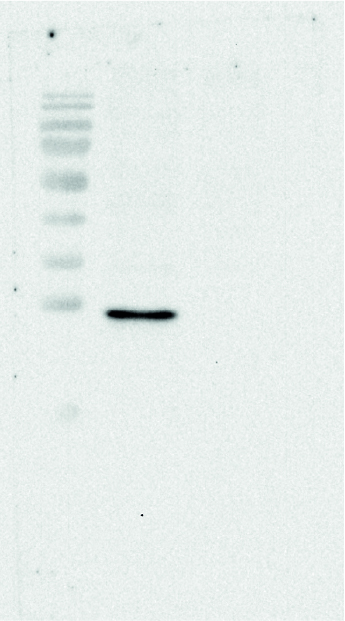

Supplement: Figure 8—figure supplement 1—source data 1. — Uncropped blots and gels accompanied by images indicating the areas shown in Figure 8—figure supplement 1B–C with a red rectangle. In addition, raw scan images are provided. Additionally, for immunoblots light image overlays depicting the membrane outline are provided. [file elife-95407-fig8-figsupp1-data1.zip › Figure 8-figure supplement 1-source data 1/Figure 8-figure supplement 1C-source data L11 blot.jpg]

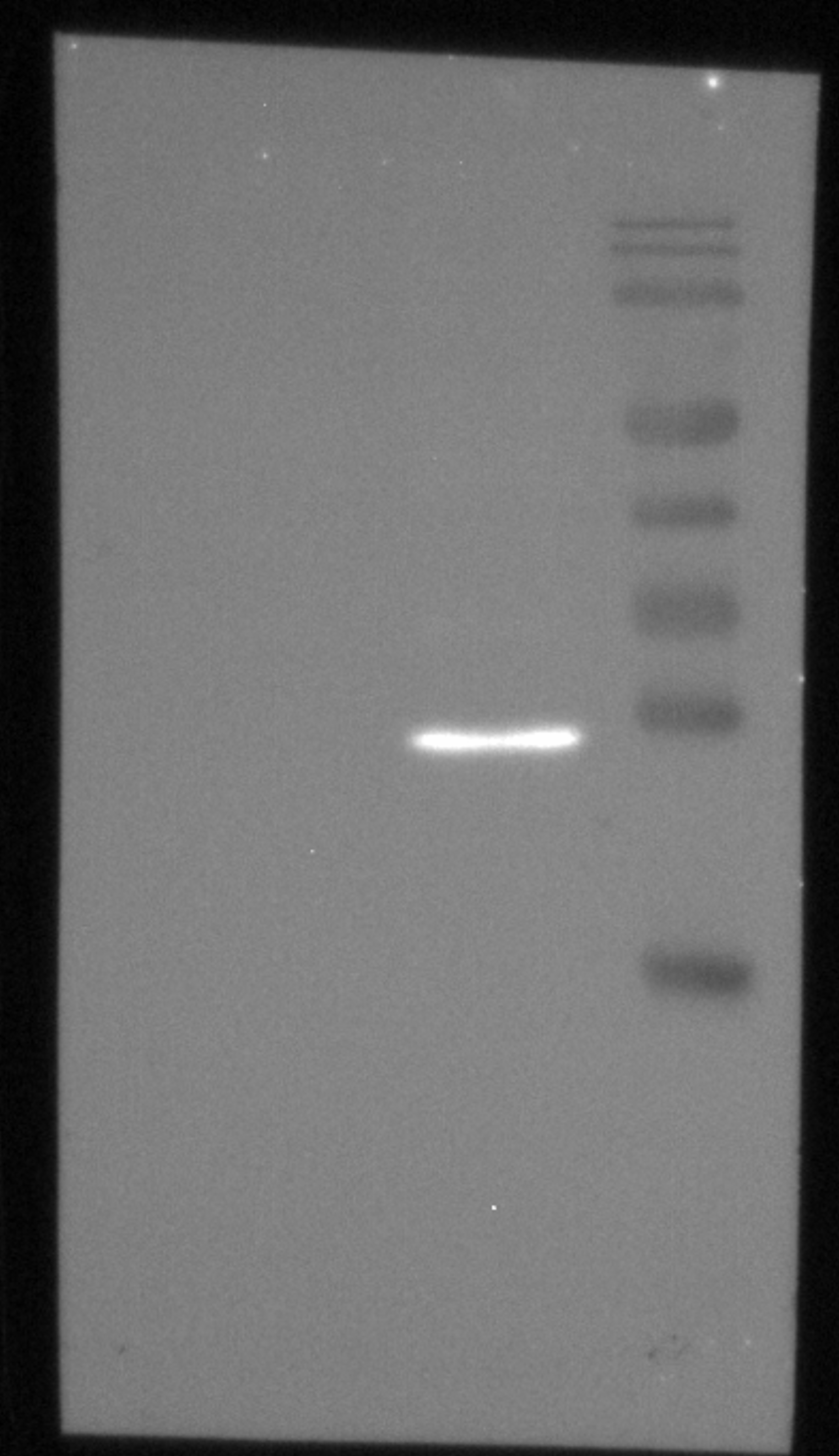

Supplement: Figure 8—figure supplement 1—source data 1. — Uncropped blots and gels accompanied by images indicating the areas shown in Figure 8—figure supplement 1B–C with a red rectangle. In addition, raw scan images are provided. Additionally, for immunoblots light image overlays depicting the membrane outline are provided. [file elife-95407-fig8-figsupp1-data1.zip › Figure 8-figure supplement 1-source data 1/Figure 8-figure supplement 1C-source data L11 raw scan light overlay.tif]

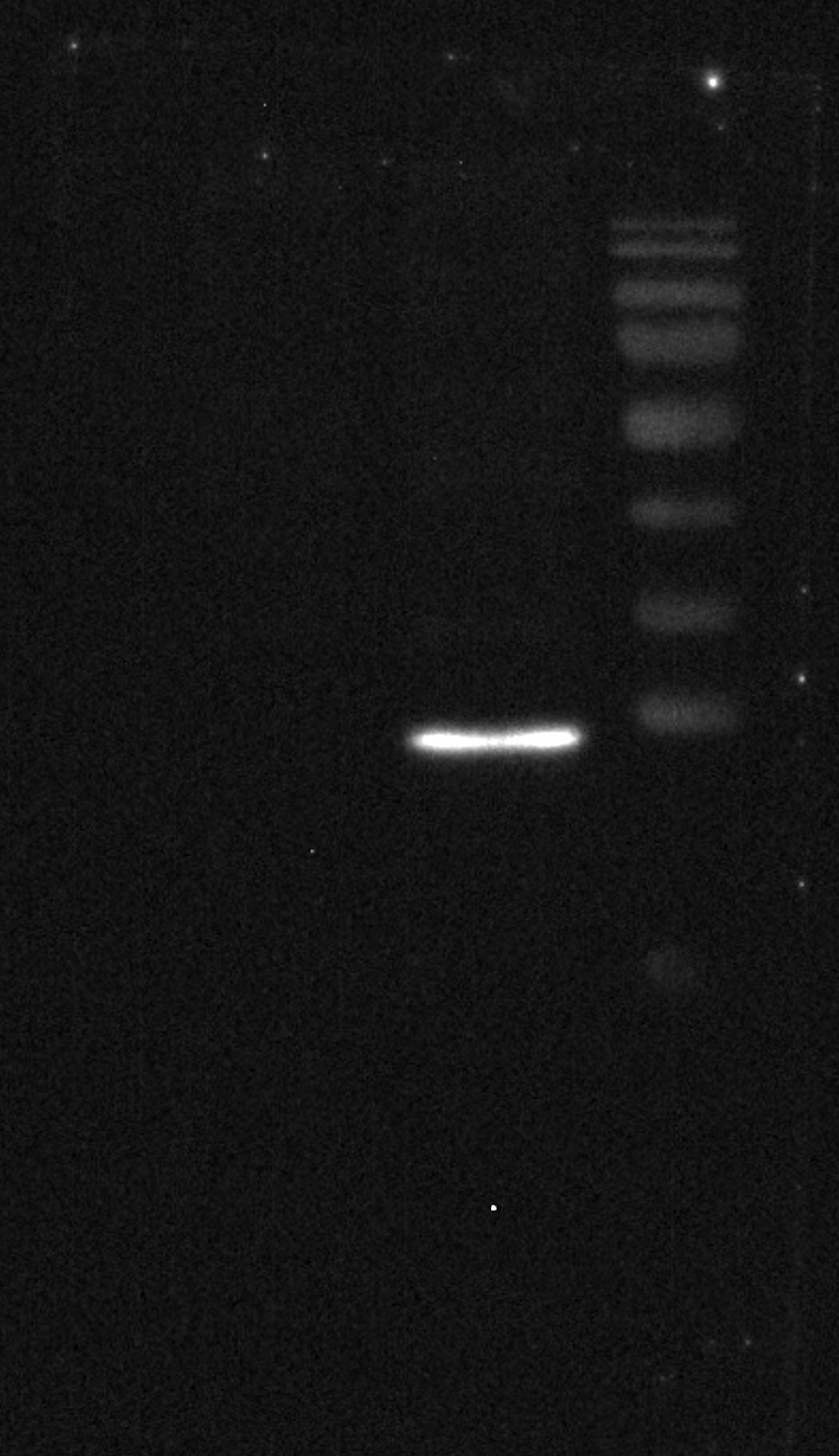

Supplement: Figure 8—figure supplement 1—source data 1. — Uncropped blots and gels accompanied by images indicating the areas shown in Figure 8—figure supplement 1B–C with a red rectangle. In addition, raw scan images are provided. Additionally, for immunoblots light image overlays depicting the membrane outline are provided. [file elife-95407-fig8-figsupp1-data1.zip › Figure 8-figure supplement 1-source data 1/Figure 8-figure supplement 1C-source data L11 raw scan.tif]

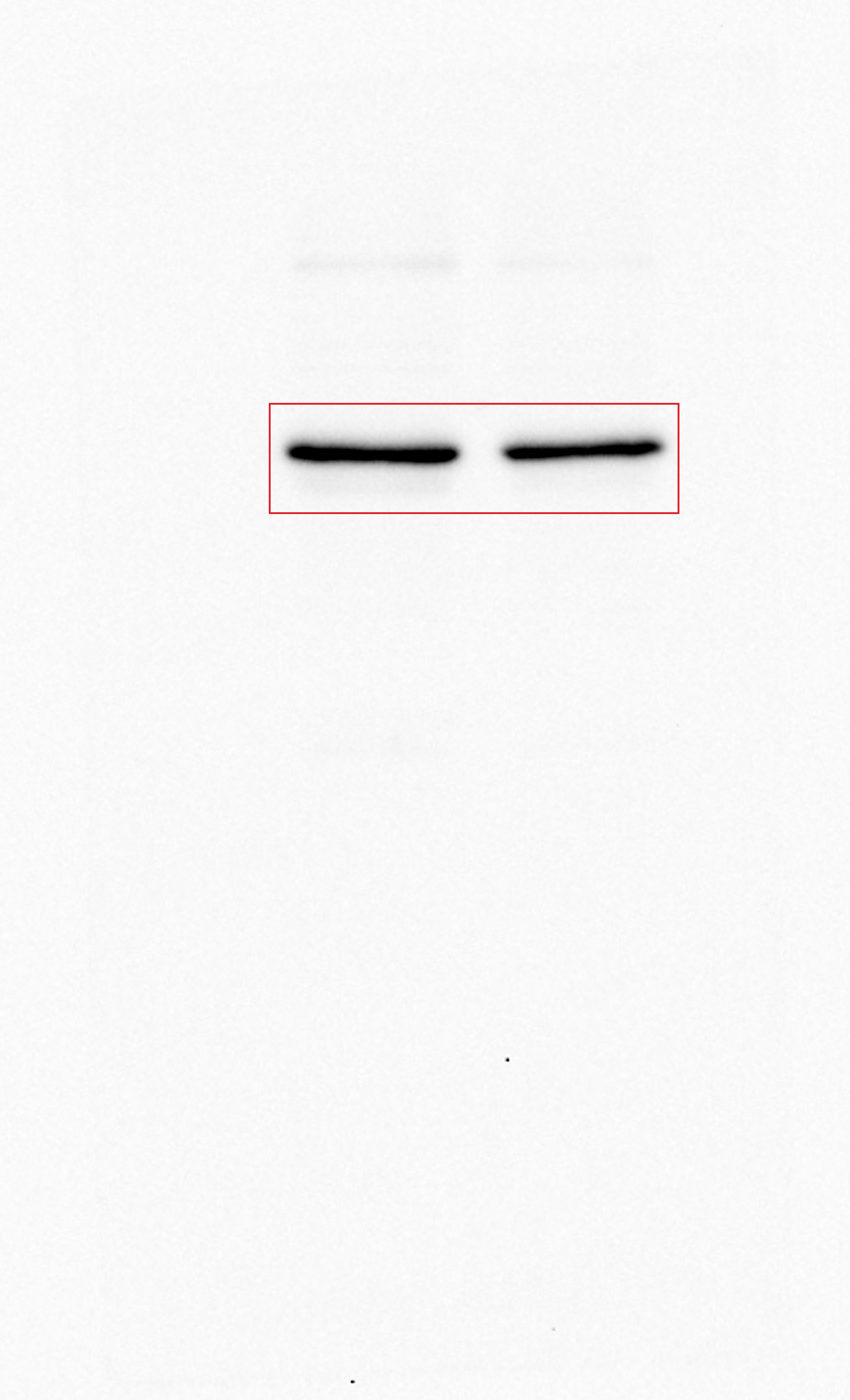

Supplement: Figure 8—figure supplement 1—source data 1. — Uncropped blots and gels accompanied by images indicating the areas shown in Figure 8—figure supplement 1B–C with a red rectangle. In addition, raw scan images are provided. Additionally, for immunoblots light image overlays depicting the membrane outline are provided. [file elife-95407-fig8-figsupp1-data1.zip › Figure 8-figure supplement 1-source data 1/Figure 8-figure supplement 1C-source data Tom40 blot labeled.jpg]

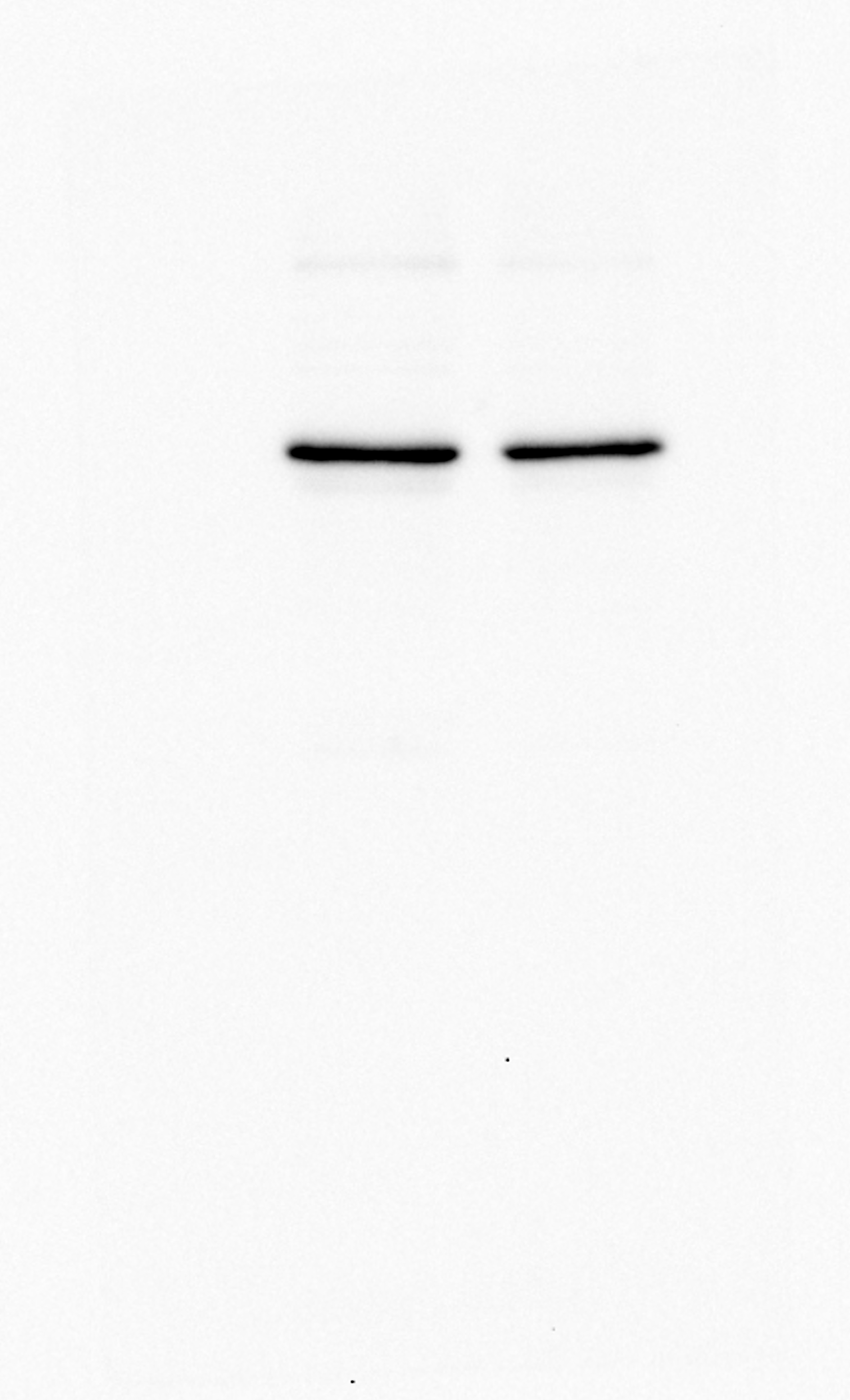

Supplement: Figure 8—figure supplement 1—source data 1. — Uncropped blots and gels accompanied by images indicating the areas shown in Figure 8—figure supplement 1B–C with a red rectangle. In addition, raw scan images are provided. Additionally, for immunoblots light image overlays depicting the membrane outline are provided. [file elife-95407-fig8-figsupp1-data1.zip › Figure 8-figure supplement 1-source data 1/Figure 8-figure supplement 1C-source data Tom40 blot.jpg]

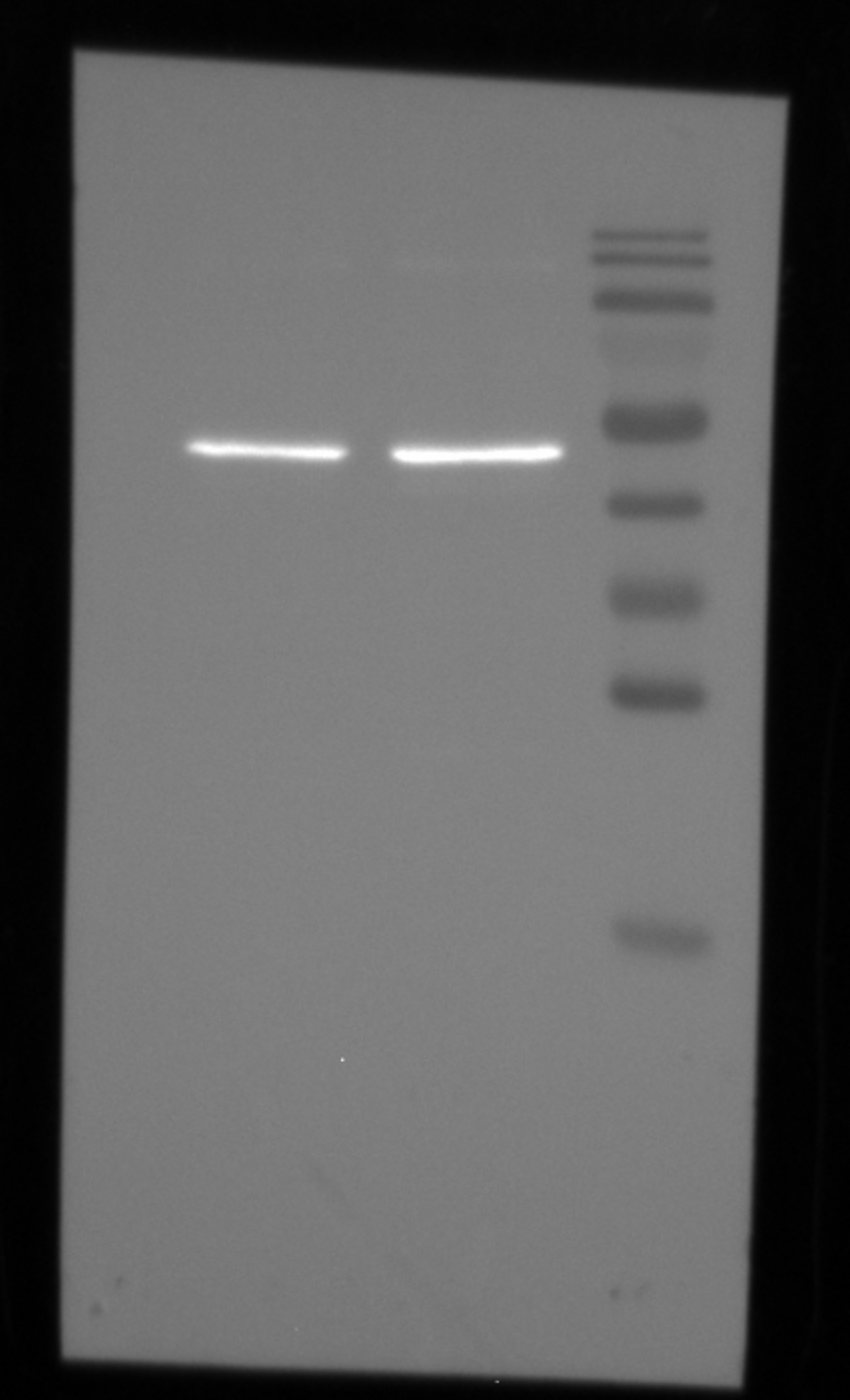

Supplement: Figure 8—figure supplement 1—source data 1. — Uncropped blots and gels accompanied by images indicating the areas shown in Figure 8—figure supplement 1B–C with a red rectangle. In addition, raw scan images are provided. Additionally, for immunoblots light image overlays depicting the membrane outline are provided. [file elife-95407-fig8-figsupp1-data1.zip › Figure 8-figure supplement 1-source data 1/Figure 8-figure supplement 1C-source dataTom40 raw scan light overlay.tif]

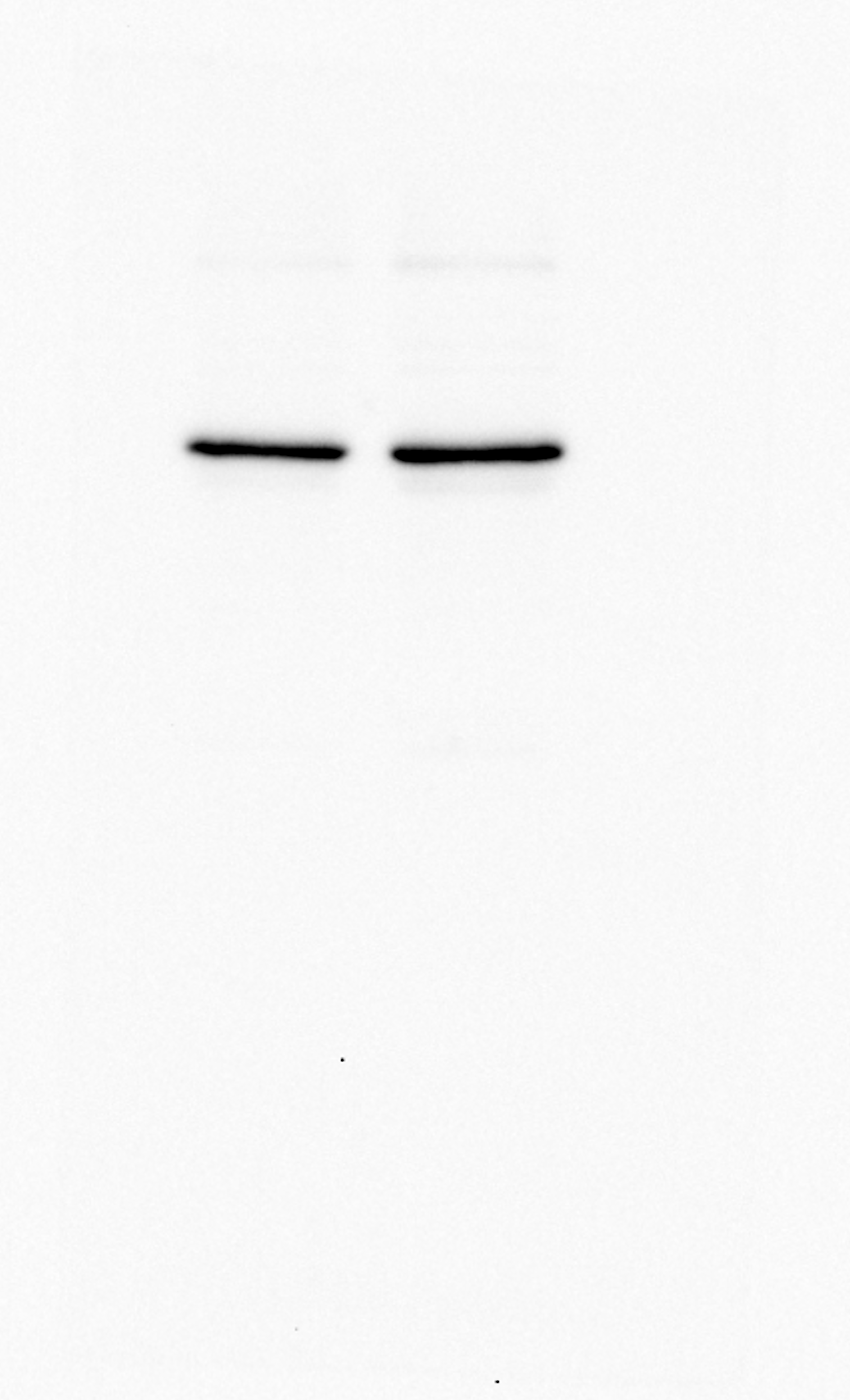

Supplement: Figure 8—figure supplement 1—source data 1. — Uncropped blots and gels accompanied by images indicating the areas shown in Figure 8—figure supplement 1B–C with a red rectangle. In addition, raw scan images are provided. Additionally, for immunoblots light image overlays depicting the membrane outline are provided. [file elife-95407-fig8-figsupp1-data1.zip › Figure 8-figure supplement 1-source data 1/Figure 8-figure supplement 1C-source dataTom40 raw scan.tif]
