# Supplementary material for: Small RNAs from mitochondrial genome recombination sites are incorporated into T. gondii mitoribosomes
Source: eLife. 2024 Feb 16;13:e95407. doi: 10.7554/eLife.95407 (PMC10948144; doi:10.7554/eLife.95407)
Supplement: Supplementary file 1. [file elife-95407-supp1.docx]

**Supplementary file 1: ONT sequencing mapping statistics on nuclear and organellar genomes for data obtained in this study and data previously published [1].**

|  | **total length (nt)** | | | |
| --- | --- | --- | --- | --- |
|  | **this study RH** | **this study mt reads >1000nt** | **Namasivayam et al. ME49** | **Namasivayam et al.**  **RH Δuprt** |
| *raw reads total* | *1.060.579.347* |  |  |  |
| *others* | *184.244.447* |  |  |  |
| *apicoplast* | *22.322.153* |  |  |  |
| mt | 78.540.610 | 48.661.147 | 699.177 | 1.985.929 |
| nucleus | 775.472.137 | 805.351.600 | 313.953.499 | 4.309.669.177 |
| mt + nucleus | 854.012.747 | 854.012.747 | 314.652.676 | 4.311.655.106 |
| **% mt** | **9,20** | **5,70** | **0,22** | **0,05** |
|  | **#reads** | | | |
|  | **this study RH** | **this study mt reads >1000nt** | **Namasivayam et al. ME49** | **Namasivayam et al.**  **RH Δuprt** |
| *raw reads total* | *2.080.000* |  |  |  |
| *others* | *299.025* |  |  |  |
| *apicoplast* | *8.152* |  |  |  |
| mt | 86.761 | 23.693 | 269 | 779 |
| nucleus | 1.686.062 | 1.749.130 | 43.392 | 765.200 |
| mt + nucleus | 1.772.823 | 1.772.823 | 43.661 | 765.979 |
| **% mt** | **4,89** | **1,34** | **0,62** | **0,10** |
