# Supplementary material for: Small RNAs from mitochondrial genome recombination sites are incorporated into T. gondii mitoribosomes
Source: eLife. 2024 Feb 16;13:e95407. doi: 10.7554/eLife.95407 (PMC10948144; doi:10.7554/eLife.95407)
Supplement: Supplementary file 2. [file elife-95407-supp2.docx]

**Supplementary file 2: Read length distribution of *T. gondii* mitochondrial ONT reads.**

| **Read length (nt)** | **Number of reads** |
| --- | --- |
| 0 - 150 | 103 |
| 150 - 500 | 38,343 |
| 500 - 1,000 | 24,643 |
| 1,000 - 2,000 | 15,402 |
| 2,000 - 3,000 | 4,710 |
| 3,000 - 4,000 | 1,864 |
| 4,000 - 5,000 | 860 |
| 5,000 - 6,000 | 399 |
| 6,000 - 7,000 | 183 |
| 7,000 - 8,000 | 118 |
| 8,000 - 9,000 | 53 |
| 9,000 - 10,000 | 38 |
| >10,000 | 45 |
| **total** | 86,761 |
