# Supplementary material for: Small RNAs from mitochondrial genome recombination sites are incorporated into T. gondii mitoribosomes
Source: eLife. 2024 Feb 16;13:e95407. doi: 10.7554/eLife.95407 (PMC10948144; doi:10.7554/eLife.95407)
Supplement: Supplementary file 3. [file elife-95407-supp3.docx]

**Supplementary file 3: Mitochondrial sequence block frequencies in ONT DNA sequencing data.**

| **Block** | **Number** |
| --- | --- |
| A | 19794 |
| B | 21737 |
| C | 3138 |
| D | 14076 |
| E | 5703 |
| F | 12089 |
| Fp | 21286 |
| H | 3572 |
| I | 5637 |
| J | 45849 |
| K | 6629 |
| Kp | 19398 |
| L | 22564 |
| M | 4947 |
| Mp | 21991 |
| N | 8393 |
| O | 21814 |
| P | 9940 |
| Q | 14275 |
| R | 12662 |
| S | 18294 |
| T | 8780 |
| U | 9431 |
| V | 33337 |
