# Supplementary material for: Small RNAs from mitochondrial genome recombination sites are incorporated into T. gondii mitoribosomes
Source: eLife. 2024 Feb 16;13:e95407. doi: 10.7554/eLife.95407 (PMC10948144; doi:10.7554/eLife.95407)
Supplement: Supplementary file 4. — Combinations that were found less than 50 times are considered false positives and shown in gray. [file elife-95407-supp4.docx]

**Supplementary file 4: Sequence block combinations identified in *T. gondii* mitochondrial ONT reads using a custom R script.**Combinations that were found less than 50-times are considered false-positives and shown in grey.

| **1st block** | **direction** | **2nd block** | **direction** | **n** | **orientation** |
| --- | --- | --- | --- | --- | --- |
| J | forward | B | forward | 19622 | head-tail |
| J | reverse | O | forward | 19528 | tail-tail |
| B | forward | Mp | forward | 19378 | head-tail |
| L | forward | J | forward | 19307 | head-tail |
| L | reverse | V | forward | 18377 | tail-tail |
| V | forward | S | forward | 14445 | head-tail |
| J | forward | Q | reverse | 12391 | head-head |
| V | forward | D | forward | 11757 | head-tail |
| D | forward | Kp | forward | 11741 | head-tail |
| Fp | forward | R | forward | 11400 | head-tail |
| O | forward | F | forward | 11044 | head-tail |
| F | forward | Fp | forward | 10765 | head-tail |
| R | forward | S | reverse | 9320 | head-head |
| Mp | forward | U | reverse | 7858 | head-head |
| Fp | reverse | N | forward | 7301 | tail-tail |
| Kp | forward | U | forward | 6969 | head-tail |
| A | forward | P | forward | 6957 | head-tail |
| T | forward | V | forward | 6858 | head-tail |
| N | forward | A | forward | 6345 | head-tail |
| A | forward | T | forward | 6320 | head-tail |
| K | forward | O | reverse | 5680 | head-head |
| Kp | forward | K | forward | 5447 | head-tail |
| I | forward | Kp | forward | 4599 | head-tail |
| J | forward | E | forward | 4345 | head-tail |
| P | forward | I | forward | 4274 | head-tail |
| E | forward | A | forward | 4248 | head-tail |
| Mp | forward | M | forward | 4078 | head-tail |
| Fp | forward | M | reverse | 3989 | head-head |
| H | forward | Q | forward | 3060 | head-tail |
| P | forward | H | forward | 2525 | head-tail |
| C | forward | Q | forward | 2293 | head-tail |
| S | forward | C | forward | 2287 | head-tail |
| J | reverse | R | forward | 45 | tail-tail |
| V | forward | V | reverse | 18 | head-head |
| Fp | forward | O | forward | 14 | head-tail |
| D | forward | A | forward | 10 | head-tail |
| D | forward | D | reverse | 6 | head-head |
| I | forward | S | forward | 6 | head-tail |
| S | forward | E | forward | 5 | head-tail |
| Kp | reverse | Kp | forward | 5 | tail-tail |
| J | reverse | J | forward | 5 | tail-tail |
| D | forward | I | reverse | 3 | head-head |
| S | forward | M | forward | 3 | head-tail |
| S | reverse | S | forward | 3 | tail-tail |
| B | forward | Mp | reverse | 2 | head-head |
| Q | forward | A | forward | 2 | head-tail |
| T | forward | L | forward | 2 | head-tail |
| V | forward | Kp | forward | 2 | head-tail |
| Q | forward | B | forward | 2 | head-tail |
| S | reverse | C | forward | 2 | tail-tail |
| S | reverse | D | forward | 2 | tail-tail |
| P | reverse | P | forward | 2 | tail-tail |
| A | forward | E | reverse | 1 | head-head |
| B | forward | V | forward | 1 | head-tail |
| F | forward | O | reverse | 1 | head-head |
| I | forward | J | forward | 1 | head-tail |
| I | forward | P | reverse | 1 | head-head |
| J | forward | Mp | reverse | 1 | head-head |
| Kp | forward | Kp | reverse | 1 | head-head |
| L | forward | O | forward | 1 | head-tail |
| Mp | forward | C | forward | 1 | head-tail |
| P | forward | P | reverse | 1 | head-head |
| Q | forward | A | reverse | 1 | head-head |
| S | forward | S | reverse | 1 | head-head |
| S | forward | F | reverse | 1 | head-head |
| U | forward | J | reverse | 1 | head-head |
| Q | reverse | Q | forward | 1 | tail-tail |
| Kp | reverse | K | forward | 1 | tail-tail |
| B | reverse | Mp | forward | 1 | tail-tail |
| A | reverse | A | forward | 1 | tail-tail |
| L | forward | V | forward | 1 | head-tail |
| P | reverse | T | forward | 1 | tail-tail |
| L | reverse | L | forward | 1 | tail-tail |
| L | reverse | Mp | forward | 1 | tail-tail |
| J | forward | Mp | forward | 1 | head-tail |
| P | forward | K | forward | 1 | head-tail |
| J | reverse | S | forward | 1 | tail-tail |
| Fp | reverse | Fp | forward | 1 | tail-tail |
| I | forward | Fp | forward | 1 | head-tail |
| O | reverse | Q | forward | 1 | tail-tail |
| E | reverse | V | forward | 1 | tail-tail |
| Mp | forward | E | forward | 1 | head-tail |
| J | forward | F | forward | 1 | head-tail |
| C | reverse | U | forward | 1 | tail-tail |
