# Supplementary material for: Small RNAs from mitochondrial genome recombination sites are incorporated into T. gondii mitoribosomes
Source: eLife. 2024 Feb 16;13:e95407. doi: 10.7554/eLife.95407 (PMC10948144; doi:10.7554/eLife.95407)
Supplement: Supplementary file 5. [file elife-95407-supp5.docx]

**Supplementary file 5: Fraction of reads containing full-length open reading frames.**

|  | **ORF length [nt]** | **#reads longer than ORF length** | **#reads containing full-length ORF** | **% reads containing ORF** |
| --- | --- | --- | --- | --- |
| **coxI** | 1,476 | 12,099 | 1,337 | **11.1** |
| **coxIII** | 745 | 28,503 | 1,463 | **5.1** |
| **cob** | 1,107 | 18,149 | 1,560 | **8.6** |
