# Supplementary material for: Small RNAs from mitochondrial genome recombination sites are incorporated into T. gondii mitoribosomes
Source: eLife. 2024 Feb 16;13:e95407. doi: 10.7554/eLife.95407 (PMC10948144; doi:10.7554/eLife.95407)
Supplement: Supplementary file 6. — After filtering the raw reads against the nuclear rRNA genes, the remaining reads were mapped against the three subgenomes of T. gondii RH-88. [file elife-95407-supp6.docx]

**Supplementary file 6: Mapping statistics of the RNA-seq data on the different genomes/sequence blocks.** After filtering the raw reads against the nuclear rRNA genes, the remaining reads were mapped against the three subgenomes of *T. gondii* RH-88.

| **Library** | **reads after rRNA filtering** | **Nuclear genome^1^** | **Apicoplast genome^2^** | **Mitochondrial sequence blocks^3^** | **Mitochondrial sequence blocks and combinations^4^** |
| --- | --- | --- | --- | --- | --- |
| Input1 | 14.402.531 | 8.380.729 | 27.627 | 4.182.533 | 4.814.603 |
| Input2 | 9.207.557 | 5.367.352 | 21.958 | 2.481.756 | 2.898.989 |
| Input3 | 16.001.184 | 7.791.619 | 34.156 | 5.866.849 | 6.905.255 |

1 from strain RH-88, accession number GCA_019455545.1

2 accession number CM033583.1

3 individual blocks according to [(Namasivayam et al. 2021; MN077088.1- MN077111.1)](https://paperpile.com/c/Byadca/Ldqeb/?suffix=%3B%20MN077088.1-%20MN077111.1)

4 All block combinations identified here as described in Fig. 2
