# Supplementary material for: Small RNAs from mitochondrial genome recombination sites are incorporated into T. gondii mitoribosomes
Source: eLife. 2024 Feb 16;13:e95407. doi: 10.7554/eLife.95407 (PMC10948144; doi:10.7554/eLife.95407)
Supplement: Supplementary file 7. [file elife-95407-supp7.docx]

**Supplementary file 7: List of mitochondrial non-coding RNAs identified by sRNA sequencing.**

| **Accession** | **Block** | **Assigned Name^1^** | **Position from RNA sequencing [Position previous annotation]** | **Length** | **assigned rRNA region^2^** | **Only found in cyst-forming Eucoccidians** |
| --- | --- | --- | --- | --- | --- | --- |
| MN077107.1 | U | **RNA15** | complement (10-36) | 27 |  |  |
|  |  | LSUA | 37 – 209 [37 – 209] | 173 | LSU1 |  |
|  |  | SSUA | 201 – 313 [204 – 314] | 113 | SSU4 |  |
| OR086911 | K | SSUB | complement (38-159)  [complement (38-156)] | 122 | SSU6 |  |
|  |  | **RNA29** | complement (163-204) | 42 |  | + |
|  |  | **RNA5** | complement (206-288) | 83 | SSU9 |  |
| MN077095.1 | I | **RNA9** | 23 – 97 | 75 | SSU8 |  |
|  |  | SSUF | 99 – 153  [complement (98-156)] | 55 | SSU12 |  |
| OR086913 | H | **RNA14** | complement (13-47) | 35 | SSU1 |  |
|  |  | LSUE | complement (52-331)  [complement (54-244; 247-331)] | 280 | LSU9 |  |
|  |  | LSUD |  |  | LSU8 |  |
|  |  | **RNA11** | complement (336-390) | 55 | LSU5 |  |
| OR086912 | M | LSUF | 280 – 485 [262-390; 381 – 487] | 206 | LSU11 |  |
|  |  | LSUG |  |  | LSU12 |  |
|  |  | SSUD | complement (523-588)  [complement (524-588)] | 66 | SSU10 |  |
| MN077106.1 | T | LSUB | complement (246-271)  [complement (247-271)] | 25 | LSU3 |  |
| MN077109.1 | Fp | **RNA28** | complement (44-85) | 42 |  | + |
| MN077102.1 | P | **RNA32** | 20 - 54 | 35 |  | + |
|  |  | **RNA33** | 55 - 125 | 71 |  | + |
| MN077100.1 | N | **RNA6** | complement (60-115) | 56 | LSU15 |  |
| MN077091.1 | D | **RNA30** | 14 – 44 | 31 |  | + |
| MN077102.1  +  MN077095.1 | P  +  I | **RNA7** | Block P 127-184  Block I 1-21 | 79 |  |  |
| MN077101.1  +  OR086910 | O  +  F | **RNA18** | Block O 69-86  Block F 1-13 | 31 | LSU14 |  |
| MN077101.1  +  MN077097.1 | O  +  K | RNA10 | Block O 47-86  Block K complement (291-333) | 83 | LSU13 |  |
| OR086912  +  MN077109.1 | M  +  Fp | SSUE | Block M 606-650  Block Fp complement (89-91) | 48 | SSU11 |  |
| MN077109.1  +  MN077100.1 | Fp  +  N | **RNA31** | Block Fp complement (1-20)  Block N 1-45 | 65 |  | + |
| MN077109.1  +  OR086910 | Fp  +  F | RNA8 | Block Fp complement (1-20)  Block F complement (2-88) | 107 | SSU5 |  |
| MN077091.1  +  MN077110.1 | D  +  Kp | **RNA2** | Block D 72-82  Block Kp 1-52 | 63 | LSU2 |  |
| MN077095.1  +  MN077110.1 | I  +  Kp | **RNA1** | Block I 157-204  Block Kp 1-48 | 96 | LSU6 |  |
| MN077107.1  +  MN077110.1 | U  +  Kp | **RNA13** | Block U complement (1-8)  Block Kp complement (87-112) | 34 | LSU10 |  |
| OR086911  +  MN077110.1 | K  +  Kp | **RNA17** | Block K complement (1-33)  Block Kp complement (105-112) | 41 | SSU3 |  |
| OR086915  +  OR086913 | Q  +  H | **RNA16** | Block Q complement (1-18)  Block H complement (390-397) | 26 |  |  |
| MN077107.1  +  OR086916 | U  +  B | **RNA23t** | Block U 312-354  Block B complement (127-144) | 61 |  |  |
| MN077088.1  +  MN077100.1 | A  +  N | **RNA34** | Block A complement (1-13)  Block N complement (117-166) | 63 |  |  |
| MN077105.1  +  MN077104.1 | S  +  R | **RNA3** | Block S 184-205  Block R complement (17-85) | 91 | LSU7 |  |
| MN077096.1  +  MN077092.1 | J  +  E | **RNA19** | Block J = 74-85  Block E = 1-22 | 34 | SSU7 |  |

1 RNAs in bold have not been previously predicted based on sequence similarities [1]
2 Numbers specify the linear order of fragments relative to conventional rRNA as suggested by Feagin *et al.* 2012 [2]
(LSU: ribosomal large subunit, SSU: ribosomal small subunit)
