# Supplementary material for: Small RNAs from mitochondrial genome recombination sites are incorporated into T. gondii mitoribosomes
Source: eLife. 2024 Feb 16;13:e95407. doi: 10.7554/eLife.95407 (PMC10948144; doi:10.7554/eLife.95407)
Supplement: Supplementary file 8. [file elife-95407-supp8.docx]

|  | *P. falciparum** | | *T. gondii* | |
| --- | --- | --- | --- | --- |
|  | number of fragments | nt total | number of fragments | nt total |
| LSU rRNA | 15 | 1233 | 12^$^ | 1193 |
| SSU rRNA | 12 | 804 | 11 | 845 |
| unassigned sRNAs shared^1^ | 4 | 205 | 4 | 193 |
| unassigned sRNAs unique^2^ | 8 | 350 | 7 | 349 |

**Supplementary file 8: Overview of mitochondrial non-coding RNAs identified in *P. falciparum* and *T. gondii***

* based on Feagin *et al.,* 2012 and Hillebrand *et al.,* 2018
^$^ Please note that LSUE and D as well as LSUF and G were shown to be single rRNAs here, thus reducing the number of fragments in *T. gondii*
1 sRNAs not assigned to a region of ribosomal RNA, found in *P. falciparum* and *T. gondii*
2 sRNAs not assigned to a region of ribosomal RNA, only found in *P. falciparum* or *T. gondii*
