# Supplementary material for: Small RNAs from mitochondrial genome recombination sites are incorporated into T. gondii mitoribosomes
Source: eLife. 2024 Feb 16;13:e95407. doi: 10.7554/eLife.95407 (PMC10948144; doi:10.7554/eLife.95407)
Supplement: Supplementary file 9. [file elife-95407-supp9.docx]

**Supplementary file 9: List of oligonucleotides used in this study**

| **Name** | **Sequence (5' to 3')** | **ID^*^** |
| --- | --- | --- |
| RNA17 probe | GACTTATTAAACCAGCCTGGGATCA | a |
| RNA29 probe | GTTCTAATTCCCCGTGGTAAACACAGTC | b |
| RNA5 probe | GTGTCATAGGAGATATACTCTATAATTAG | c |
| LSUE probe | GCCATCTCGATCCTCATATTCAAA | d |
| LSUD probe | GCGGTATCTAGTGTTGATGTACAA | e |
| LSUF probe | GAACTCCCAATATTATCTGACCTGTT | f |
| LSUG probe | GTATTACATCTGACGGTGAACTATC | g |
| RNA6 probe | GTCTCTCAATAACTAGCTGAGTGCTTG | h |
| RNA7 probe | GTTTCATAAATACAATCAGTGAAAGCTCTT | i |
| RNA1+RNA2 probe | GTTTGTACCTACTTGACTCCTCAGTTTAAG | j |
| RNA34 probe | GCATTTCCTATGCTCCTTAACATCACAGC | k |
| RNA19 probe | GTTCTCGAAACCATGCTAACACAATAG | l |
| RNA3 probe | GCTCACTGCGTACTTAGGATCAGACCAA | m |
| SSUA probe | GACTCTTCTATAGTTTAACCGCTACTG |  |
| SSUD probe | GGCGCTTAATAACGATTCCGTC |  |
| Cob RACE PCR primer | CCCCAGAAACTCATTTGTCCCC |  |
| Rumsh1 RACE PCR primer | TGATCCAACCGACGCGAC |  |
| Rumsh 5’ RACE linker | GUGAUCCAACCGACGCGACAAGCUAAUGCAAGANNN (RNA) |  |
| cox3 qPCR fwd | CAGTACCATCGTACTGGGAGTAATC |  |
| cox3 qPCR rev | GATAGACCTAAGTATTCCGTACAGAC |  |
| cob qPCR fwd | CGCGCTTAAAGTTGCCTTTTATC |  |
| cob qPCR rev | GCTCGAATCTCAGAAAGTAAACC |  |
| cox1 qPCR fwd | GAGTTATACAGTTCTGGTTCGC |  |
| cox1 qPCR rev | CACTACCAAATTCAGCACAAATAC |  |
| LSUF/G qPCR fwd | GTCGGTGCTATCTTGGGTTTC |  |
| LSUF/G qPCR rev | CCTGTTATCCCCGGCGTACCTTAC |  |
| ESR1 qPCR fwd | CCAGATGGTCAGTGCCTTGT |  |
| ESR1 qPCR rev | CAAATCCACAAAGCCTGGCA |  |
| 3x HA amplification fwd – (+HindIII restriction site) | TATAAAGCTTGGTGGAGGTAGCGGTGGTGGAAGTT  ACCCGTACGACGTCCCG |  |
| 3x HA amplification rev – (+NsiI restriction site) | TATAATGCATATTATGCGCAGGCATAATCTGGAACA  TCGTAAGG |  |
| Q5 mutagenesis gRNA fwd Tgurpl11m tagging | GTCACAGTCACTTCTTTGTGGGTTTTAGAGCTAGA  AATAGC |  |
| Q5 mutagenesis rev Tgurpl11m tagging | AACTTGACATCCCCATTTAC |  |
| flank fwd  Tgurpl11m tagging | AAGACATGCGTCAGAGAAAGAAAGCAGCGAAGCGA  GCGGCCACAAAGAAGGGTGGAGGTAGCGGTGGTG  GAAGT |  |
| flank rev  Tgurpl11m tagging | TCTTGAAACAGACGGGAAGGCAAAAAGAACAAAAA  AACCACACTTCACAGTGCAGGGCTCTAGAACTAGT  GGATCG |  |
| genotyping fwd  Tgurpl11m tagging | TTGCCTGCGTACCACAAGC | P1 |
| genotyping rev  Tgurpl11m tagging | CAGGAGAAAGCCAAGCGGA | P2 |

* lowercase letter used as probe IDs or numbers used as primer IDs in figures, respectively
